# Clinical Manifestations of Alport Syndrome-Diffuse Leiomyomatosis Patients With Contiguous Gene Deletions in *COL4A6* and *COL4A5*

**DOI:** 10.3389/fmed.2021.766224

**Published:** 2021-10-27

**Authors:** Xi Zhou, Jingjing Wang, Jianhua Mao, Qing Ye

**Affiliations:** The Children's Hospital of Zhejiang University School of Medicine, National Clinical Research Center for Child Health, Hangzhou, China

**Keywords:** X-linked Alport syndrome, diffuse leiomyomatosis, *COL4A5*, *COL4A6*, gene deletion, DNA analysis

## Abstract

Alport syndrome-diffuse leiomyomatosis is a rare type of X-linked Alport syndrome resulting from contiguous deletions of 5′ exons of *COL4A5* and *COL4A6*. Studies have suggested that the occurrence of diffuse leiomyomatosis is associated with the characteristic localisation of the *COL4A6* gene deletion break point. An electronic database was searched for all studies accessing AS-DL to analyze the clinical characteristics, gene deletion break points of patients with AS-DL, and the pathogenesis of AS-DL. It was found that the proportion of *de novo* mutations of AS-DL was significantly higher in female probands than male probands (78 vs. 44%). Female patients with AS-DL had a mild clinical presentation. The incidence of proteinuria and ocular abnormalities was much lower in female probands than in male probands, and there was generally no sensorineural hearing loss or chronic kidney disease (CKD), which progressed to Stage 3 in female probands. The contiguous deletion of the 5' exons of *COL4A5* and *COL4A6*, with the break point within the intron 3 of *COL4A6*, was the critical genetic defect causing AS-DL. However, the pathogenesis of characteristic deletion of *COL4A6* that contributes to diffuse leiomyomatosis is still unknown. In addition, characteristic contiguous deletion of *COL4A5* and *COL4A6* genes in AS-DL may be related to transposed elements (TEs).

## Introduction

Alport syndrome is a hereditary glomerular disease characterised by hematuria, progressive nephritis, sensorineural hearing loss, and ocular abnormalities (1, 2). AS is mainly caused by mutation of the gene-encoding type IV collagen in the glomerular basement membrane. Abnormal expression of type IV collagen leads to irregular thickening and thinning of glomerular basement membrane (GBM), lamellation and splitting in lamina densa, podocyte disappearance, glomerulosclerosis with extracellular matrix deposition, renal fibrosis, and finally leads to end-stage renal disease (ESRD). Alport syndrome is a heterogeneous disease with three modes of inheritance, including X-linked Alport syndrome (XLAS), autosomal recessive AS (ARAS), and autosomal AS (ADAS). X-linked Alport syndrome (XLAS, OMIM: 301050) is the most common disease form, accounting for about 80–85% (1, 3). Extensive abnormalities of glomerular basement membrane (GBM) in patients with AS were observed under the electron microscope, including irregular thinning, thickening, and lamellation, and splitting in lamina dense (2). Absence or incomplete expression of type IV collagen α5 chain can be observed in kidney tissue of patients with XLAS. XLAS is caused by mutation of the *COL4A5* gene-encoding type IV collagen α5 chain (4). Mutations include missense mutations, splice site mutations, truncating mutations, and deletion mutations. These mutations are spread throughout the gene without any identified mutational hot spot. *COL4A5* is located on the X chromosome. Therefore, male patients with hemizygous mutation usually have severe clinical manifestations. Female heterozygote carriers may have a wide range of symptoms, from isolated hematuria to ESRD (5).

*COL4A6* gene is paired with *COL4A5* head-to-head, located on chromosome Xq22.3, encoding collagen α6 chain, usually expressed in Bowman's capsule, epidermis, and smooth muscle cells (6). Studies have shown that rare XLAS families, patients with contiguous deletion of *COL4A5* gene, and *COL4A6* gene will be associated with diffuse leiomyomatosis (DL). DL is a benign smooth muscle tumour characterised by abnormal proliferation of well-differentiated smooth muscle cells involving the gastrointestinal tract, tracheobronchial, and female reproductive tract (7). Clinical symptoms commonly include progressive dysphagia, vomiting, or dyspepsia; less frequent, retrosternal pains, dyspnea, cough, or weight loss. Alport syndrome-diffuse leiomyomatosis (AS-DL, OMIM: 308940) is a rare variant of the X-linked Alport syndrome. Up to now, it has been reported that about 30 AS-DL families were found to carry a characteristic contiguous deletion of *COL4A5* and *COLA6* (8, 9). However, not all kinds of deletion of the *COLA6* gene will cause DL (10). This study reports an XLAS case with complete *COL4A6* gene deletion. At the same time, through the summary of reported clinical cases and literature review of related basic research results, the break point of the deletion of *COL4A6* gene in patients with AS-DL was identified, and the hypothesis that caused characteristic contiguous deletion of *COL4A5* and *COL4A6* gene and the possible pathogenesis of DL caused by *COL4A6* gene deletion were summarised.

## Patients and Methods

### Subjects and Clinical Assessment

The proband (III-3) in the pedigree was admitted with proteinuria and haematuria to the Department of Nephrology, The Children's Hospital of Zhejiang University School of Medicine. Clinical information of the family members of the patient was collected during interviews; this included age, gender, symptoms, previous history of the disease, and positive test results. Blood samples from the proband and his mother were collected for genetic screening. Written informed consent was obtained from all the participants before enrolment.

### DNA Extraction

The manufacturer extracted genomic DNA from 5 ml of peripheral blood collected from the patient with XLAS and his family members using a QIAamp Blood DNA Mini Kit (Qiagen®, Milano, Italy) instructions. DNA concentrations were determined using a NanoDrop spectrophotometer (Thermo Scientific®, Waltham, USA). DNA samples were stored at −20°C until use.

### Whole-Exome Sequencing

Array capture was used to enrich relevant human genes (SeqCap EZ Human Exome Library v2.0, Roche®, Basel, Switzerland), sequenced using the Illumina HiSeq 2000 platform (2016 Illumina, Inc. USA).

### Filtering Data

The following steps were taken to prioritise high-quality variants: (i) variants within intergenic, intronic, and untranslated regions (UTRs) and synonymous mutations were excluded from downstream analysis; (ii) variants with a quality score below 20 were excluded; (iii) only a conservation score (phyloP) above three based on comparison of humans and 43 vertebrates was considered; (iv) after this prior selection, the remaining genes were filtered by the function. PolyPhen-2 software (http://genetics.bwh.harvard.edu/pph2/) predicted the possible impact of variants. The final set of selected variants was visually inspected using Integrative Genomics Viewer. Previously described polymorphic variants in public data were investigated and compared with the variations found in the exome of the proband. The selected mutations investigated in this study were not found in previous exome sequences (http://evs.gs.washington.edu/EVS/).

### Sanger Sequencing Validation

Sanger sequencing was used to confirm next-generation sequencing of all subjects. DNA from the patient and his family members was subjected to PCR analysis. Polyacrylamide gel electrophoresis was used to determine the size of the amplification products, which were purified using a QIA quick PCR purification kit (Qiagen®, Milano, Italy) and then sequenced using both forward and reverse primers with ABI BigDye Terminator Cycle Sequencing Kit v. 3.1 and an ABI PRISM 3730XL Genetic Analyzer (Applied Biosystems®, Foster City, USA). The results were aligned with the reference sequence, and mutations were identified using sequencer software (http://www.genecodes.com). The deletions of the *COL4A5* gene and *COL4A6* gene exons were confirmed by qPCR, PCR, and gel electrophoresis. All primers were designed using the online tool primer 3. The primers of each exon of the *COL4A5* gene and *COL4A6* gene are shown (Supplementary Table 1), respectively. The fluorescence quantitative PCR reaction system of *COL4A5* gene exon detection is as follows: 2^*^ KAPA SYBR Fast qPCR Master Mix Universal 10 μl, 10-μM F Primer 0.4 μl, 10-μM R Primer 0.4 μl, DNA templates 1 μl, ROX High 0.4 μl, PCR-grade water 7.8 μl. The reaction system of *COL4A6* gene exon detection is as follows: 5 × buffer A 5 μl, 10-mm dNTP 0.5 μl, 10-m f primer 1.25 μl, 10-m r primer 1.25 μl, DNA templates 1 μl, 0.1 μl DNA polymerase (kapa2g robust hot start PCR kit), 15.9-μl PCR-grade water. PCR cycle conditions: 95°C 3 min, 95°C 3 s, 60°C 30 s, 40 cycles.

## Result

### An XLAS Family With Contiguous Deletion of the *COL4A5* and *COL4A6*

**Figure 1:**
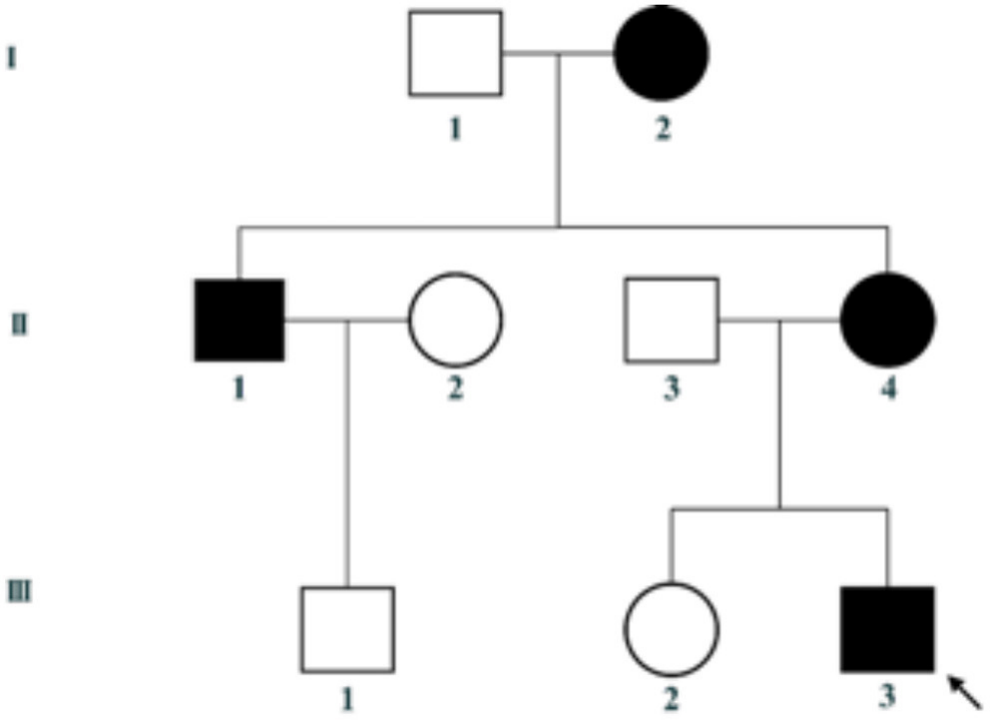
Pedigree of the family. III-3 is the proband.

**Figure 2:**
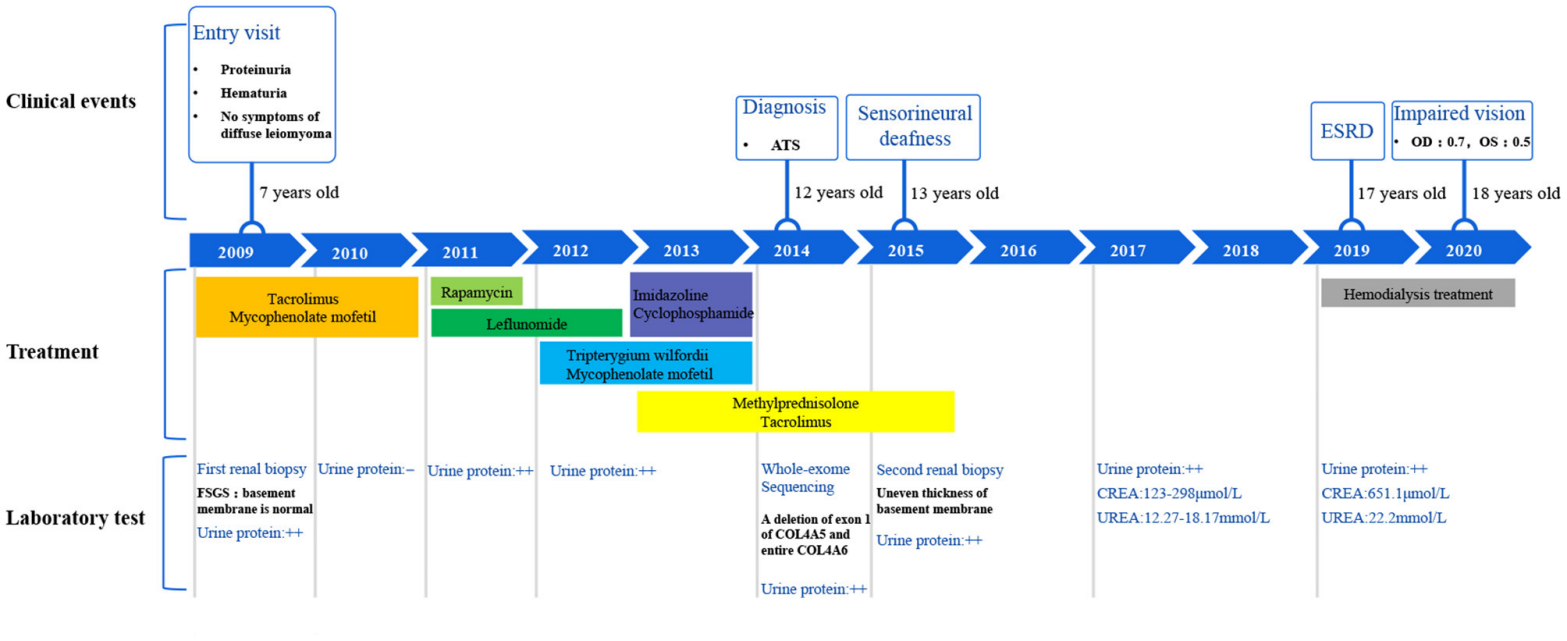
Disease progression of the proband.

**Figure 3:**
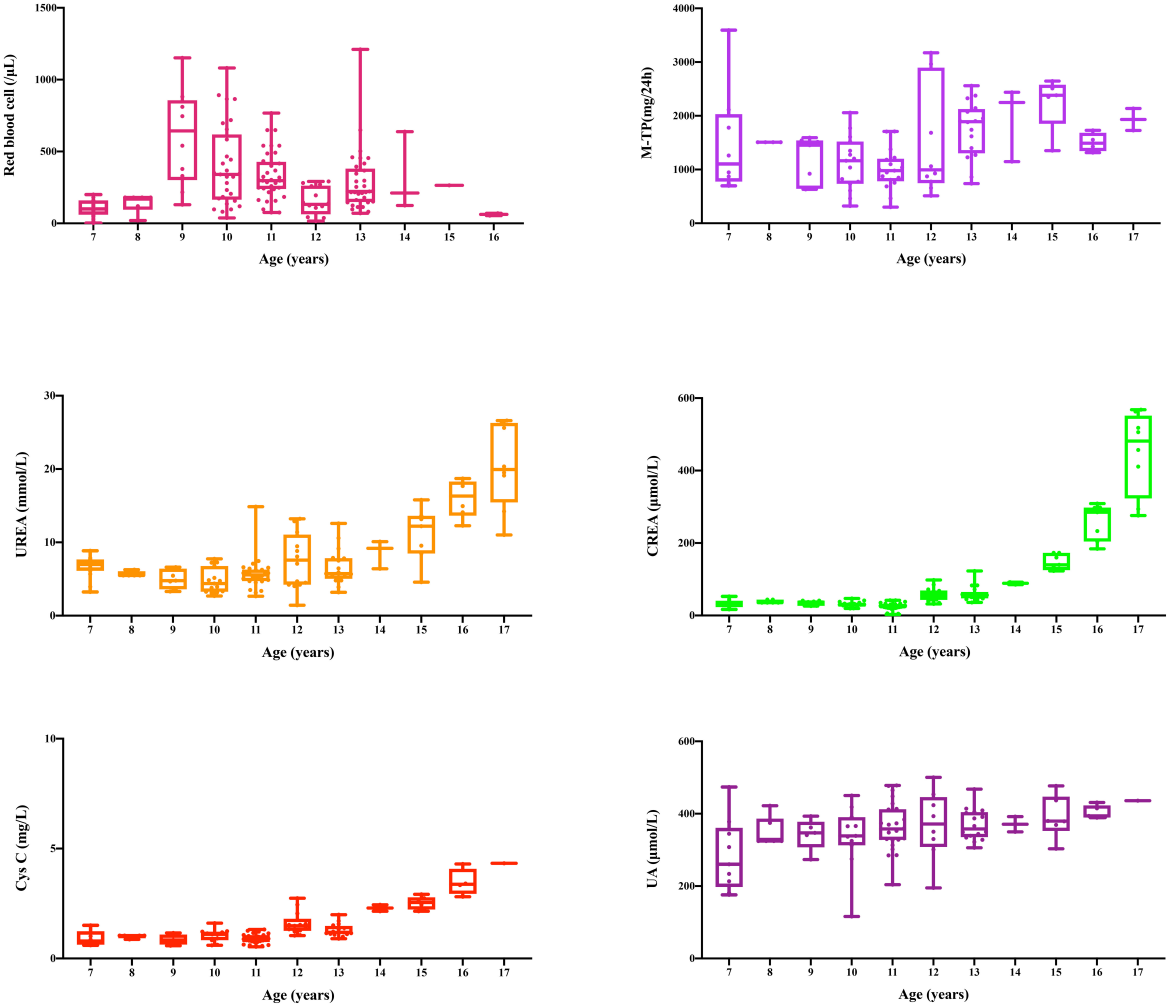
Changes of laboratory indexes during 11 years from diagnosis to progression to uremia.

**Figure 4:**
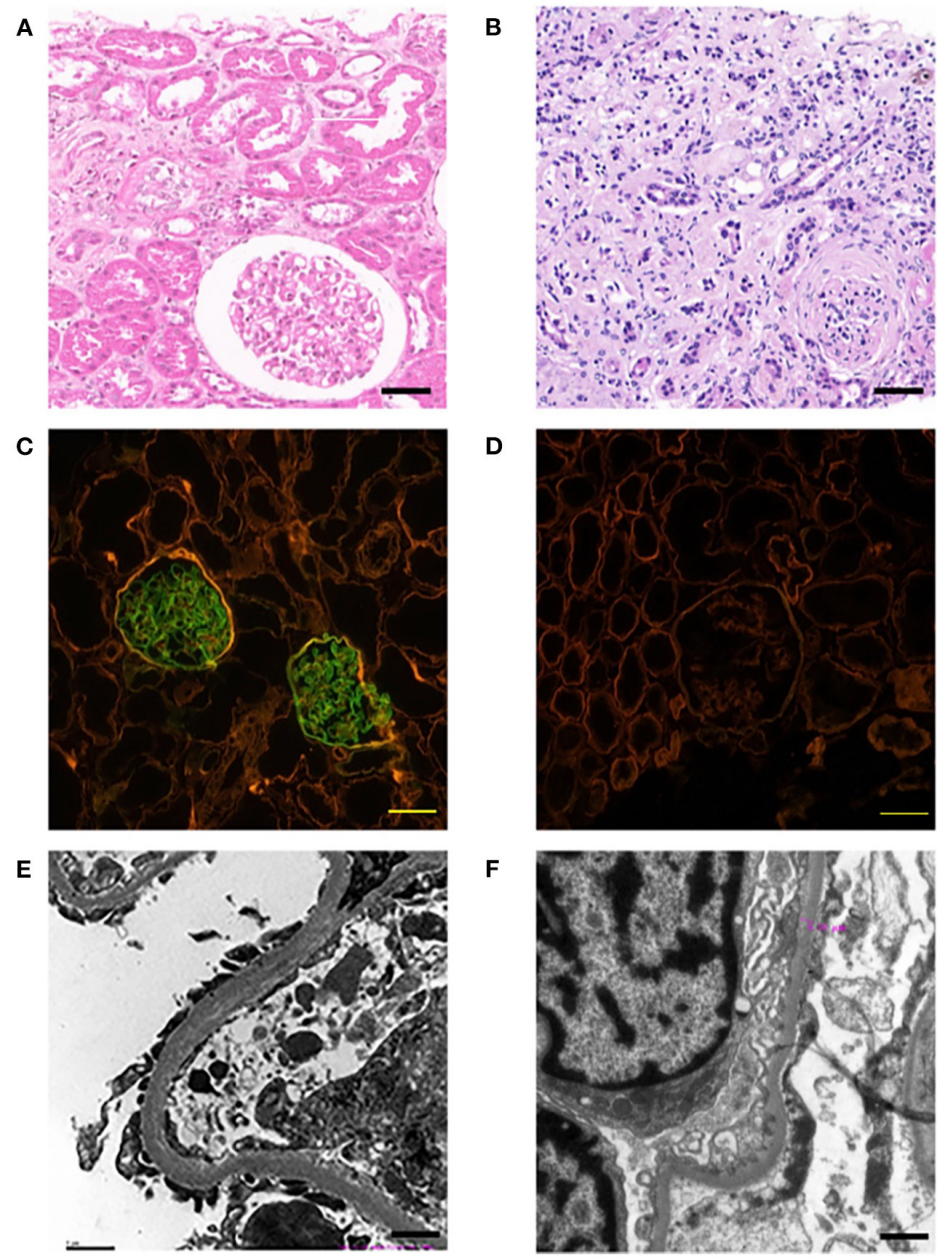
Pathological examination results of probands. The left panel **(A,C,E)** show the results of the first renal biopsy (March 10, 2009), and the right panel **(B,D,F)** show the results of the second renal biopsy (August 28, 2015). **(A)** HE staining is characterised by FSGS. Scale bars, 100 μm. **(B)** HE staining is characterised by AS. Scale bars, 100 μm. **(C)** Immunofluorescence staining of α5 chain (IV) showed that the expression of α5 chain was normal; the immunofluorescence staining was positive, which emitted bright green fluorescence by a confocal laser scanning microscope. Scale bars, 20 μm. **(D)** Immunofluorescence staining of α5 chain (IV) showed the absence expression of α5 chain; the immunofluorescence staining was negative, which showed no emitted bright green fluorescence by a confocal laser scanning microscope. Scale bars, 20 μm. **(E)** The electron microscope examination showed that the basement membrane was normal. Scale bars, 1 μm. **(F)** The electron microscope examination showed that the glomerular basement membrane was uneven in thickness, about 200–500 nm. Scale bars, 1 μm.

This family includes an 18-year-old male proband (III-1), his mother (II4), his maternal uncle (II1), and maternal grandmother (I2) (Figure 1). This study recorded the progress of the disease (Figure 2) and the changes in the laboratory evaluation of the renal function of the proband (Figure 3). Urinary abnormalities include proteinuria and hematuria, were detected in the proband at the age of 7 and diagnosed as focal segmental glomerular sclerosis (FSGS) based on the first renal biopsy. The immunofluorescence staining showed normal expression of α5 chain, which was positive in GBM, and the electron microscopic analysis revealed diffuse fusion of the foot process, normal thickness of basement membrane, and an increased mesangial matrix of GBM of the proband (Figure 4). The therapeutic regimen of mycophenolate mofetil (MMF) and tacrolimus (TAC) was used to treat FSGS in the proband from 2009 to 2010. Urine protein turned negative in 2010 when the proband was 8 years old. However, urine protein turned positive in 2011, and repeated use of various immuno suppressants was ineffective. At age 13, he was diagnosed with XLAS based on genetic analysis, typical immunofluorescence staining, and electron microscope findings on a second renal biopsy. The immunofluorescence staining showed negative expression of α5 chain, and the electron microscopic analysis showed irregular thickening of the GBM, with splitting and fragmentation of the lamina densa (Figure 4). High-frequency sensorineural hearing loss was diagnosed at the same age. The proband reached ESRD at 17 years old. Ocular abnormalities (OD: 0.7, OS: 0.5) have been presented since the proband was 18 years old. The proband has no symptoms that might be suggestive of DL as he was never reported a history of gastrointestinal or tracheobronchial symptoms, such as dysphagia, postprandial vomiting, retrosternal or epigastric pain, recurrent bronchitis, dyspnea, cough, and stridor. In addition, the mother of the proband, diagnosed with XLAS, has had proteinuria and hypertension since 15 years old. In addition, his mother has no evidence of CKD and DL. The maternal grandmother of the proband has transient (intermittent) proteinuria and no evidence of CKD and DL. The maternal uncle of the proband presented with urinary abnormalities and eventually reached ESRD at age 27 years. The maternal uncle of the proband also shows no DL symptoms.

**Figure 5:**
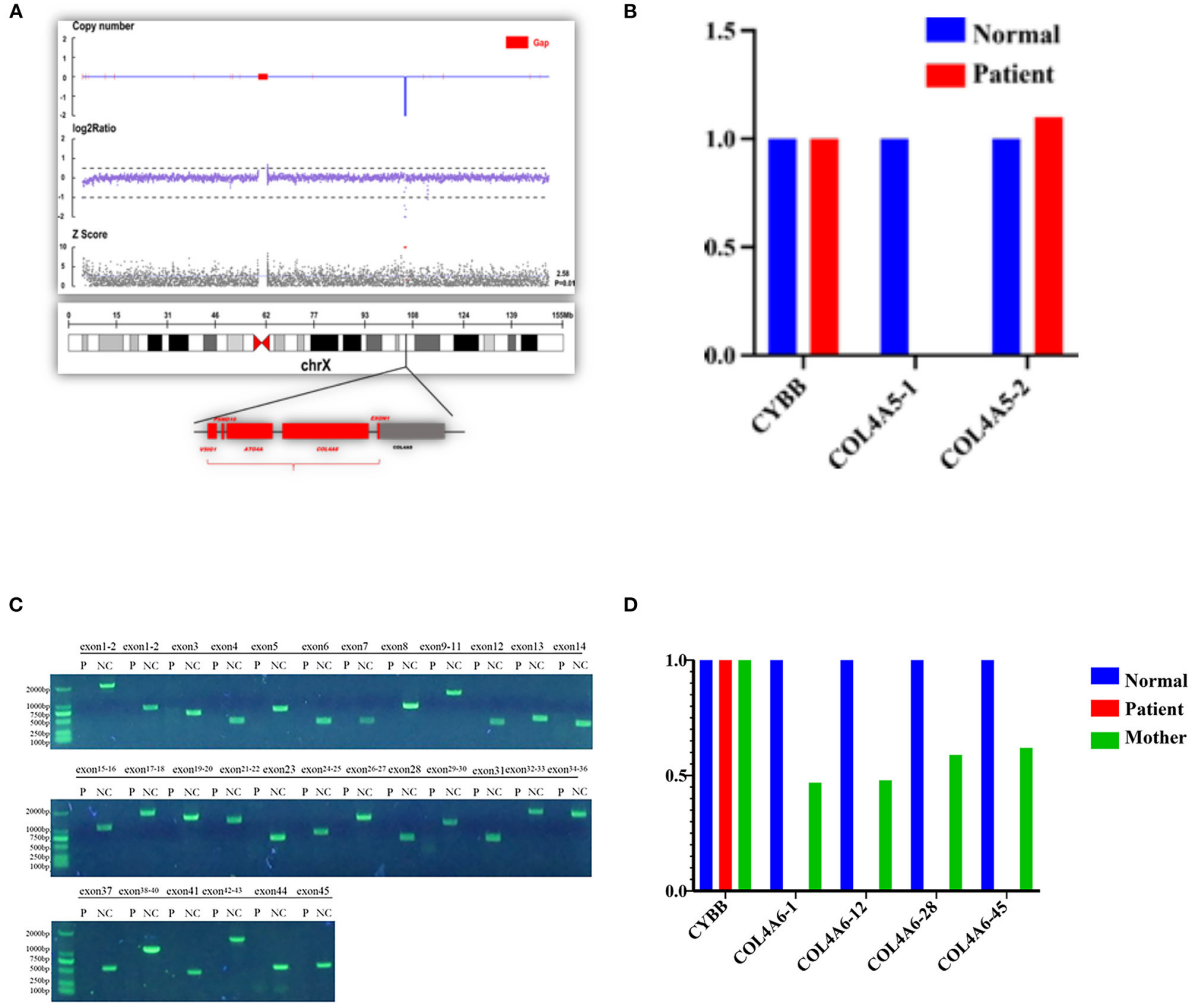
Contiguous gene deletion of *COL4A5* and *COL4A6* in the AS case we reported. **(A)** Whole exon sequencing showed that the *COL4A5*-*COL4A6* gene was contiguously deleted on the X chromosome of the proband. **(B)** qPCR verification of the exon deletion break point of *COL4A5* gene of the proband. The first exon of the *COL4A5* gene was deleted, and the copy of the second exon was normal. **(C)** Verification of the exon deletion break point of *COL4A6* gene of the proband. Amplifying each exon for the electrophoresis result of PCR in the same group of the normal control sample and the proband sample of the *COL4A6* gene. The fragment size is 1,986 bp, 643 bp, 1,560 bp, 376 bp, 714 bp, 1,354 bp, 568 bp, 796 bp, 1,402 bp, 383 bp, 509 bp, 466 bp, 1,078 bp, 1,776 bp, 1,473 bp, 1,278 bp, and 611 bp. The amplification results of control genes of the proband and normal control samples have obvious specific bands, and the product length is consistent with the design length. It is suggested that the proband *COL4A6* gene may be homozygous deletion. **(D)** qPCR verification of the exon deletion break point of *COL4A6* gene of the proband and mother of the proband. The normal control samples, proband samples, and maternal samples were detected by fluorescence quantitative PCR in the same group, and the copy numbers of exons 1, 12, 28, and 45 (represented by *COL4A6*-1, *COL4A6*-12, *COL4A6*-28, and *COL4A6*-45, respectively) of the target gene were detected by using ALB gene as internal reference gene. The results showed that the *COL4A6* gene of the proband had no amplification signal. That is, no copy of the gene was detected. The ratio of the copy number of each exon of the *COL4A6* gene in the maternal sample to the normal control is about 0.5, suggesting that the proband has a homozygous deletion of the *COL4A6* gene and the maternal sample has heterozygous deletion.

In order to identify a molecular cause underlying the clinical features of the patient, a genetic analysis of the proband and his mother was conducted. The deletion of entire exons of *COL4A6* and exon 1 of *COL4A5* was detected in the proband by whole-exome sequencing and confirmed by PCR. Genetic testing of blood DNA from the mother of the proband also showed the heterozygous deletion of all exons of *COL4A6* and exon 1 of *COL4A5* (Figure 5). The father of the proband did not have AS symptoms and carry the mutation of *COL4A5* or *COL4A6*. Co-segregation was observed in this family.

### Clinical Phenotypes of AS-DL Probands With Contiguous Deletion of the *COL4A5* and *COL4A6*

**Figure 6:**
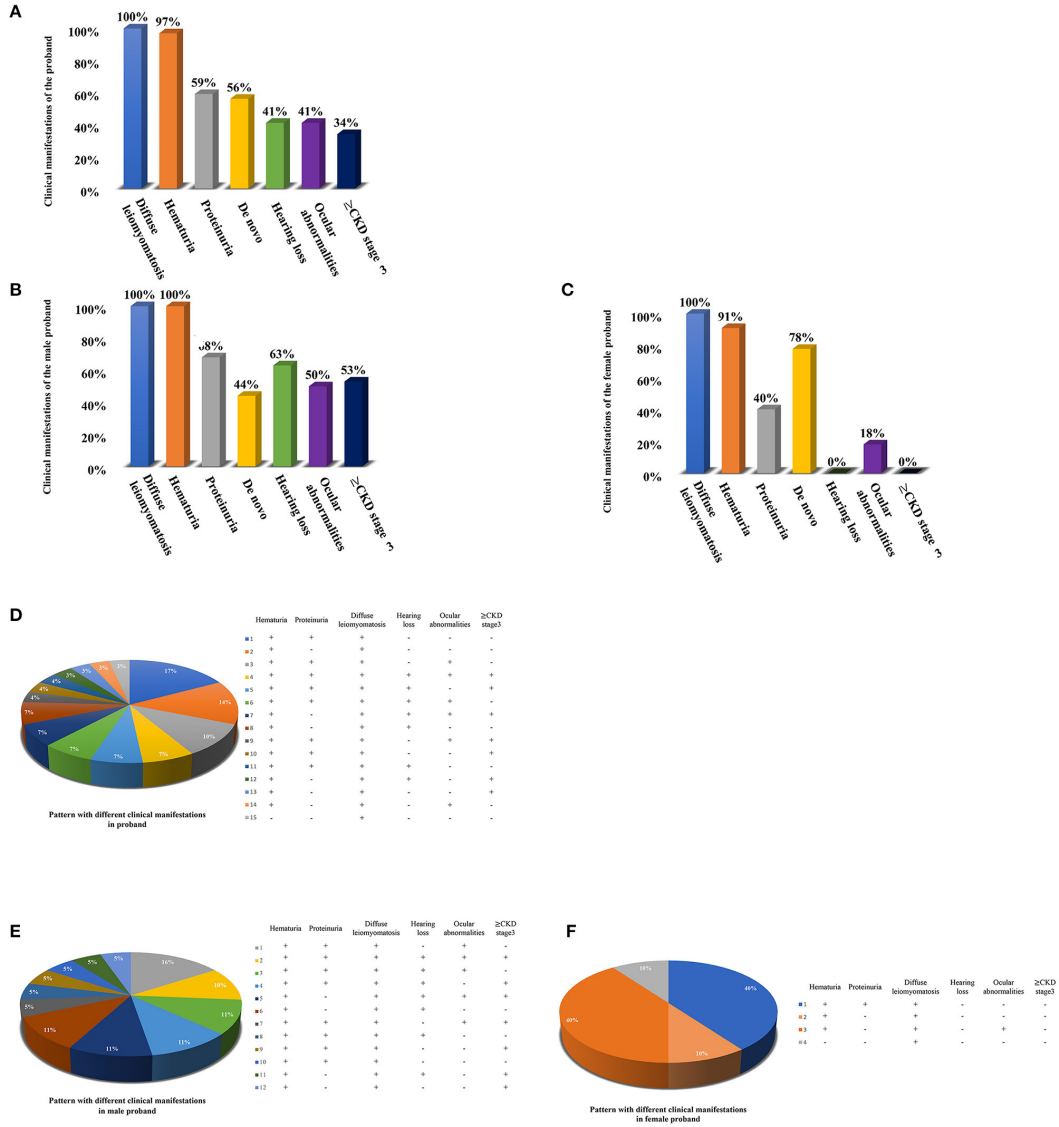
Clinical manifestations of the AS-DL proband and the pattern with different clinical manifestations. **(A)** Clinical manifestations of the total AS-DL proband. **(B)** Clinical manifestations of the male AS-DL proband. **(C)** Clinical manifestations of the female AS-DL proband. **(D)** The pattern with different clinical manifestations. **(E)** The pattern with different clinical manifestations in male probands. **(F)** The pattern with different clinical manifestations in female probands.

Totaling 15 relevant references were identified by searching the database of PubMed to analyse the clinical characteristics and gene deletion break points of patients with AS-DL (Table 1 and Figures 6A–C). Thirty-one probands had been retrieved, including 11 females and 20 male probands whose ratio of males to females is 1:.55. The initial clinical manifestation related to AS-DL appeared in early childhood, with a median age of 3 years and a general age of no more than 13 years. Among them, the median age of the male proband who had an initial clinical manifestation of AS-DL was 3 years old, while that of the female proband was later, with a median age of 6 years old. Many AS-DL probands have a family history. However, about 56% of probands were *de novo*. The DL of these probands occurred before the age of 25, and the median age of developing DL was 9 years old. There is no significant difference in the occurrence time of DL between different sexes. Among them, the median age of the male proband developing DL was 9.5 years old, and that of female patients was 9 years old. All probands analysed in the article were diagnosed with oesophageal leiomyomas. Oesophagus leiomyomas are a constant finding in families with AS-DL, the initial clinical manifestation in most patients. A few probands were diagnosed as rectal involvement by DL. Only a few probands had respiratory symptoms (dyspnea, wheezing, asthma, and bronchitis). In addition, several female probands had genital leiomyomas with diffuse clitoral and vulvar hypertrophy. Except for DL, the incidence of other clinical symptoms in patients with AS-DL from high to low was hematuria, proteinuria, sensorineural hearing loss, and ocular abnormalities. Hematuria was one of the most common clinical manifestations, which usually occurs earlier, and the incidence of male probands was higher than that of female probands. Hematuria occurred in 97% of patients and was observed in all these male probands. The median onset age of hematuria was 4.5 years. About 91% of female probands also developed hematuria, and their median age of hematuria was 3 years old. Proteinuria was also very common in these probands. About 59% of probands have proteinuria, among which the incidence of proteinuria in male probands was 68%, while that in female probands was about 40%. There was no difference in the median age of proteinuria between male and female probands, and all were 6 years old.

**Table 1:** Clinical and genetic characterisation of patients with Alport syndrome−diffuse leiomyomatosis.

**Proband**	**Gender**	**Age (onset of symptoms)**	**Age**	**Phenotype**							**GBM**	**Intron deletion**	**Genotype Deletion**		***de novo***	**References**
				**Hematuria**	**Proteinuria**	**Hypertension**	**≥CKD stage3**	**Diffuse leiomyo-matosis**	**Hearing loss**	**Ocular abnormalities**			***COL4A5***	***COL4A6***		
1	M	6	19	+(6)	−	−	+	+(8)	−	−	+	Del int.1[*COL4A5*]~del int.2[*COL4A6*]	EX.1	EX.1_2	Y	(11)
2	M	6	30	+(6)	−	−	+(16)	+(21)	+ (24)	+ (21)	+	Del int.1[*COL4A5*]~del int.2[*COL4A6*]	EX.1	EX.1_2	NA	(12)
3	M	11	29	+(11)	−	−	+(25)	+(17)	+(>20)	+(>20)	+	Del int.1[*COL4A5*]~del int.2[*COL4A6*]	EX.1	EX.1_2	N	(12)
4	F	10	13	+(10)	+(10)	−	−	+(10)	−	−	+	Del int.1[*COL4A5*]~del int.2[*COL4A6*]	EX.1	EX.1_2	Y	(13)
5	F	9	23	−	−	−	−	+(9)	−	−	+	Del int.1[*COL4A5*]~del int.2[*COL4A6*]	EX.1	EX.1_2	NA	(14)
6	M	2	26	+(2)	+(7)	+	+	+(25)	+(6)	+(26)	+	Del Int.2~int.51[*COL4A5*]	EX.2−51	/	N	(10)
7	M	13	15	+(15)	+(15)	−	+(15)	+(13)	−	−	+	Del int.1[*COL4A5*]~del int.2[*COL4A6*]	EX.1	EX.1_2	N	(15)
8	M	7	10	+(7)	+(9)	−	−	+(9)	−	−	+	Del int.1[*COL4A5*]~del int.2[*COL4A6*]	EX.1	EX.1_2	Y	(16)
9	F	3	11	+(3)	+(6)	−	−	+(7)	−	−	+	Del int.1[*COL4A5*]~del int.2[*COL4A6*]	EX.1	EX.1_2	N	(16)
10	F	1	15	+(1)	+(3)	−	−	+(6)	−	−	+	Del int.36[*COL4A5*]~del int.2[*COL4A6*]	EX.1_36	EX.1_2	Y	(16)
11	M	1	10	+(1)	+(5)	−	−	+(10)	+	−	+	Del int.4[*COL4A5*]~del int.2[*COL4A6*]	EX.1_4	EX.1_2	N	(16)
12	M	5	38	+(5)	+(5)	−	ESRD (30)	+(11)	+	−	+	Del int.1[*COL4A5*]~del int.2[*COL4A6*]	EX.1	EX.1_2	N	(16)
13	M	5	25	+(11)	−	−	ESRD (25)	+(5)	+	−	+	/	EX.1	EX.1−2	NA	(17)
14	M	2	16	+(20)	−	−	−	+(12)	+	−	+	/	EX.1	EX.1−2	NA	(17)
15	M	13	40	+	+	−	ESRD(40)	+(13)	+	+	+	/	EX.1	EX.1−2	N	(18)
16	M	3	20	+(4)	+	−	+	+(3)	+(6)	−	+	/	EX.1−36	EX.1−2	Y	(19)
17	M	<11	33	+	+	−	−	+(14)	+(11)	+(11)	+	/	EX.1	EX.1−2	N	(19)
18	M	<1	9	+(2)	−	−	−	+(<1)	+(9)	−	+	/	EX.1−7	EX.1−2	Y	(20)
19	F	9	25	+	−	−	−	+(9)	−	+	+	/	EX.1	EX.1−2	Y	(21)
20	M	1	1	+(1)	+(1)	−	−	+(1)	−	+(1)	+	/	EX.1	EX.1−2	N	(22)
21	F	NA	NA	+	−	−	−	+	−	−	NA	/	EX.1	EX.1−2	Y	(23)
22	F	NA	NA	+	−	−	−	+	−	−	NA	/	EX.1	EX.1−2	Y	(23)
23	F	NA	NA	+	−	−	−	+	−	−	NA	/	EX.1−30	EX.1−2	Y	(23)
24	F	NA	NA	+	−	−	−	+	−	−	NA	/	EX.1−2	EX.1−2	Y	(23)
25	M	3	24	+(3)	+	−	ESRD (16)	+(6)	−	+(6)	+	/	EX.1−3	EX.1−2	Y	(24)
26	M	2	7	+(2)	+(7)	−	−	+(7)	−	+(4)	+	/	EX.1	EX.1−2	Y	(24)
27	M	1	17	+(1)	+(4)	−	−	+(9)	+(17)	+(2)	+	/	EX.1	EX.1−2	N	(24)
28	M	NA	NA	+	NA	NA	NA	+	NA	−	NA	/	EX.1−2	EX.1−2	NA	(24)
29	M	1	14	+	+	−	−	+	−	+(1)	+	/	EX.1	EX.1−2	Y	(24)
30	F	2	5	+	+	−	−	+	−	−	+	/	EX.1	EX.1−2	N	(24)
31	F	NA	NA	+	NA	NA	NA	+	NA	+	NA	/	EX.1	EX.1−2	NA	(24)

*GBM, glomerular basement membrane; CKD, chronic kidney disease; F, female; M, male; Del, deletion; NA, data not available; Y, yes; N, no; EX, exon; age at the onset of the phenotype was placed in parentheses*.

Sensorineural hearing loss usually occurs in older children. About 41% of the probands were found to have sensorineural hearing loss, and they were all male patients with a median age of 11 years. The most common ocular abnormality of probands was cataracts, and there were a few cases with posterior polymorphous corneal dystrophy and retinopathy with severe symptoms. The incidence of ocular abnormalities was 41%; among which, about 50% of male probands and 18% of female probands had ocular abnormalities. The median age of male probands with ocular abnormalities was 6 years old, while female probands had no record of onset age. Of these probands, 34% have advanced to Stage III or above of chronic kidney disease. All of them were male. The median age of occurrence was 25 years old. DL is not specifically associated with the progression of the disease. AS-DL probands have different clinical phenotypes. The most common clinical manifestations were hematuria, proteinuria, and diffuse leiomyoma, accounting for 17%. Second, hematuria and diffuse leiomyoma accounted for 14%. refer to the following figure for more information (Figures 6D–F).

In conclusion, the proportion of *de novo* mutations in AS-DL was significantly higher in female probands than in male probands (78 vs. 44%). The clinical manifestations of AS-DL in female probands were relatively few and mild. The incidence of proteinuria and ocular abnormalities was much lower than in male probands, and there was generally no hearing loss or progression to CKD Stage 3.

Probands with AS-DL were found to carry the contiguous deletion of the 5′ exons of *COL4A5* and *COL4A6*, with the break point located within the intron 3 of *COL4A6*. All of them include the 4.2 kb critical region, containing exon 1 of *COL4A5*, exons 1', 1, and 2 of *COL4A6*, and the common promoter region that regulates the expression of the two adjacent genes.

## Discussion

**Figure 7:**
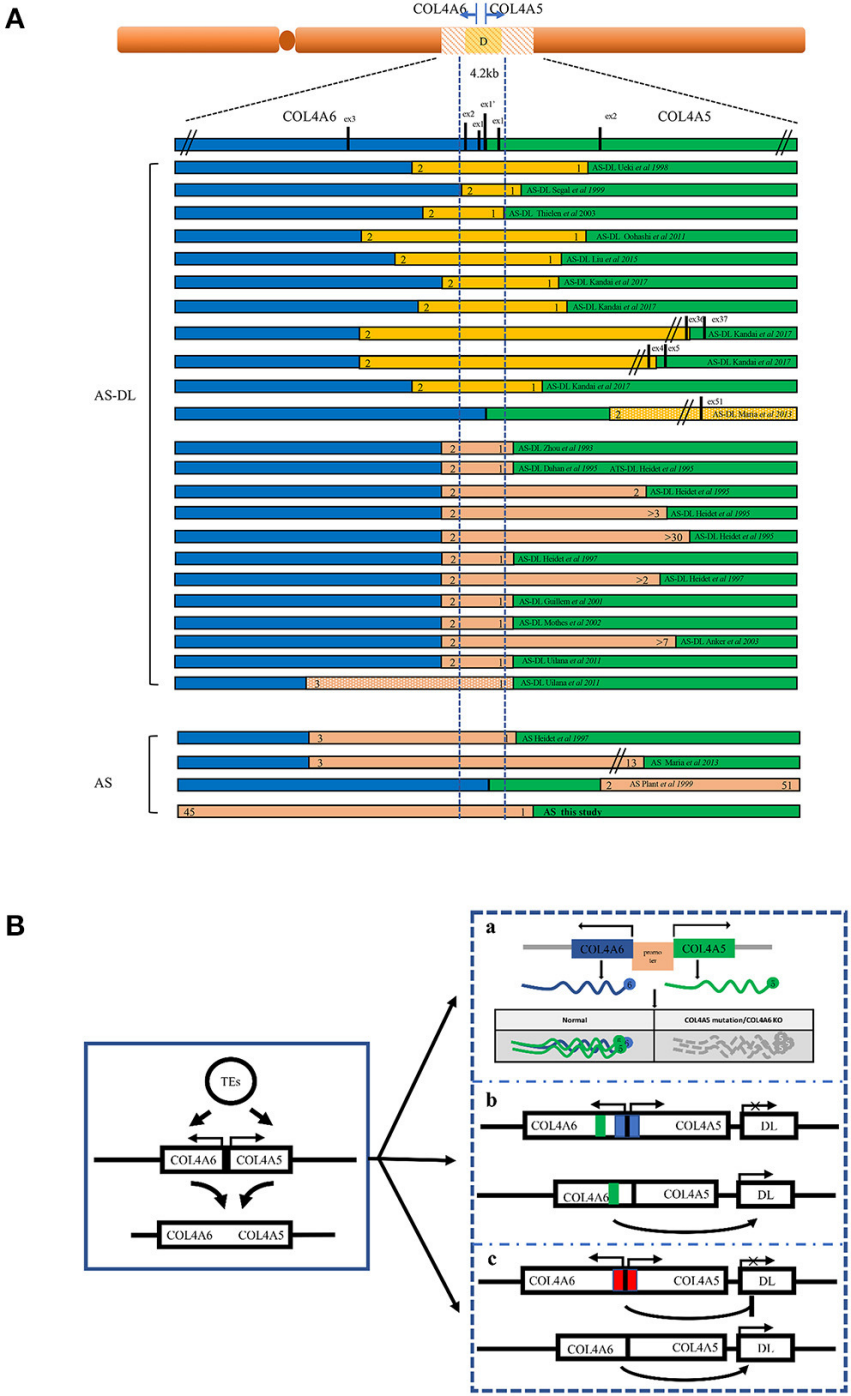
Schematic overview of the reported deletions within *COL4A5* and *COL4A6* and the models of the mechanism leading to AS-DL. **(A)** A summary of reported AS or AS-DL cases caused by contiguous deletion of *COL4A5* and *COL4A6* genes. **(B)** A schematic diagram of the mechanism of AS with DL caused by contiguous deletion of *COL4A5* and *COL4A6* genes. a: Absence of α5 (IV) and α6 (IV) collagen chain. The absence of the α5 (IV) collagen chain may change the structure of the ECM, which binds with the α5 (IV) collagen chain. The deregulation of cellular signals secreted from ECM may favour the proliferation of smooth muscle cells, leading to the development of DL. b: The silencer model in AS-DL. Deletion of the silencer element activates the gene involved in DL. c: The insulator model in AS-DL. Deletion of the insulator element activates the distal enhancers and/or neighbouring genes involved in DL.

All of the AS-DL families have been found to have a contiguous deletion of 5' exons of *COL4A5* and *COL4A6*. All of them contain 4.2 kb critical regions starting from intron 2 of *COL4A6* and ending at intron 1 of *COL4A5*. At present, all reported cases show that the deletion of exon 1 and exon 2 of the *COL4A6* gene will be affected by DL (10–26) (Figure 7A). When the deletion break point of the *COL4A6* gene extends to exon 3, most cases will not be affected by DL. However, in 2011, a case of AS-DL detected with deletion of the *COL4A6* gene extended into intron 3 was reported (19). No AS-DL case was reported with the contiguous deletion of *COL4A5*-*COL4A6* extended to the exon 4 of *COL4A6* or the whole *COL4A6* gene. Therefore, the break point of the deletion that can cause DL is located in intron 3 of the *COL4A6* gene. Contiguous *COL4A5* and *COL4A6* deletions extending upstream beyond exon 4 of *COL4A6*, or encompassing the entire *COL4A5* and *COL4A6* genes, or deletion mutations involving only *COL4A5*, were identified in patients with X-linked AS, who did not develop DL. In addition, mutation only in *COL4A6* has not been found in patients with AS-DL. However, the report in 2013 mentioned that a *COL4A5* deletion spanning exons 2 through 51, extending distally beyond *COL4A5* but proximally not into *COL4A6*, was detected in an AS-DL family, which segregated with the disease phenotype. The report mentioned that deletion of the 5′ exons of *COL4A6* is not needed to develop diffuse leiomyomatosis in patients with Alport syndrome, contrary to the recognised gene deletion characteristics of patients with AS-DL. However, the author did not detect the α6 (IV) collagen chain. It is impossible to determine whether the *COL4A6* gene is typically expressed (10).

The mechanism of leiomyoma caused by *COL4A6* deletion is not completely clear (Figure 7B). A hypothesis is that the partially deleted *COL4A6* gene expresses a truncated α6 (IV) chain, which leads to leiomyoma. However, the α6 (IV) collagen chain was not detected in the basement membrane of oesophageal leiomyoma resected from patients with AS-DL (27). The truncated α6 (IV) chain was absent in leiomyoma, so the truncated α6 (IV) chain may not be related to leiomyoma formation. In the X-AS canine model, the *COL4A5* gene carries unintentional point mutation, and the α6 (IV) chain cannot be assembled with an abnormal α5 (IV) chain to form a heterotrimerand express in the smooth muscle basement membrane. This study found that there was no leiomyoma in dogs lacking the α6 (IV) chain, which shows that the absence of the α6 (IV) chain or the absence of the α6 (IV) chain combined α5 (IV) chain cannot be the primary cause of DL. Therefore, there may be other mechanisms leading to DL (28). Deletion of a 4.2 kb minimal overlap region extending from intron 2 of *COL4A6* and intron 1 of *COL4A5* was observed in all patients with AS-DL. Another hypothesis is that a silencing element in this critical region may include a kind of microRNA and inhibit the expression of genes related to DL. Loss of this critical region contributes to the overgrowth of smooth muscle cells. However, patients with complete *COL4A6* deletion do not show DL, which contradicts this hypothesis. Alternatively, the 4.2-kb region may act as an insulator that protects against smooth muscle overgrowth afforded by inhibiting the distal enhancers and/or neighbouring genes. This deletion may activate a specific enhancer, which leads to smooth muscle cell overgrowth. The enhancer may locate in the undeleted part of intron 3 of *COL4A6*, which is consistent with the phenomenon that patients only show AS without DL when there is deletion extended to exon 4 of *COL4A6*. In addition, IRS4, a neighbouring gene of *COL4A5*, is also considered a candidate gene that may be activated (13). *IRS4* is connected with various growth factor receptors with tyrosine kinase activity (such as insulin receptor, IGF1R, and FGFR1) and a complex intracellular signal molecular network containing the SH2 domain. This molecular network plays an essential role in cell growth and proliferation. In addition, the α5 (IV) collagen chain has many binding sites of extracellular matrix (ECM) components; it also interacts with various integrin and non-integrin cell receptors. It is also possible that the absence of α5 (IV) chain or existence of abnormal α5 (IV) chain in the basement membrane leads to changes in the structure or function of other ECM components and/or cell surface receptors, thus leading to the overgrowth of smooth muscle cells (10, 29). However, these assumptions are not entirely satisfactory. The molecular mechanism of AS associated with DL remains to be clarified.

Another interesting question is, why are gene deletions in patients with XLAS concentrated at the junction of *COL4A5* and *COL4A6*? Other studies suggest that transposed elements (TEs) may play an essential role in the contiguous deletion of *COL4A5* and *COL4A6*. Transposed elements can provide new exons, genes, and regulatory sequences that greatly influence exon-intron structure formation and regulation of gene expression. Studies have found that the number of break points in TEs appears to be higher than expected of the general number of TEs of the genomic sequences of *COL4A6* and *COL4A5*. Therefore, TEs may promote the occurrence of non-allelic homologous recombination events, including AS-DL (12, 16, 30).

To sum up, this study reports an XLAS proband with deletion of the complete *COL4A6* gene and exon 1 of *COL4A5*. This case expanded the knowledge of genotype-phenotype correlations of AS. It is worth noting that according to the genotype-phenotype correlations in AS-DL, any woman with manifestations suggestive of DL but does not have any clinical or laboratory evidence of kidney disease should be considered for AS-DL diagnosis as male offspring are at risk for AS-DL. Since the pathogenesis of AS-DL is still unclear, further studies are needed to explore the pathogenesis.

## Data Availability Statement

The original contributions presented in the study are publicly available. This data can be found at: https://www.ncbi.nlm.nih.gov/sra/, PRJNA759552.

## Ethics Statement

The studies involving human participants were reviewed and approved by the Children's Hospital, Zhejiang University School of Medicine Ethics Committee. Written informed consent to participate in this study was provided by the participants' legal guardian/next of kin. Written informed consent was obtained from the individual(s), and minor(s)' legal guardian/next of kin, for the publication of any potentially identifiable images or data included in this article.

## Author Contributions

XZ drafted the initial manuscript and contributed to manuscript editing. JW collected the data from patients and contributed to manuscript editing. QY and JM devised the conceptual ideas, contributed to the discussion and interpretation of the results, and reviewed the final manuscript. All authors approved the final manuscript.

## Funding

This study was supported by the Key Project of Provincial Ministry Co-construction, Health Science, and Technology Project Plan of Zhejiang Province (WKJ-ZJ-2128), Key Laboratory of Women's Reproductive Health Research of Zhejiang Province (No. ZDFY2020-RH-0006), the National Natural Science Foundation of China (Grant/Award No: U20A20351), and Key Research and Development Plan of Zhejiang Province (Grant/Award No: 2021C03079).

## Conflict of Interest

The authors declare that the research was conducted in the absence of any commercial or financial relationships that could be construed as a potential conflict of interest.

## Publisher's Note

All claims expressed in this article are solely those of the authors and do not necessarily represent those of their affiliated organizations, or those of the publisher, the editors and the reviewers. Any product that may be evaluated in this article, or claim that may be made by its manufacturer, is not guaranteed or endorsed by the publisher.
