# Supplementary material for: Clinical Manifestations of Alport Syndrome-Diffuse Leiomyomatosis Patients With Contiguous Gene Deletions in COL4A6 and COL4A5
Source: Front Med (Lausanne). 2021 Oct 27;8:766224. doi: 10.3389/fmed.2021.766224 (PMC8578185; doi:10.3389/fmed.2021.766224)
Supplement: Supplementary file 4 [file Table_4.DOCX]

| **Supplementary table 1. Primers for PCR detection of *COL4A5* and *COL4A6* gene exons** | | | | | |
| --- | --- | --- | --- | --- | --- |
| **Genes** | **Exon** | **F terminal sequence** | **R terminal sequence** | **Fragment length（bp）** | **The annealing temperature（℃）** |
| *COL4A5* | exon1 | GGAGAAGAATGAAACTGCGTGG | GGAGGAAGGACTTACCGCAG | 104 | 60 |
|  | exon2 | TTCAGTTGAGCTGTAAGTCAGAGTC | CACACTTTGATCCTGGAGAACACC | 111 | 60 |
| *COL4A6* | exon1-exon2 | CAGACTAGTTGACTGAGCACCA | ATTTTCCCAACCCGTTACGAAG | 1986 | 60 |
|  | exon1-exon2 | TAAGGAAACAGCCTCCAAGCATC | TTTCCATAGCATCTGCGGCACT | 643 | 60 |
|  | exon3 | CAAAGTTCCCAAACCGGCTA | ACAATCTGGCTCTCTACAACC | 590 | 60 |
|  | exon4 | TGCCTCACAGATCTCTTAGCC | GCACCAAACTACTCAGGAATGACA | 376 | 60 |
|  | exon5 | ATTGGCTTGTTTGAATAGTCCAGT | CTCCGCTCTCCAATCATCCG | 714 | 60 |
|  | exon6 | ACAAGGAGCCACAGTTTAATAGCAT | GCCAGTATTGACTCCTACATTGGAC | 174 | 60 |
|  | exon7 | TCAAGGAATGTGTTTTGCCCCTTA | TTCTTTTGAATCCAGCTTGGTCTG | 368 | 60 |
|  | exon8 | GTAGGAAAATCACTAGCCCACA | TAACTCACAGCCTTACATGGAC | 796 | 60 |
|  | exon9-exon11 | AACATTCTTGTACAGTTCTCGTGGA | CATGTAGCCCTCAAGAGATCCC | 1402 | 60 |
|  | exon12 | GGGTGAAGAGGCCAAAGTAAGAG | TCATAAGGGGCAAAACAAAGAAGG | 383 | 60 |
|  | exon13 | ATCCTTTCTGTCTCCCAATTAGCC | ATGCTTACGTCTCCATGTGTTTGA | 509 | 60 |
|  | exon14 | TACATGGAACAGTGTTGTGGGTAG | ATTGGGTTGAGGAGAAACGAGAAG | 436 | 60 |
|  | exon15-exon16 | GTCTAGGAACATTCGCTTACCTGA | TTATATGCTCCAGGTGTAGGCAAA | 1078 | 60 |
|  | exon17-exon18 | CTCTTTGGGGAGTATTAGAATTCCT | CTACCTCTTGTTTGACTTTGGCTA | 1776 | 60 |
|  | exon19-exon20 | GATTTTCCTCTCAGGTTTGCCACT | GAGGCTGTTGGTTTTCTCATTCCC | 1473 | 60 |
|  | exon21-exon22 | TCTGCAAGCCTTCAGATTCCCTC | AGAAATGAGCAGGATAATAACAACCC | 1278 | 60 |
|  | exon23 | TGTGTTGTTAGATCACCCAGAGTAG | TTCTTCACGAAGTAGGGTGGAAAT | 611 | 60 |
|  | exon24-exon25 | CTAGCACTTGGACACTAGGAGCAT | CCTGTAATTGGCAGAGGACTACCAC | 777 | 60 |
|  | exon26-exon27 | TGCCTCCCAGAACTAGAACCTCC | CTCAAGCTTCTCCCCAACCAAA | 1354 | 60 |
|  | exon28 | ATAATCAGAAGGGGTTTGCTCTCC | GCACTAGCCTTGTCCAACTATCTT | 588 | 60 |
|  | exon29-exon30 | TTTGTATTGTGTAGTGCCATGTCC | TAACAAATGGCAGTCTCAAAGTCC | 1134 | 60 |
|  | exon31 | CAGTGGTATAGCAGAAGCAACTCT | TGTGAACAAAAGAACAGAGGAGGA | 574 | 60 |
|  | exon32-exon33 | CTTGCCACAGAATACATGATGGAC | TGATTAGGCCATATTTCTAGCAGT | 1710 | 60 |
|  | exon34-exon36 | AGACTCATAGACTGGCAAAGACCT | GCCTTCCTGCCTAACTCACTCG | 1560 | 60 |
|  | exon37 | TGAGGTCCCTTGATATGGAGAAGA | GGAGGAACAGAAGAGAATCTGGAG | 568 | 60 |
|  | exon38-exon40 | TGGTCTGGCTCCTACCTAACTGCT | CATCCAGACTTTCTTGGCGTGGTG | 1113 | 60 |
|  | exon41 | TCCTGTGGGGACAAACAGGATA | AAAGCCAAGTACCAAGTACAACCA | 466 | 60 |
|  | exon42-exon43 | TCTTGGGCATGACACATAAGTCC | CATTAAGGAGGAAGCCAAAGCCTA | 1592 | 60 |
|  | exon44 | ACTTCAGCACATGTACAATGTTACAGG | GGTTCTCCATTCTTTAGCCAAGCC | 580 | 60 |
|  | exon45 | TGAGACAAGGAACCAGGGCTTA | CTTGACAGAAACAGGACAGCAGAT | 605 | 60 |
| Reference genes (CYBB） |  | AGTCACTCTGCTCCCTTTCC | CGACAGACTGGCAAGAGAATC | 109 | 60 |
| **Note: Reference gene and variant *COL4A6* gene: NM_033641.3. Repeated detection of Exon1 and Exon2 was performed.** | | | | | |
|  |  |  |  |  |  |
|  |  |  |  |  |  |
