# Supplementary material for: Clinical Manifestations of Alport Syndrome-Diffuse Leiomyomatosis Patients With Contiguous Gene Deletions in COL4A6 and COL4A5
Source: Front Med (Lausanne). 2021 Oct 27;8:766224. doi: 10.3389/fmed.2021.766224 (PMC8578185; doi:10.3389/fmed.2021.766224)
Supplement: Supplementary file 5 [file Data_Sheet_1.PDF]

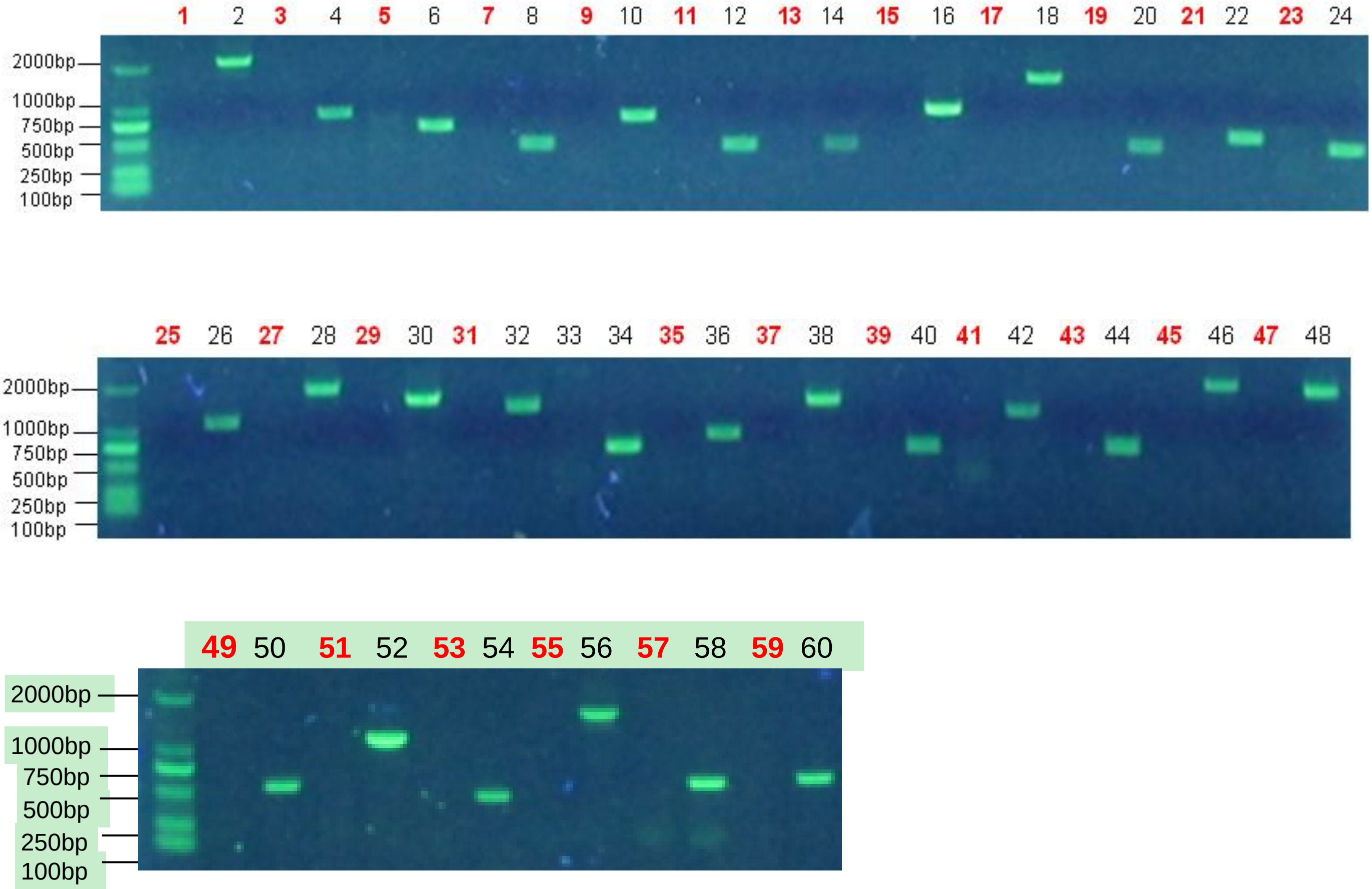

1 2 3 4 5 6 7 8 9 10 11 12 13 14 15 16 17 18 19 20 21 22 23 24
2000bp
1000bp
750bp
500bp
250bp
100bp
25 26 27 28 29 30 31 32 33 34 35 36 37 38 39 40 41 42 43 44 45 46 47 48
2000bp
1000bp
750bp
500bp
250bp
100bp
49 50 51 52 53 54 55 56 57 58 59 60
2000bp
1000bp
750bp
500bp
250bp
100bp

上图为针对COL4A6基因的正常对照样本与先证者样本同组进行PCR的电泳结果，对各外显子进行扩增，片段设计大小为1986bp、643bp、590bp、376bp、714bp、174bp、368bp、796bp、1402bp、383bp、509bp、436bp、1078bp、1776bp、1473bp、1278bp、611bp、777bp、1354bp、588bp、1134bp、574bp、1710bp、1560bp、568bp、1113bp、466bp、1592bp、580bp、605bp，其中2、4、6、8、10、12、14、16、18、20、22、24、26、28、30、32、34、36、38、40、42、44、46、48、50、52、54、56、58、60泳道对应正常对照样本，有明显特异条带，且产物长度均与设计长度吻合；1、3、5、7、9、11、13、15、17、19、21、23、25、27、29、31、33、35、37、39、41、43、45、47、49、51、53、55、57、59泳道对应先证者样本，无特异扩增条带；先证者和正常对照样本的对照基因扩增结果，均有明显特异条带，且产物长度与设计长度吻合（图中未列）。提示先证者COL4A6基因可能为纯合缺失。

本项目是针对特定对象提供的样品进行分子生物学分析研究的科研技术服务,非医保临检项目。本结果仅对该样品负责。本报告中“突变影响”分析无法确保查遍所有文献及跟踪最新文献，且文献观点仅供参考，不代表本实验室观点。实验室不参与、亦不负责对该结果的进一步分析及应用。

检测员：　　　　审核员：
