# Supplementary material for: Clinical Manifestations of Alport Syndrome-Diffuse Leiomyomatosis Patients With Contiguous Gene Deletions in COL4A6 and COL4A5
Source: Front Med (Lausanne). 2021 Oct 27;8:766224. doi: 10.3389/fmed.2021.766224 (PMC8578185; doi:10.3389/fmed.2021.766224)
Supplement: Supplementary file 6 [file Data_Sheet_2.PDF]

北京德易东方转化医学研究中心

基因分析报告（科研）

<table>
<tr><td>受检者</td><td>裘景文</td><td>男</td><td>12岁9月</td><td>病案号</td><td></td><td>送检时间</td><td>2015年10月9日</td><td rowspan="2">研究项目</td><td rowspan="2">人类四千种遗传病基因突变筛查+CNV分析</td></tr>
<tr><td>编号</td><td>1018429NT01</td><td>样本类型</td><td colspan="3">EDTA抗凝血</td><td>报告时间</td><td>2015年12月7日</td></tr>
<tr><td colspan="2">临床怀疑疾病</td><td colspan="8"></td></tr>
<tr><td colspan="2">临床特征</td><td colspan="8">怀疑Alport综合征。</td></tr>
</table>

本项目是针对特定对象提供的指定样品进行分子生物学分析研究的科研技术服务，非医保临检项目。本结果仅对该样品负责。本报告中所列示结果均为实验室检测数据，仅用于本项目所涉及的单基因遗传病相关基因近全部编码区序列的单碱基转换、颠换和小范围（100bp以内）插入缺失的突变筛查，突变筛查不包含100bp以上片段的缺失重复、poly结构和串联重复序列等不适合测序技术检测的序列，以及存在同源相似序列（假基因）的序列检测，检测结果不代表最终诊断结果。本报告为对本次检测得到的大量突变筛选的初步结果，并非最终检测结果，最终阳性检测结果为经一代测序技术验证后的结果。本报告中所列示突变未必都会导致受检者发病，是否致病需结合突变影响、遗传方式及临床特征等多因素综合分析。本报告中与疾病相关性、相关性说明、突变关联疾病及疾病临床特征等信息均来自现有已报道的研究结果，均基于我们对医学遗传学的目前的认识水平，无法确保查遍所有文献及跟踪最新文献，仅供参考。受检者临床特征及临床怀疑疾病均来自医生或患者提供，实验室无法对该信息及基于该信息所做出的分析结果的真实性、准确性、全面性做出保证。报告中临床特征匹配分析仅供医生参考，不能代表医生诊断意见。实验室不参与、亦不负责对该结果的进一步分析及应用。本实验室对下列检测结果保留最终解释权。

检测员：　　　　　　审核员：

一、结论：

(1)发现以下疾病相关性突变（见主要检测结果）。
(2)拷贝数变异（Copy number variations，CNV）分析未见异常。

二、主要检测结果：

基因变异位点：

突变信息										基因功能或关联疾病表型
基因	染色体位置	核酸改变	氨基酸改变	rs编号	MAF	杂合/纯合	与疾病相关性	相关性说明	PubMed文献号	
COL4A3	chr2:228163437	c.3791T>C (E43)	p.1264, I>T			杂合	可能相关	蛋白结构预测结果为有害		常染色体显性遗传的Alport综合征,AD

COL4A6	chrX:107681416-107681658；<br>chrX:107681172-107681223；<br>chrX:107553978-107554058；<br>chrX:107464470-107464604；<br>chrX:107462928-107462972；<br>chrX:107457342-107457458；<br>chrX:107454902-107454970；<br>chrX:107453199-107453234；<br>chrX:107449748-107449810；<br>chrX:107448890-107448925；<br>chrX:107448669-107448710；<br>chrX:107447550-107447642；<br>chrX:107446158-107446211；<br>chrX:107439741-107439809；<br>chrX:107438312-107438356；<br>chrX:107437764-107437817；<br>chrX:107436858-107436927；<br>chrX:107435703-107435810；<br>chrX:107434623-107434763；<br>chrX:107433622-107433726；<br>chrX:107431747-107431907；<br>chrX:107431078-107431257；<br>chrX:107430326-107430509；<br>chrX:107424127-107424198；<br>chrX:107423745-107423852；<br>chrX:107422447-107422668；<br>chrX:107421914-107422075；<br>chrX:107420071-107420241；<br>chrX:107418884-107419027；<br>chrX:107418320-107418445；<br>chrX:107417670-107417851；<br>chrX:107415699-107415762；<br>chrX:107414617-107414691；<br>chrX:107414040-107414147；<br>chrX:107413839-107413946；	全部外显子缺失	缺失			半合子	较高	整个基因外显子缺失		弥漫型平滑肌瘤病伴Alport综合征

COL4A6	chrX:107413175-107413246；<br>chrX:107412725-107412850；<br>chrX:107408600-107408716；<br>chrX:107408107-107408268；<br>chrX:107407830-107407928；<br>chrX:107406122-107406268；<br>chrX:107404849-107404965；<br>chrX:107403693-107403884；<br>chrX:107402692-107402978；<br>chrX:107398837-107400490；	全部外显子缺失	缺失			半合子	较高	整个基因外显子缺失		弥漫型平滑肌瘤病伴Alport综合征

三、主要检测结果列表项注释：

一）**基因**：即发现突变的基因名称，这里原则上列出的均为NCBI上的官方命名（Official Name）。
二）**染色体位置**：指突变在染色体上的绝对坐标位置，这个位置是唯一的（参考基因组Hg19）。
三）**核酸改变**：指检测到的DNA上的碱基排序变异。
1. 点突变（碱基颠换）的核酸改变表示："c.（或m.）**（数字）碱基>碱基（E**)"，或IVS*+/-**（数字）碱基>碱基"，其中c.表示该基因编码区，m.表示该基因非编码区的UTR位置，如：**1)** c.8306C>T，即表示：该基因的编码区8306位的碱基C突变为T；**2)** m. 7236G>T，即表示：该基因的UTR区7236位的碱基G突变为T；**3)** IVS4-1C>A，即表示：该基因的内含子4的负1位的碱基C突变为A。
2. 插入缺失突变位置的核酸改变表示："c.（或m.或IVS*+/-）**（起始位置数字）"至"**（起始位置数字）：插入/缺失&碱基"。如IVS4+14至IVS4+20:插入T，即表示：从内含子4的14位置到20位置，插入碱基T，并且意味着该起止位置的7个碱基原本都是T，突变后增加了一个T；最后的"（E**）"表示突变位于第几外显子，比如(E15)，则表示突变位于第15外显子。
四）**氨基酸改变**：DNA的突变对氨基酸序列带来的变化。氨基酸是蛋白质的基本组成单位，编码氨基酸的基因序列发生改变，就可能造成蛋白序列的变化。报告中常见的氨基酸改变主要有以下几种：**1.** 错义突变：DNA的变异导致该位置氨基酸的改变，这类变异叫做错义突变。对于错义，表示为："p.**（数字）氨基酸>氨基酸"，如p.856Y>H，即表示：该基因编码的蛋白的第856氨基酸蛋白位置上，氨基酸Y突变为氨基酸H；**2.** 无义突变：表示为："p.**（数字1）氨基酸>氨基酸（（数字2））"，这种突变导致数字1位置的氨基酸变为终止密码子（用X表示），从而导致蛋白的氨基酸序列在此处异常终止；数字2表示该终止突变引起蛋白缩短的长度；如"p.364E>X(216)",即表示该基因的该无义突变导致其编码的蛋白第364位氨基酸之后的216个氨基酸均无法翻译，从而导致蛋白缩短了216个氨基酸。**3.** 剪接位点突变：这种突变会影响DNA的转录过程，导致转录产物mRNA序列的异常，从而导致蛋白结构的异常。**4.** 框移突变：也叫做移码突变，是指插入或缺失N个碱基的突变，N为非3的整数倍的数字，这种突变会导致读码框发生错位，从而导致该位置及之后的蛋白的氨基酸序列的明显改变。表示为："p.**（数字1）氨基酸>氨基酸fs（（数字2））"，fs表示frameshift（移码）。数字2表示从突变位置开始算起，后面还可以翻译但框移的氨基酸的数量。如"p.80,N>Mfs6"，即表示该基因的该框移突变导致其编码的蛋白从第80位氨基酸开始算起，后面再翻译6个氨基酸即终止，且这6个氨基酸是移码的，序列和原来的序列不同。**5.** 对于造成蛋白序列长度变化的，如插入缺失突变、框移突变（造成移码的插入缺失）或剪切位点突变等，则不予列示氨基酸的具体变化，列表中标记为"插入（/缺失）移码（/非移码）"、"剪切位点"；**6.** 对于非编码区如UTR或不影响剪接的内含子突变，该栏目标记为"非编码区"；
五）**rs编号**：该变异在dbSNP数据库中的编号。**一个突变有无rs号与该突变是否致病没有直接关系。如果无rs编号提示可能是新发现的突变位点。**
六）**MAF**：即次要等位基因频率（Minor Allele Frequency）
1. 在本报告中，MAF可以通俗理解为该变异的杂合形式在人群中的分布率（出现概率）。如MAF为0.0215，则表示该变异的杂合形式在人群中的分布率为2.15%。
2. MAF数据可以用于区别普通变异和罕见变异。MAF越小就说明群体中突变概率越低，说明是罕见突变；一般以1%作为与疾病相关性的判断的边界线，即MAF>1%时，一般认为不属于罕见疾病的致病突变。但这也不是绝对的，一方面MAF值是在不断修正的，随着人类基因组数据库的不断扩充，很多变异的MAF都在发生变化；另一方面，还要结合具体情况分析，比如对于MAF为2%的纯合变异，该纯合型在人群中出现的概率为2%×2%=0.04%，那就比杂合型罕见多了，就不能简单归为无意义的SNP了。
七）**纯合/杂合**：杂合是指在由父母遗传来的一对等位基因中，其中一个基因上发现了突变，而另一个基因序列是正常的。而纯合是指两个等位基因的相同位置上都发现了突变，没有任何一个基因是正常的。对于常染色体上的突变，纯合突变是一对等位基因都存在突变，而杂合突变指等位基因中只有其中一个基因出现突变；对于男性的X染色体上的突变，因为只有一个等位基因，因此只有纯合类型。
八）**与疾病相关性**：指该突变与疾病的相关性，分为较高、可能相关和未知三类。"与疾病相关性较高"的情况（强烈提示阳性的基因突变）：有致病相关性文献、中等缺失或外显子缺失、无义（截短）、RunOn（加长）突变、框移突变、剪切位点突变；"可能相关"的情况（提示阳性可能的基因突变）：蛋白结构预测有害；"未知"的情况（不排除致病可能性）：蛋白结构预测为容忍或无害。
九）**相关性说明**：指对左边（上述八）"与疾病相关性"结论的说明。
十）**PubMed文献号**：该突变与疾病的相关性如在OMIM数据库中有文献报道，则提供PubMed文献号，根据文献号可以在PubMed数据库中查找相应的文献，需注意的是，文献中所提及的疾病与报告中的基因关联疾病有可能不尽相同，且OMIM数据库收录文献未必全面及时。
十一）**基因功能或关联疾病表型**：本报告中对于与疾病相关性已经明确的基因，则列出该基因关联的疾病；对于与疾病相关性尚不明确的基因，则给出该基因的基本功能。
十二）**遗传方式**：指该疾病所呈现的孟德尔遗传方式，分为AD（常染色体显性遗传）、AR（常染色体隐性遗传）、XD（X染色体显性遗传）、XR（X染色体隐性遗传），共显性遗传、Y染色体遗传等。一种疾病有可能有多种遗传方式。大部分常染色体显性遗传病为不完全显性。

四、附：上述变异基因相关疾病临床特征（参考）：

根据医师送检时提供的患者病历信息：怀疑Alport综合征。在基因所对应疾病中匹配较高的是：

1 常染色体显性遗传的Alport综合征：常染色体显性遗传ALPORT综合征;常染色体显性遗传;尤其影响高频率的感音神经性耳聋;肾小球性肾病;肉眼血尿和镜下血尿;蛋白尿;终末期肾功能衰竭;肾小球基底膜变薄;(疾病早期);肾小球基底膜增厚;(疾病晚期);肾小球基底膜分裂;肾小球基底膜弥漫性纹理;高血压;肉眼血尿和镜下血尿;蛋白尿;渐进性疾病;听力丧失是可变的;由胶原蛋白;IV型;α3基因突变引起的(COL4A3;120070.0009);

2 弥漫型平滑肌瘤病伴Alport综合征：扩散性平滑肌瘤病伴遗传性肾炎综合征;X连锁遗传;发育停滞;白内障;前圆锥形晶状体;尤其影响高频率的感音神经性耳聋;气管支气管平滑肌瘤病;支气管刺激;呼吸困难;食管平滑肌瘤病;吞咽困难;便秘;生殖器平滑肌瘤病;血尿;肉眼和显微镜;蛋白尿;肾小球肾病;终末期肾衰竭;肾小球基底膜薄（发病早期）;肾小球基底膜厚（疾病晚期）;肾小球基底膜分裂;肾小球基底膜弥漫性薄层状;血尿;肉眼和显微镜;蛋白尿;儿童期发病;在第二个或第三个十年肾衰竭;由胶原蛋白IV型α-5;(COL4A5;303630)以及胶原蛋白IV型α-6(COL4A5;303630)基因缺失引起

五、附：本次检测发现的其他临床意义未明的SNP：

基因突变及良性多态性变异:

基因	染色体位置	核酸改变	氨基酸改变	rs编号	MAF	杂合/纯合	与疾病相关性	相关性说明	PubMed文献号	基因功能或关联疾病表型
COL4A3	chr2:228163437	c.3791T>C(E43)	p.1264,I>T		0.0012(DYDF数据库)	杂合	可能相关	蛋白结构预测结果为有害		良性家族性血尿,AD;常染色体隐性遗传性遗传性肾炎（Alport综合征）,AR

良性家族性血尿：常染色体显性遗传;镜下血尿;肾小球基底膜薄;通常在童年发病;非渐进性疾病;不会导致肾功能衰竭;由胶原蛋白IV型;α-3基因突变引起（COL4A3;120070.0007）;由胶原蛋白IV型;α-4基因突变引起（COL4A4;120131.0003）;

常染色体隐性遗传性遗传性肾炎（Alport综合征）：常染色体隐性遗传;常染色体隐性遗传;前圆锥形晶状体;晶状体混浊;白内障;近视;黄斑周围色素变化（“斑点”）;角膜内皮细胞囊泡;角膜糜烂;感音神经性耳聋;尤其影响高频率;肾小球性肾病;终末期肾功能衰竭;肾小球基底膜变薄（在疾病早期）;肾小球基底膜增厚（在疾病后期）;肾小球基底膜分裂;肾小球基底膜弥漫性薄层状;高血压;镜下血尿和肉眼血尿;蛋白尿;肾病综合征;儿童期发病;渐进性疾病;听力损失发生在童年后期;听力损失和眼部发现是多变的;约1至5%接受了肾移植的患者可抵抗肾小球基底膜肾炎;遗传异质性;由胶原蛋白;IV型;α-3基因突变引起（COL4A3;120070.0001）;由胶原蛋白;IV型;α-4基因突变引起（COL4A4;120131.0001）;

基因突变及良性多态性变异：

基因	染色体位置	核酸改变	氨基酸改变	rs编号	MAF	杂合/纯合	与疾病相关性	相关性说明	PubMed文献号	基因功能或关联疾病表型
CFH	chr1:196706049	c.2509G>A(E16)	p.837,V>I	rs55807605	0.0034	杂合	可能相关	蛋白结构预测结果为有害		补体因子H缺乏症,AD,AR;非典型溶血性尿毒症综合征，易感1型,AD,AR
**补体因子H缺乏症：**常染色体显性遗传;常染色体隐性遗传;进行性肾功能衰竭;膜增生性肾炎II型;肾活检肾小球基底膜增厚;补体C3沉积在肾小球基底膜;血尿;持续激活的补体替代途径;低补体血症;补体替代途径的组成部分缺失;某些细菌感染;尤其是脑膜炎奈瑟菌易感性增加;血清补体因子H降低;补体因子H;水平正常;但功能受损;低补体血症;婴儿期或儿童期发病;变量表型;有些病人可无症状;由补体因子H基因的突变引起的（;CFH;134370.0002;）										
**非典型溶血性尿毒症综合征，易感1型：**常染色体显性遗传;常染色体隐性遗传;在幼儿中;前期症状为胃肠腹泻;通常因大肠杆菌（E.;coli;0157-H7）或志贺氏杆菌导致(典型溶血尿毒综合症;HUS);严重肾衰竭;无尿;高血压（尤其是非典型性溶血性尿毒综合征（aHUS）患者）;全身和局灶性神经功能异常（少于30%）;癫痫;昏迷;轻偏瘫;认知缺失;视觉缺失;言语障碍;微血管病性溶血性贫血;血小板减少;血栓性微血管病;网织红细胞增多;裂红细胞;毛刺细胞;补体消耗;补体调节缺陷;补体系统激活;部分病人可能有因子H的自身免疫抗体;导致因子H不足;发烧;血红蛋白减少;血尿氮上升（BUN）;肌酸酐上升;血清中因子H减少（非典型性溶血尿毒症综合征;HUS）;血清中因子I减少（非典型性溶血尿毒症综合征;HUS）;血清中C3减少（非典型性溶血尿毒症综合征;HUS）;血清中因子B减少（非典型性溶血尿毒症综合征;HUS）;高血脂;血管性血友病因子裂解酶活性正常;腹泻相关(D+溶血尿毒综合征;HUS);见于3岁以下儿童;与vero毒素产生性大肠杆菌相关（90%的病人）;D+HUS;（典型性溶血尿毒综合征;HUS）通常是零星发病;限于一例;预后较好;腹泻-负亚型(D-HUS)或非典型性溶血尿毒综合征（HUS）具有更严重的症状并且经常复发;D+HUS;通常为家族性的;表型与血栓性血小板减少性紫癜重叠(TTP;274150);可能由药物（包括抗肿瘤药物;免疫治疗药物和抗血小板药物）引发;敏感性与补体因子H基因突变有关;(CHF;134370.0001);敏感性与补体因子H相关蛋白1基因;(CFHR1;134371.0001)和补体因子H相关蛋白3基因;(CFHR3;605366.0001)突变有关;										
PDP1	chr8:94930144	c.2T>C(E2)	p.1,M>T			杂合	较高	起始密码子突变，影响蛋白编码起始位点		丙酮酸脱氢酶磷酸酶缺陷,AR
DSG2	chr18:29118743-29118743	c.1681(E12):缺失A	p.561,K>Kfs11			杂合	较高	理论上这种移码突变会导致蛋白序列明显改变		致心律失常性右室发育不良10,AD;扩张性心肌病1BB型
SEPT9	chr17:75303247-75303247	c.44(E2):缺失G	p.15,R>Rfs13			杂合	较高	理论上这种移码突变会导致蛋白序列明显改变		遗传性神经痛性肌萎缩症,AD

基因突变及良性多态性变异:

基因	染色体位置	核酸改变	氨基酸改变	rs编号	MAF	杂合/纯合	与疾病相关性	相关性说明	PubMed文献号	基因功能或关联疾病表型
RP1L1	chr8:10480295-10480296	c.416(E2)至c.417(E2):插入C	p.139,P>Pfs11	rs201192645		杂合	较高	理论上这种移码突变会导致蛋白序列明显改变		隐匿性黄斑营养不良,AD
OPA1	chr3:193360767	c.1069G>A(E11)	p.357,A>T	rs190223702	0.0004	杂合	较高	有疾病相关报道	19319978;	正常眼压性青光眼;视神经萎缩1型,伴耳聋、眼肌瘫痪、肌病、运动失调和神经疾病,AD;视神经萎缩1型,AD
HNF1A	chr12:121426790	c.481G>A(E2)	p.161,A>T	rs201095611		杂合	较高	有疾病相关报道	9754819;	年轻起病成人型糖尿病3型,AD;新生儿糖尿病（胰岛素抵抗型）,AR;非胰岛素依赖型糖尿病(易感),AD;胰岛素依赖型糖尿病20型
STRC	chr15:43893734	c.4561C>G(E24)	p.1521,R>G	rs138763871		杂合	可能相关	蛋白结构预测结果为有害		常染色体隐性遗传耳聋16
AGXT2L2	chr5:177657017	c.262A>G(E3)	p.88,N>D	rs146105181	0.0006	杂合	可能相关	蛋白结构预测结果为有害		磷酸羟基赖氨酸尿,AR
TAF4B	chr18:23865998	c.1125G>T(E7)	p.375,Q>H	rs148172329	0.0078	杂合	可能相关	蛋白结构预测结果为有害		精子生成障碍13型,AR
CD320	chr19:8369919-8369921	c.262(E2)至c.264(E2):缺失GAG	缺失非移码	rs150384171	0.007	杂合	可能相关	蛋白结构预测结果为有害		甲基丙二酸尿运钴胺素蛋白受体缺陷
SDHAF2	chr11:61205157	c.97C>T(E2)	p.33,R>C	rs144867876	0.0016	杂合	可能相关	蛋白结构预测结果为有害		副神经节瘤2型,AD
NPHP4	chr1:5923461	c.4145G>A(E30)	p.1382,G>E			杂合	可能相关	蛋白结构预测结果为有害		Senior-Loken综合征(肾视网膜营养不良)4型,AR;肾消耗病4型,AR
MAP2K2	chr19:4099225	c.893C>T(E7)	p.298,P>L	rs200371894	0.0024	杂合	可能相关	蛋白结构预测结果为有害		Cardiofaciocutaneous综合症4型,AD

基因突变及良性多态性变异:

基因	染色体位置	核酸改变	氨基酸改变	rs编号	MAF	杂合/纯合	与疾病相关性	相关性说明	PubMed文献号	基因功能或关联疾病表型
ATM	chr11:108170498	c.5063T>C(E34)	p.1688,I>T	rs199836342	0.0002	杂合	可能相关	蛋白结构预测结果为有害		共济失调性毛细血管扩张症,AR
APOL4	chr22:36597750	c.31G>A(E3)	p.11,V>I	rs376470983		杂合	可能相关	蛋白结构预测结果为有害		精神分裂症,AD
SYNJ1	chr21:34050984	c.1598G>A(E12)	p.533,R>Q			杂合	可能相关	蛋白结构预测结果为有害		帕金森氏病20型（早发性）,AR
LTC4S	chr5:179222839	c.211G>C(E3)	p.71,G>R			杂合	可能相关	蛋白结构预测结果为有害		白三烯C4合酶缺乏症,AR
EPG5	chr18:43488030	c.4222A>G(E24)	p.1408,I>V	rs369984684	0.0002	杂合	可能相关	蛋白结构预测结果为有害		Vici综合征,AR
KIAA1033	chr12:105536238	c.1795A>T(E19)	p.599,T>S	rs1345092	0.0082	杂合	可能相关	蛋白结构预测结果为有害		常染色体隐性遗传性智力发育迟滞43型,AR
FLNB	chr3:58109181	c.3488C>T(E21)	p.1163,P>L			杂合	可能相关	蛋白结构预测结果为有害		脊椎骨性结合综合征,AR;骨发育不全3型,AD;拉森氏综合征,AD;飞去来器样骨发育不良,AD;骨发育不全1型
C8B	chr1:57409446	c.1157G>T(E8)	p.386,G>V	rs373307501		杂合	可能相关	蛋白结构预测结果为有害		补体成分8缺乏症2型,AR
CARD11	chr7:2977555	c.1129C>T(E8)	p.377,R>W			杂合	可能相关	蛋白结构预测结果为有害		免疫缺陷11型,AR;持续性多克隆B淋巴细胞增多症,AD
USH1C	chr11:17552979	c.215T>A(E3)	p.72,V>E			杂合	可能相关	蛋白结构预测结果为有害		常染色体隐性遗传耳聋18型,AR;尤塞氏综合症IC型,AR
VCAN	chr5:82816838	c.2713A>G(E7)	p.905,K>E			杂合	可能相关	蛋白结构预测结果为有害		Wagner综合症,AD
ZNF469	chr16:88495349	c.1471G>A(E1)	p.491,A>T	rs117555121	0.01	杂合	可能相关	蛋白结构预测结果为有害		脆性角膜综合症1型,AR

基因突变及良性多态性变异:

基因	染色体位置	核酸改变	氨基酸改变	rs编号	MAF	杂合/纯合	与疾病相关性	相关性说明	PubMed文献号	基因功能或关联疾病表型
COL7A1	chr3:48630293	c.761C>T(E6)	p.254,A>V	rs201916805	0.0002	杂合	可能相关	蛋白结构预测结果为有害		新生儿暂时性大疱性皮肤松解症,AD,AR;胫前营养不良性大疱性表皮松解,AD;表皮松解症伴先天性皮肤局部缺失和指甲畸形,AD;先天性缺甲症8型,AD;痒疹样大疱性表皮松解症,AD,AR;常染色体隐性营养不良性大疱性表皮松解,AR;常染色体显性营养不良性大疱性表皮松解,AD
HPSE2	chr10:100503697	c.727A>T(E4)	p.243,S>C	rs188784527	0.0008	杂合	可能相关	蛋白结构预测结果为有害		urofacial综合征,AR
TBC1D7	chr6:13316916	c.406G>A(E5)	p.136,A>T	rs9381921	0.002	杂合	可能相关	蛋白结构预测结果为有害		巨脑症,AR
TMPRSS6	chr22:37480371	c.1187C>T(E10)	p.396,P>L			杂合	可能相关	蛋白结构预测结果为有害		铁剂难治性缺铁性贫血/小细胞低色素性贫血伴铁代谢障碍,AR
SPG11	chr15:44876248	c.5630C>T(E30)	p.1877,S>L			杂合	可能相关	蛋白结构预测结果为有害		遗传性痉挛性截瘫11型,AR
ASPM	chr1:197112538	c.844A>C(E3)	p.282,N>H	rs113777932	0.0034	杂合	可能相关	蛋白结构预测结果为有害		常染色体隐性原发性小头畸形5型,AR
	chr1:197112477	c.905G>A(E3)	p.302,C>Y	rs77736715	0.0034	杂合	可能相关	蛋白结构预测结果为有害		
	chr1:197112375	c.1007C>A(E3)	p.336,T>K	rs112113370	0.0034	杂合	可能相关	蛋白结构预测结果为有害		
NBAS	chr2:15613423	c.1648G>T(E16)	p.550,G>C	rs571873632	0.0004	杂合	可能相关	蛋白结构预测结果为有害		身材矮小,视神经萎缩和佩-休二氏异常,AR
ARID1A	chr1:27105700	c.5311C>T(E20)	p.1771,P>S	rs187631645	0.0004	杂合	可能相关	蛋白结构预测结果为有害		常染色体显性遗传性精神智力发育迟滞14型

基因突变及良性多态性变异:

基因	染色体位置	核酸改变	氨基酸改变	rs编号	MAF	杂合/纯合	与疾病相关性	相关性说明	PubMed文献号	基因功能或关联疾病表型
HLA-C	chr6:31238931	c.538C>G(E3)	p.180,L>V			杂合	可能相关	蛋白结构预测结果为有害		银屑病易感1型;人类免疫缺陷病毒易感1型
	chr6:31239114	c.355C>T(E3)	p.119,L>F			杂合	可能相关	蛋白结构预测结果为有害		
	chr6:31238930	c.539T>A(E3)	p.180,L>Q			杂合	可能相关	蛋白结构预测结果为有害		
RELN	chr7:103234329	c.3712A>C(E27)	p.1238,N>H	rs114003896	0.0024	杂合	可能相关	蛋白结构预测结果为有害		无脑回畸形2型,AR
BFSP1	chr20:17475218	c.1499C>T(E8)	p.500,A>V	rs550597037	0.0002	杂合	可能相关	蛋白结构预测结果为有害		青少年皮质性白内障
DDX59	chr1:200635655	c.214G>A(E2)	p.72,V>I			杂合	可能相关	蛋白结构预测结果为有害		口面指综合征5型,AR
CACNA1C	chr12:2676738	c.1673C>T(E13)	p.558,T>M	rs572234918	0.0006	杂合	可能相关	蛋白结构预测结果为有害		Brugada综合征3型;先天性长Q-T间期综合征8型/蒂莫西综合症,AD
ATP7B	chr13:52548790	c.566C>T(E2)	p.189,P>L			杂合	可能相关	蛋白结构预测结果为有害		肝豆状核变性,AR
ATL3	chr11:63400521	c.1084A>G(E11)	p.362,I>V	rs139006668	0.0012	杂合	可能相关	蛋白结构预测结果为有害		遗传性感觉自主神经病IF型,AD
PAX4	chr7:127253551	c.574C>A(E5)	p.192,R>S	rs3824004	0.0046	杂合	可能相关	蛋白结构预测结果为有害		糖尿病，酮症倾向;非胰岛素依赖型糖尿病(易感),AD;年轻起病成人型糖尿病9型
WISP3	chr6:112385978	c.421G>A(E3)	p.141,E>K			杂合	可能相关	蛋白结构预测结果为有害		儿童期进行性假风湿关节病/晚发型脊柱骨骺发育不良伴进行性骨关节病,AR
CSF2RB	chr22:37322141	c.313G>A(E4)	p.105,V>I	rs373460188	0.0004	杂合	可能相关	蛋白结构预测结果为有害		肺泡蛋白沉着症5型,AR

基因突变及良性多态性变异:

基因	染色体位置	核酸改变	氨基酸改变	rs编号	MAF	杂合/纯合	与疾病相关性	相关性说明	PubMed文献号	基因功能或关联疾病表型
TREH	chr11:118532376	c.587T>C(E6)	p.196,M>T	rs147473566	0.0098	杂合	可能相关	蛋白结构预测结果为有害		海藻糖酶缺乏症
CDK5RAP2	chr9:123301413	c.413G>A(E6)	p.138,G>D			杂合	可能相关	蛋白结构预测结果为有害		常染色体隐性原发性小头畸形3型,AR
SEC23A	chr14:39502520	c.2221C>G(E20)	p.741,P>A			杂合	可能相关	蛋白结构预测结果为有害		颅额鼻发育不良,AR
STXBP2	chr19:7706658	c.497C>T(E7)	p.166,T>M	rs181216956	0.0026	杂合	可能相关	蛋白结构预测结果为有害		噬血细胞淋巴组织细胞增生症5型
HAGH	chr16:1872946	c.25C>G(E3)	p.9,L>V			杂合	可能相关	蛋白结构预测结果为有害		羟酰谷胱甘肽水解酶缺陷症,AD
CRLF1	chr19:18710461	c.311A>G(E2)	p.104,N>S	rs117193413	0.0042	杂合	可能相关	蛋白结构预测结果为有害		冷引导发汗综合征1型,AR
CHD7	chr8:61655556	c.1565G>T(E2)	p.522,G>V	rs142962579	0.0062	杂合	可能相关	蛋白结构预测结果为有害		先天性脊柱侧凸易感3型;促性腺激素分泌不足的性腺功能低下症,伴或不伴嗅觉丧失5型;霍尔希特纳综合征,AD
NEB	chr2:152420386	c.18530G>A(E118)	p.6177,R>H	rs147159176	0.0032	杂合	可能相关	蛋白结构预测结果为有害		2型杆状体肌病,AR
SMPD1	chr11:6411936-6411941	c.108(E1)至c.113(E1):缺失GCTGGC	缺失非移码	rs558809956		杂合	可能相关	蛋白结构预测结果为有害		尼曼-匹克病B型,AR;尼曼-匹克病A型,AR
MAMLD1	chrX:149638177	c.332C>A(E3)	p.111,P>H			半合子	可能相关	蛋白结构预测结果为有害		X-连锁尿道下裂2型,XR
ADCY1	chr7:45717651	c.1789C>T(E9)	p.597,R>W	rs142005939	0.0018	杂合	可能相关	蛋白结构预测结果为有害		常染色隐性遗传性耳聋44型,AR

基因突变及良性多态性变异:

基因	染色体位置	核酸改变	氨基酸改变	rs编号	MAF	杂合/纯合	与疾病相关性	相关性说明	PubMed文献号	基因功能或关联疾病表型
STAT5B	chr17:40354410	c.2185G>T(E18)	p.729,A>S	rs200042237		杂合	可能相关	蛋白结构预测结果为有害		伴免疫系统缺陷的生长激素不敏感综合症
RNF213	chr17:78298927	c.3122C>T(E18)	p.1041,A>V	rs138825989	0.0012	杂合	可能相关	蛋白结构预测结果为有害		烟雾病2型/毛毛样脑血管病2型
CFH	chr1:196706049	c.2509G>A(E16)	p.837,V>I	rs55807605	0.0034	杂合	可能相关	蛋白结构预测结果为有害		年龄相关性黄斑变性4型;基底膜板层状玻璃膜疣,AD
F11	chr4:187195378	c.434A>G(E5)	p.145,H>R	rs199657604	0.0014	杂合	可能相关	蛋白结构预测结果为有害		遗传性凝血因子XI缺乏症
POLH	chr6:43581585	c.1433C>T(E11)	p.478,T>M	rs9296419	0.0058	杂合	可能相关	蛋白结构预测结果为有害		着色性干皮病,变异型,AR
PPARGC1B	chr5:149212358	c.722G>A(E5)	p.241,R>Q	rs201009390	0.0006	杂合	可能相关	蛋白结构预测结果为有害		肥胖（易感）
	chr5:149206344	c.361G>A(E3)	p.121,A>T	rs374316596		杂合	可能相关	蛋白结构预测结果为有害		
NNT	chr5:43628483	c.958A>G(E7)	p.320,I>V	rs200849046	0.0002	杂合	可能相关	蛋白结构预测结果为有害		糖皮质激素缺乏症4型,AR
GIGYF2	chr2:233712232-233712233	c.3635(E27)至c.3636(E27):插入GCA	插入非移码	rs58340018		杂合	可能相关	蛋白结构预测结果为有害		帕金森病11型
ACE	chr17:61561870	c.1889C>T(E12)	p.630,P>L	rs142818229		杂合	可能相关	蛋白结构预测结果为有害		心肌梗塞,易感1型;肾小管发育不良,AR;脑出血、中风（易感）;阿尔茨海默病（老年性痴呆）（易感）,AD;糖尿病微血管并发症,易感3型
GRXCR1	chr4:42964918	c.394A>G(E2)	p.132,T>A			杂合	可能相关	蛋白结构预测结果为有害		常染色体隐性遗传耳聋25型

基因突变及良性多态性变异:

基因	染色体位置	核酸改变	氨基酸改变	rs编号	MAF	杂合/纯合	与疾病相关性	相关性说明	PubMed文献号	基因功能或关联疾病表型
ATP13A2	chr1:17320210	c.1663C>G(E16)	p.555,L>V			杂合	可能相关	蛋白结构预测结果为有害		Kufor-Rakeb病,AR
ZFHX4	chr8:77765012	c.5855G>A(E10)	p.1952,G>D	rs74655788	0.001	杂合	可能相关	蛋白结构预测结果为有害		先天性遗传性上睑下垂症1型,AD
PHKB	chr16:47683077	c.1738A>C(E19)	p.580,M>L	rs140636792	0.001	杂合	可能相关	蛋白结构预测结果为有害		糖原累积症IXb型（肝）,AR
TTLL5	chr14:76201564	c.1213C>T(E15)	p.405,R>W	rs201356863	0.0002	杂合	可能相关	蛋白结构预测结果为有害		视锥-视杆细胞营养不良19型,AR
FANCE	chr6:35426132	c.1028G>A(E5)	p.343,R>Q	rs45467798	0.0042	杂合	可能相关	蛋白结构预测结果为有害		范可尼贫血,互补E组,AR
PIEZO1	chr16:88787652	c.5590C>T(E39)	p.1864,R>C	rs79879471	0.0004	杂合	可能相关	蛋白结构预测结果为有害		（脱水型）遗传性口形红细胞增多症伴或不伴假性高钾血症和/或围产期水肿,AD
RPGRIP1	chr14:21762877	c.127C>T(E2)	p.43,R>W			杂合	可能相关	蛋白结构预测结果为有害		视锥细胞与视锥杆细胞营养不良13型;先天性黑蒙症6型
SGCD	chr5:156186376	c.848A>G(E9)	p.283,Q>R	rs397516338		杂合	可能相关	蛋白结构预测结果为有害		扩张性心肌病1L型;肢带型肌营养不良症2F型
TG	chr8:133918963	c.3665C>T(E17)	p.1222,S>L	rs12549018	0.0064	杂合	可能相关	蛋白结构预测结果为有害		自身免疫性甲状腺疾病,易感3型;甲状腺激素生成障碍3型,AR
COL3A1	chr2:189871110	c.3133G>A(E43)	p.1045,A>T	rs149722210	0.0018	杂合	可能相关	蛋白结构预测结果为有害		爱唐综合症(皮肤弹力过度症)4型（血管型）,AD;爱唐综合症(皮肤弹力过度症)3型（运动过度型）,AD
	chr2:189861949	IVS25+5G>A	剪切位点	rs146652498	0.0018	杂合	可能相关	mRNA剪接可能会受影响		
MIF	chr22:24236731	c.70A>T(E1)	p.24,T>S	rs200995600		杂合	可能相关	蛋白结构预测结果为有害		全身型青少年类风湿性关节炎

基因突变及良性多态性变异:

基因	染色体位置	核酸改变	氨基酸改变	rs编号	MAF	杂合/纯合	与疾病相关性	相关性说明	PubMed文献号	基因功能或关联疾病表型
FAT4	chr4:126372975	c.10804A>C(E9)	p.3602,I>L	rs76491994	0.0096	杂合	可能相关	蛋白结构预测结果为有害		Van Maldergem综合征2型,AR;Hennekam淋巴管扩张-淋巴水肿综合征2型,AR
SETBP1	chr18:42618459	c.4010G>C(E5)	p.1337,S>T	rs529611461	0.0004	杂合	可能相关	蛋白结构预测结果为有害		常染色体显性遗传性精神发育迟滞29型,AD;Schinzel-Giedion面中部回缩综合征,AD
	chr18:42531184	c.1879C>T(E4)	p.627,R>C	rs146193261	0.01	杂合	可能相关	蛋白结构预测结果为有害		
STAT3	chr17:40467780	c.2296G>A(E24)	p.766,A>T			杂合	可能相关	蛋白结构预测结果为有害		复发感染型高IgE综合征/job综合征（显性遗传）/常染色体显性高lgE综合征,AD;婴儿期发病的多系统自身免疫性疾病,AD
TUBA8	chr22:18609647	c.902A>G(E4)	p.301,Q>R	rs2234333	0.0018	纯合	可能相关	蛋白结构预测结果为有害		多小脑回畸形合并视神经发育不良,AR
COG5	chr7:107167769	c.544A>G(E6)	p.182,I>V	rs79413133	0.0052	杂合	可能相关	蛋白结构预测结果为有害		先天性糖基化病2I型
ABCC2	chr10:101595954	c.3521G>A(E25)	p.1174,R>H	rs139188247		杂合	可能相关	蛋白结构预测结果为有害		杜宾约翰逊综合征/Dubin-Johnson综合征/遗传性结合型胆红素增高症1型,AR
VLDLR	chr9:2647560	c.1790C>T(E12)	p.597,A>V	rs369741873		杂合	可能相关	蛋白结构预测结果为有害		小脑性共济失调、智力低下和平衡失调综合征1型,AR
TTN	chr2:179518937	c.38378A>G(E193)	p.12793,K>R	rs189389531	0.0034	杂合	可能相关	蛋白结构预测结果为有害		迟发性胫骨肌营养不良症,AD;肢带型肌营养不良症2J型,AR;家族性肥厚型心肌病9型;先天性肌病,早发,伴心肌病,AR;遗[illegible]
	chr2:179615663	c.11464C>T(E46)	p.3822,H>Y			杂合	可能相关	蛋白结构预测结果为有害		

基因突变及良性多态性变异:

基因	染色体位置	核酸改变	氨基酸改变	rs编号	MAF	杂合/纯合	与疾病相关性	相关性说明	PubMed文献号	基因功能或关联疾病表型
	chr2:179483183	c.47002G>C(E252)	p.15668,E>Q			杂合	可能相关	蛋白结构预测结果为有害		传性疾病伴早期呼吸衰竭;扩张型心肌病1G型
SETX	chr9:135218144	c.431A>G(E5)	p.144,N>S			杂合	可能相关	蛋白结构预测结果为有害		早发型共济失调伴动眼不能和低白蛋白血症2型,AR;肌萎缩性侧索硬化,幼年4型,AD

基因突变及良性多态性变异:

基因	染色体位置	核酸改变	氨基酸改变	rs编号	MAF	杂合/纯合	与疾病相关性	相关性说明	PubMed文献号	基因功能或关联疾病表型
CRYGD	chr2:208988933	c.155C>G(E2)	p.52,S>W			杂合	可能相关	蛋白结构预测结果为有害		白内障4型，多种型,AD
	chr2:208988920	c.168C>G(E2)	p.56,Y>X(119)	rs202233735	0.0036	杂合	较高	无义突变可能会导致蛋白翻译提前终止		
ORC4	chr2:148710067	c.463A>C(E9)	p.155,I>L	rs534080667	0.0002	杂合	可能相关	蛋白结构预测结果为有害		MEIER-GORLIN综合征2型,AR
FGF23	chr12:4479706	c.559C>G(E3)	p.187,R>G	rs190841442	0.0008	杂合	可能相关	蛋白结构预测结果为有害		家族性瘤样钙化高磷酸盐血症,AR;常显性遗传低血磷性佝偻病,AD
MOGS	chr2:74689855	c.1061G>A(E4)	p.354,G>D	rs200348131	0.0002	杂合	可能相关	蛋白结构预测结果为有害		先天性糖基化病2B型,AR
RIPK4	chr21:43161057	c.2296C>G(E8)	p.766,L>V	rs200025631	0.0006	杂合	可能相关	蛋白结构预测结果为有害		致命性国(腿弯)翼状胬肉综合征,AR
ITGAM	chr16:31341863	c.3216G>C(E28)	p.1072,E>D	rs140927329	0.0018	杂合	可能相关	蛋白结构预测结果为有害		系统性红斑狼疮,易感6型,ITGAM突变型
DAOA	chr13:106124937	c.184A>G(E3)	p.62,K>E	rs9558562	0.0092	杂合	可能相关	蛋白结构预测结果为有害		精神分裂症,AD
B3GNT1	chr11:66114662	c.355C>A(E1)	p.119,P>T	rs201892419	0.0006	杂合	可能相关	蛋白结构预测结果为有害		A13型肌-眼脑病（先天性肌营养不良伴眼、脑异常）,AR
SFTPB	chr2:85894807	c.226G>A(E3)	p.76,G>R	rs148914290	0.0006	杂合	可能相关	蛋白结构预测结果为有害		肺泡蛋白沉着症1型/非特异性间质肺炎,AR
RFX5	chr1:151315284	c.1229G>A(E11)	p.410,R>Q			杂合	可能相关	蛋白结构预测结果为有害		裸淋巴细胞综合征2型,AR
CPS1	chr2:211521316	c.3626T>C(E30)	p.1209,M>T	rs200569046	0.001	杂合	可能相关	蛋白结构预测结果为有害		尿素循环障碍/氨甲酰磷酸合成酶I缺乏症,AR;新生儿肺动脉高压（易感）

基因突变及良性多态性变异:

基因	染色体位置	核酸改变	氨基酸改变	rs编号	MAF	杂合/纯合	与疾病相关性	相关性说明	PubMed文献号	基因功能或关联疾病表型
SLC4A11	chr20:3209897	c.1991G>A(E16)	p.664,R>H			杂合	可能相关	蛋白结构预测结果为有害		Fuchs角膜内皮营养不良4型;角膜营养不良和感知性耳聋,AR;角膜内皮营养不良2型,AR
FLG	chr1:152275982	c.11380C>A(E3)	p.3794,Q>K	rs562201369	0.0002	杂合	可能相关	蛋白结构预测结果为有害		寻常性鱼鳞病,AD;特应性皮炎(湿疹)2型
MUC5B	chr11:1269557	c.11447C>T(E31)	p.3816,T>M	rs201948297	0.0014	杂合	可能相关	蛋白结构预测结果为有害		特发性肺纤维化易感,AD
C5orf42	chr5:37226814	c.1883G>A(E12)	p.628,R>K	rs74975451	0.0074	杂合	可能相关	蛋白结构预测结果为有害		Joubert综合征17型,AR;口-面-指综合征6型,AR
HRG	chr3:186386733	c.193A>G(E2)	p.65,T>A			杂合	可能相关	蛋白结构预测结果为有害		富组氨酸糖蛋白缺乏性易栓症
	chr3:186387779	c.353A>G(E3)	p.118,D>G	rs3733008	0.0078	杂合	可能相关	蛋白结构预测结果为有害		
INF2	chr14:105180903	c.3404C>T(E21)	p.1135,T>M	rs3803311	0.009	杂合	可能相关	蛋白结构预测结果为有害		局灶性节段性肾小球硬化症5型;恰克-马利-杜斯氏症,显性中间E型,AD
CR1	chr1:207751179	c.4567G>A(E29)	p.1523,A>T	rs187750583	0.0012	杂合	可能相关	蛋白结构预测结果为有害		疟疾,易感
CENPJ	chr13:25478083	c.2806A>G(E8)	p.936,S>G	rs75008861	0.005	杂合	可能相关	蛋白结构预测结果为有害		常染色体隐性原发性小头畸形6型,AR;Seckel综合征4,AR
NAGA	chr22:42459035	IVS6-7C>A	剪切位点	rs150693978	0.0052	杂合	可能相关	mRNA剪接可能会受影响		Kanzaki病,AR;Schindler病I型和III型,AR
LEMD3	chr12:65632449	c.1776G>A(E6)	p.592,R>R			杂合	可能相关	mRNA剪接可能会受影响		Buschke-Ollendorff综合征,AD;肢骨纹状肥大

基因突变及良性多态性变异:

基因	染色体位置	核酸改变	氨基酸改变	rs编号	MAF	杂合/纯合	与疾病相关性	相关性说明	PubMed文献号	基因功能或关联疾病表型
POLG	chr15:89864246	IVS17-3C>A	剪切位点			杂合	可能相关	mRNA剪接可能会受影响		进行性眼外肌麻痹合并线粒体核酸缺失1型,AD;线粒体DNA缺失综合征4B型（线粒体性神经胃肠脑病变综合征型）,AR;感觉性共济失调性周围神经病伴构音障碍及眼肌麻痹(SANDO),AR;进行性眼外肌麻痹合并线粒体核酸缺失（隐性遗传）,AR;弥漫性进行性脑灰质变性综合症/阿尔帕斯氏综合征/线粒体DNA缺失综合征4A,AR
	chr15:89864088	c.2890C>T(E18)	p.964,R>C	rs201477273	0.003	杂合	可能相关	蛋白结构预测结果为有害		
ROBO2	chr3:75986622	IVS1-10C>G	剪切位点	rs9631539		杂合	可能相关	mRNA剪接可能会受影响		膀胱输尿管返流2型
	chr3:75986663	c.19C>A(E2)	p.7,R>S	rs12171318		杂合	可能相关	蛋白结构预测结果为有害		
IFNGR2	chr21:34799345	IVS4+6A>C	剪切位点	rs184664014	0.0008	杂合	可能相关	mRNA剪接可能会受影响		免疫缺陷28
DNAH11	chr7:21856163	c.10433G>A(E64)	p.3478,R>Q	rs117729990	0.0032	杂合	未知	未知		原发性纤毛运动障碍7型（伴或不伴内脏异位）,AR
DNMT3B	chr20:31375113-31375121	c.384(E5)至c.392(E5):缺失CACAGACGA	缺失非移码			杂合	未知	未知		免疫机能丧失,着丝粒的不稳定和面部畸形综合症1型,AR
SHANK2	chr11:70666489	c.1327G>A(E12)	p.443,A>T			杂合	未知	未知		自闭症,易感17型
	chr11:70332443	c.3809G>A(E25)	p.1270,R>H			杂合	可能相关	蛋白结构预测结果为有害		
COL18A1	chr21:46932115	c.4363G>T(E41)	p.1455,V>L	rs61736805	0.018	杂合	未知	未知		Knobloch综合症1型/视网膜剥离和枕部脑膨出综合征,AR

基因突变及良性多态性变异:

基因	染色体位置	核酸改变	氨基酸改变	rs编号	MAF	杂合/纯合	与疾病相关性	相关性说明	PubMed文献号	基因功能或关联疾病表型
PRKDC	chr8:48746866	c.8042C>T(E59)	p.2681,P>L	rs200543348		杂合	未知	未知		免疫缺陷26，伴或不伴神经系统异常,AR
NUP210L	chr1:154002449	c.3778A>G(E28)	p.1260,T>A			杂合	可能相关	蛋白结构预测结果为有害		
C11orf21	chr11:2320865-2320866	c.357(E4)至c.358(E4):插入C	p.120,T>Hfs4	rs3214127	0.005	杂合	较高	理论上这种移码突变会导致蛋白序列明显改变		
MPND	chr19:4359190	c.1267C>T (E11)	p.423,P>S	rs200972486	0.0014	杂合	可能相关	蛋白结构预测结果为有害		
MAPK8IP2	chr22:51042513-51042514	c.785(E5)至c.786(E5):插入G	p.262,A>Afs183	rs35700889		纯合	较高	理论上这种移码突变会导致蛋白序列明显改变		
OR3A2	chr17:3181827	c.403A>G(E1)	p.135,I>V			杂合	可能相关	蛋白结构预测结果为有害		
TMEM44	chr3:194337905	c.847G>A(E7)	p.283,D>N	rs201921184	0.0028	杂合	可能相关	蛋白结构预测结果为有害		
MLNR	chr13:49795272	c.799T>C(E1)	p.267,Y>H	rs184614513	0.0012	杂合	可能相关	蛋白结构预测结果为有害		
NPIP	chr16:15045766	c.937C>T(E8)	p.313,L>F	rs147546015		杂合	可能相关	蛋白结构预测结果为有害		
ATXN7L2	chr1:110034254	c.2069G>A(E10)	p.690,G>D			杂合	可能相关	蛋白结构预测结果为有害		
RUFY4	chr2:218940170	c.955G>T(E9)	p.319,V>F	rs192336087	0.0096	杂合	可能相关	蛋白结构预测结果为有害		
UBE4B	chr1:10166271	c.826C>T(E7)	p.276,P>S	rs200980839	0.0006	杂合	可能相关	蛋白结构预测结果为有害		

基因突变及良性多态性变异:

基因	染色体位置	核酸改变	氨基酸改变	rs编号	MAF	杂合/纯合	与疾病相关性	相关性说明	PubMed文献号	基因功能或关联疾病表型
TONSL	chr8:145661967-145661967	c.1988(E16):缺失T	p.663,L>Rfs33			杂合	较高	理论上这种移码突变会导致蛋白序列明显改变		
LTK	chr15:41797721	c.1705C>A(E14)	p.569,R>S	rs148513655	0.0066	杂合	未知	蛋白结构预测结果为容忍或无害		
NGEF	chr2:233835009	c.298G>A(E3)	p.100,E>K			杂合	可能相关	蛋白结构预测结果为有害		
PTPRB	chr12:70964983	c.3193G>A(E13)	p.1065,D>N			杂合	可能相关	蛋白结构预测结果为有害		
NEFM	chr8:24772189	c.883C>A(E1)	p.295,R>S	rs201666371	0.0002	杂合	可能相关	蛋白结构预测结果为有害		
TNRC6A	chr16:24801671	c.1708G>A(E6)	p.570,G>R	rs555251368	0.0002	杂合	可能相关	蛋白结构预测结果为有害		
TMEM87A	chr15:42560219	c.217C>A(E3)	p.73,P>T			杂合	可能相关	蛋白结构预测结果为有害		
NPTX2	chr7:98248949	IVS1-6T>C	剪切位点	rs186876608	0.001	杂合	可能相关	mRNA剪接可能会受影响		
N4BP2	chr4:40122958	c.3227C>T(E9)	p.1076,T>M	rs183255501	0.0004	杂合	可能相关	蛋白结构预测结果为有害		
CCDC63	chr12:111291236	c.37G>A(E3)	p.13,D>N	rs114761668	0.0022	杂合	可能相关	蛋白结构预测结果为有害		
TPM4	chr19:16178470	c.36G>C(E1)	p.12,K>N	rs201430950	0.0004	杂合	可能相关	蛋白结构预测结果为有害		
DNAH7	chr2:196801500	c.3095C>T(E20)	p.1032,T>I			杂合	可能相关	蛋白结构预测结果为有害		
TMEM156	chr4:38990575	c.635T>C(E4)	p.212,M>T	rs2276887	0.0062	纯合	可能相关	蛋白结构预测结果为有害		

基因突变及良性多态性变异:

基因	染色体位置	核酸改变	氨基酸改变	rs编号	MAF	杂合/纯合	与疾病相关性	相关性说明	PubMed文献号	基因功能或关联疾病表型
C20orf141	chr20:2796077	c.247G>T(E1)	p.83,D>Y			杂合	可能相关	蛋白结构预测结果为有害		
SLC37A3	chr7:140043294	c.1244T>C(E13)	p.415,L>P	rs186668396	0.0048	杂合	可能相关	蛋白结构预测结果为有害		
FAM173B	chr5:10236627	c.407A>G(E3)	p.136,H>R	rs140073596	0.0004	杂合	可能相关	蛋白结构预测结果为有害		
ING5	chr2:242650860	c.345G>A(E4)	p.115,M>I	rs185566428	0.0006	杂合	可能相关	蛋白结构预测结果为有害		
KRTAP1-5	chr17:39183037	c.371G>A(E1)	p.124,R>H	rs148354281	0.0066	杂合	可能相关	蛋白结构预测结果为有害		
WNT11	chr11:75905835	c.373G>A(E3)	p.125,A>T	rs201061165	0.0026	杂合	可能相关	蛋白结构预测结果为有害		
S100A7A	chr1:153390601-153390601	c.43(E2):缺失G	p.15,D>Tfs19			杂合	较高	理论上这种移码突变会导致蛋白序列明显改变		
TYSND1	chr10:71902429	c.1478T>A(E3)	p.493,L>H			杂合	可能相关	蛋白结构预测结果为有害		
XIRP2	chr2:168103418	c.5516G>A(E9)	p.1839,G>D	rs77219745	0.01	杂合	可能相关	蛋白结构预测结果为有害		
	chr2:168101570	c.3668T>C(E9)	p.1223,I>T	rs75802875	0.01	杂合	可能相关	蛋白结构预测结果为有害		
DDR1	chr6:30862414	c.1397G>A(E10)	p.466,R>H	rs201231834	0.0002	杂合	可能相关	蛋白结构预测结果为有害		
NRIP1	chr21:16337111	c.3403C>T(E4)	p.1135,R>C	rs61750207	0.0044	杂合	可能相关	蛋白结构预测结果为有害		
	chr21:16338136	c.2378C>T(E4)	p.793,P>L			杂合	可能相关	蛋白结构预测结果为有害		

基因突变及良性多态性变异:

基因	染色体位置	核酸改变	氨基酸改变	rs编号	MAF	杂合/纯合	与疾病相关性	相关性说明	PubMed文献号	基因功能或关联疾病表型
PCDHGB4	chr5:140767890	c.439C>T(E1)	p.147,Q>X(777)	rs534112048	0.0004	杂合	较高	无义突变可能会导致蛋白翻译提前终止		
EML5	chr14:89131795	c.3163T>G(E22)	p.1055,L>V	rs373476855	0.0002	杂合	可能相关	蛋白结构预测结果为有害		
MCM3	chr6:52141073	c.1502A>G(E9)	p.501,Y>C			杂合	可能相关	蛋白结构预测结果为有害		
NPAS4	chr11:66191310	c.949A>C(E7)	p.317,M>L	rs76159120	0.0022	杂合	可能相关	蛋白结构预测结果为有害		
CHST5	chr16:75563732	c.551G>A(E3)	p.184,R>Q	rs201072221	0.0088	杂合	可能相关	蛋白结构预测结果为有害		
SEZ6L	chr22:26688840	c.563G>A(E2)	p.188,R>Q	rs146205533	0.006	杂合	可能相关	蛋白结构预测结果为有害		
FAM186B	chr12:49982238	c.2533C>T(E6)	p.845,R>C	rs76224014	0.0056	杂合	可能相关	蛋白结构预测结果为有害		
PEBP4	chr8:22675244	c.263C>G(E4)	p.88,A>G	rs117235442	0.0062	杂合	可能相关	蛋白结构预测结果为有害		
NOL8	chr9:95076749	c.1954C>G(E8)	p.652,L>V			杂合	可能相关	蛋白结构预测结果为有害		
NLRP9	chr19:56243576	c.1621G>C(E2)	p.541,A>P	rs117621173	0.0032	杂合	可能相关	蛋白结构预测结果为有害		
DMRT3	chr9:990401	c.815A>C(E2)	p.272,K>T	rs187176004	0.002	杂合	可能相关	蛋白结构预测结果为有害		
LEPREL2	chr12:6938023-6938024	c.418(E1)至c.419(E1):插入G	p.140,R>Rfs57			杂合	较高	理论上这种移码突变会导致蛋白序列明显改变		

基因突变及良性多态性变异:

基因	染色体位置	核酸改变	氨基酸改变	rs编号	MAF	杂合/纯合	与疾病相关性	相关性说明	PubMed文献号	基因功能或关联疾病表型
FAM58BP	chr1:200182710-200182721	c.19(E1)至c.30(E1):缺失GCCGGAGAGGAG	缺失非移码	rs565394962	0.039	杂合	可能相关	蛋白结构预测结果为有害		
KLK14	chr19:51582802	c.418G>A(E5)	p.140,G>R	rs199715229	0.0024	杂合	可能相关	蛋白结构预测结果为有害		
MDN1	chr6:90359865	c.16084A>G(E97)	p.5362,I>V	rs373778917		杂合	可能相关	蛋白结构预测结果为有害		
BOC	chr3:113003262	c.2734C>T(E17)	p.912,P>S	rs3814404	0.0076	杂合	可能相关	蛋白结构预测结果为有害		
	chr3:113005527	c.3163C>T(E20)	p.1055,P>S			杂合	可能相关	蛋白结构预测结果为有害		
PSMD4	chr1:151234652	c.42G>T(E2)	p.14,E>D			杂合	可能相关	蛋白结构预测结果为有害		
LTF	chr3:46506310	IVS1+5G>A	剪切位点	rs200665317	0.0004	杂合	可能相关	mRNA剪接可能会受影响		
KLHL28	chr14:45414689	c.443G>A(E2)	p.148,G>D			杂合	可能相关	蛋白结构预测结果为有害		
EMID1	chr22:29630183	c.1024G>A(E11)	p.342,E>K	rs377281956		杂合	可能相关	蛋白结构预测结果为有害		
CCT8L2	chr22:17073274	c.167G>A(E1)	p.56,R>Q	rs567911712	0.0004	杂合	可能相关	蛋白结构预测结果为有害		
ARHGAP20	chr11:110451725	c.1876G>A(E15)	p.626,V>I	rs190953225	0.002	杂合	可能相关	蛋白结构预测结果为有害		
DPY19L3	chr19:32902223	c.182C>T(E3)	p.61,T>I	rs576952552	0.0002	杂合	可能相关	蛋白结构预测结果为有害		

基因突变及良性多态性变异:

基因	染色体位置	核酸改变	氨基酸改变	rs编号	MAF	杂合/纯合	与疾病相关性	相关性说明	PubMed文献号	基因功能或关联疾病表型
DPY19L3	chr19:32902198	c.157A>G(E3)	p.53,I>V	rs201012525	0.0006	杂合	可能相关	蛋白结构预测结果为有害		
ARHGAP18	chr6:129920446	c.1628A>C(E12)	p.543,K>T			杂合	可能相关	蛋白结构预测结果为有害		
ZCWPW1	chr7:100013609	c.740T>G(E8)	p.247,I>R	rs149367938	0.006	杂合	可能相关	蛋白结构预测结果为有害		
TLN2	chr15:63128231	c.7333G>T(E54)	p.2445,V>L	rs78592652	0.0034	杂合	未知	蛋白结构预测结果为容忍或无害		
POU6F1	chr12:51590593	c.34G>A(E2)	p.12,A>T	rs77905600	0.0074	杂合	可能相关	蛋白结构预测结果为有害		
HNRNPA1L2	chr13:53216887	c.260A>C(E7)	p.87,K>T	rs561271617	0.0002	杂合	可能相关	蛋白结构预测结果为有害		
MTOR	chr1:11190698-11190709	c.5490(E39)至c.5501(E39):缺失TGCCGCCACCAC	缺失非移码	rs571156267	0.001	杂合	可能相关	蛋白结构预测结果为有害		
ITGAD	chr16:31422174	c.1331A>G(E12)	p.444,K>R			杂合	可能相关	蛋白结构预测结果为有害		
LTB4R2	chr14:24780337	c.467C>T(E2)	p.156,A>V	rs375901905		杂合	可能相关	蛋白结构预测结果为有害		
GLS	chr2:191746076	c.266A>C(E1)	p.89,H>P	rs143584207	0.012	杂合	可能相关	蛋白结构预测结果为有害		
ULK2	chr17:19687216	c.2254G>A(E22)	p.752,G>R	rs55730189	0.0058	杂合	可能相关	蛋白结构预测结果为有害		
PLEKHA7	chr11:17035678	c.157C>T(E2)	p.53,R>C	rs201642255	0.0012	杂合	可能相关	蛋白结构预测结果为有害		

基因突变及良性多态性变异:

基因	染色体位置	核酸改变	氨基酸改变	rs编号	MAF	杂合/纯合	与疾病相关性	相关性说明	PubMed文献号	基因功能或关联疾病表型
NXN	chr17:882626	c.293G>A(E1)	p.98,R>Q			杂合	可能相关	蛋白结构预测结果为有害		
TET3	chr2:74273945	c.496C>T(E1)	p.166,R>W	rs369970162		杂合	可能相关	蛋白结构预测结果为有害		
SREBF2	chr22:42264732	c.656C>T(E3)	p.219,T>M	rs201991869	0.0008	杂合	可能相关	蛋白结构预测结果为有害		
OR11H1	chr22:16449784	c.21G>T(E1)	p.7,Q>H	rs200562384		杂合	可能相关	蛋白结构预测结果为有害		
HEATR1	chr1:236740100	c.2905G>A(E21)	p.969,D>N	rs141424510	0.001	杂合	可能相关	蛋白结构预测结果为有害		
KCTD19	chr16:67333285	c.967G>A(E6)	p.323,V>I	rs370173436		杂合	可能相关	蛋白结构预测结果为有害		
ZNF830	chr17:33288637	c.52C>A(E1)	p.18,Q>K	rs200110878	0.0004	杂合	可能相关	蛋白结构预测结果为有害		
ACY3	chr11:67410339	c.816T>A(E8)	p.272,Y>X(48)	rs188330165	0.0002	杂合	较高	无义突变可能会导致蛋白翻译提前终止		
TBL2	chr7:72984918-72984920	c.1261(E7)至c.1263(E7):缺失AAC	缺失非移码	rs202195176	0.0034	杂合	可能相关	蛋白结构预测结果为有害		
LPO	chr17:56327932	c.730G>A(E7)	p.244,A>T	rs8178338	0.0054	杂合	可能相关	蛋白结构预测结果为有害		
OBSCN	chr1:228509763	c.18092G>A(E66)	p.6031,R>H	rs570286923	0.0002	杂合	可能相关	蛋白结构预测结果为有害		
ENTPD2	chr9:139946719	c.199G>A(E2)	p.67,G>S			杂合	可能相关	蛋白结构预测结果为有害		
CLASP2	chr3:33558573	c.3908A>G(E36)	p.1303,Y>C	rs183373873	0.0022	杂合	可能相关	蛋白结构预测结果为有害		

基因突变及良性多态性变异:

基因	染色体位置	核酸改变	氨基酸改变	rs编号	MAF	杂合/纯合	与疾病相关性	相关性说明	PubMed文献号	基因功能或关联疾病表型
TMEM192	chr4:166009677	c.517C>A(E4)	p.173,L>M	rs528271478		杂合	可能相关	蛋白结构预测结果为有害		
SLC30A5	chr5:68411954	c.985C>T(E9)	p.329,R>W	rs142663787	0.0064	杂合	可能相关	蛋白结构预测结果为有害		
MTCH1	chr6:36953920-36953920	c.30(E1):缺失C	p.11,W>Gfs82	rs35538959	0.047	杂合	较高	理论上这种移码突变会导致蛋白序列明显改变		
B3GNT6	chr11:76750639-76750645	c.44(E2)至c.50(E2):缺失CCTGCCT	p.15,A>Afs5			杂合	较高	理论上这种移码突变会导致蛋白序列明显改变		
CSE1L	chr20:47685270	c.586A>G(E7)	p.196,S>G	rs150139793	0.0006	杂合	可能相关	蛋白结构预测结果为有害		
TRIM68	chr11:4626335	c.400A>G(E2)	p.134,M>V			杂合	可能相关	蛋白结构预测结果为有害		
PRAMEF5	chr1:13365987	c.431A>G(E3)	p.144,Q>R	rs199741513		杂合	可能相关	蛋白结构预测结果为有害		
PRAMEF6	chr1:13001186	c.497G>A(E3)	p.166,C>Y	rs200795385		杂合	未知	蛋白结构预测结果为容忍或无害		
UBE2D3	chr4:103720139	IVS6-5A>G	剪切位点	rs141984049	0.0008	杂合	可能相关	mRNA剪接可能会受影响		
PRAMEF4	chr1:12939476	c.1326C>G(E4)	p.442,N>K	rs149134171		杂合	可能相关	蛋白结构预测结果为有害		
CMPK1	chr1:47838652	c.344A>G(E3)	p.115,N>S	rs72553947	0.0058	杂合	可能相关	蛋白结构预测结果为有害		
SSH1	chr12:109186093	c.1862A>G(E14)	p.621,N>S	rs117900986	0.009	杂合	可能相关	蛋白结构预测结果为有害		

基因突变及良性多态性变异:

基因	染色体位置	核酸改变	氨基酸改变	rs编号	MAF	杂合/纯合	与疾病相关性	相关性说明	PubMed文献号	基因功能或关联疾病表型
ZNF844	chr19:12187830	c.1895C>A(E4)	p.632,T>K	rs181157360	0.0004	杂合	可能相关	蛋白结构预测结果为有害		
NYAP1	chr7:100087058	c.1714G>A(E4)	p.572,V>M	rs199772550	0.001	杂合	可能相关	蛋白结构预测结果为有害		
AKAP6	chr14:33004989	c.554C>T(E3)	p.185,A>V			杂合	可能相关	蛋白结构预测结果为有害		
RALGAPA1	chr14:36191031	c.2129T>C(E16)	p.710,V>A	rs75856669	0.0058	杂合	可能相关	蛋白结构预测结果为有害		
KRTAP4-9	chr17:39261686	c.46G>A(E1)	p.16,G>S	rs199817117		杂合	可能相关	蛋白结构预测结果为有害		
PRAMEF13	chr1:13448199	c.1276G>A(E4)	p.426,D>N	rs143762964		杂合	可能相关	蛋白结构预测结果为有害		
SLITRK2	chrX:144906464	c.2521G>T(E5)	p.841,A>S	rs189172454		半合子	可能相关	蛋白结构预测结果为有害		
FAM118A	chr22:45723947	IVS5+3A>G	剪切位点	rs74676649		杂合	可能相关	mRNA剪接可能会受影响		
PICK1	chr22:38471068	c.1177G>A(E13)	p.393,G>R	rs187753076	0.002	杂合	可能相关	蛋白结构预测结果为有害		
MADD	chr11:47311593	c.2992A>G(E18)	p.998,I>V			杂合	可能相关	蛋白结构预测结果为有害		
SPARCL1	chr4:88415038	c.914T>C(E5)	p.305,I>T	rs117758185	0.0066	杂合	可能相关	蛋白结构预测结果为有害		
FAM82A1	chr2:38178747	c.389G>C(E2)	p.130,R>P			杂合	可能相关	蛋白结构预测结果为有害		
RRP1	chr21:45217979	c.809C>T(E8)	p.270,A>V	rs117749073	0.0042	杂合	可能相关	蛋白结构预测结果为有害		

基因突变及良性多态性变异:

基因	染色体位置	核酸改变	氨基酸改变	rs编号	MAF	杂合/纯合	与疾病相关性	相关性说明	PubMed文献号	基因功能或关联疾病表型
INADL	chr1:62579839-62579841	c.4576(E35)至c.4578(E35):缺失GAG	缺失非移码	rs149912818	0.0074	杂合	可能相关	蛋白结构预测结果为有害		
TOR1AIP2	chr1:179815378	c.1241C>T(E7)	p.414,T>M	rs151254233		杂合	可能相关	蛋白结构预测结果为有害		
DSCAM	chr21:41719720	c.1087A>G(E6)	p.363,T>A	rs537698010	0.0004	杂合	可能相关	蛋白结构预测结果为有害		
POC5	chr5:74998606	c.337C>T(E5)	p.113,P>S	rs79392300	0.01	杂合	可能相关	蛋白结构预测结果为有害		
TMPRSS13	chr11:117789313-117789327	c.248(E2)至c.262(E2):缺失AGGCA>5<AGCCC	缺失非移码	rs201746372		杂合	可能相关	蛋白结构预测结果为有害		
MLKL	chr16:74712858	c.977C>G(E7)	p.326,P>R			杂合	可能相关	蛋白结构预测结果为有害		
SLC4A2	chr7:150763611	c.586G>A(E6)	p.196,V>M	rs537018427	0.0002	杂合	可能相关	蛋白结构预测结果为有害		
MRGPRX3	chr11:18159558	c.809T>A(E3)	p.270,I>N	rs183941944	0.0022	杂合	可能相关	蛋白结构预测结果为有害		
STARD9	chr15:42988052	c.13258G>C(E26)	p.4420,G>R	rs143402747	0.006	杂合	可能相关	蛋白结构预测结果为有害		
PAPPA	chr9:119158863	c.4852C>T(E22)	p.1618,R>W	rs373780391	0.0002	杂合	可能相关	蛋白结构预测结果为有害		

基因突变及良性多态性变异:

基因	染色体位置	核酸改变	氨基酸改变	rs编号	MAF	杂合/纯合	与疾病相关性	相关性说明	PubMed文献号	基因功能或关联疾病表型
ANKRD53	chr2:71211834-71211845	c.997(E6)至c.1008(E6):缺失GCCCGGGCCACC	缺失非移码	rs545687883	0.0022	杂合	可能相关	蛋白结构预测结果为有害		
KLHL8	chr4:88099704	c.1021A>G(E5)	p.341,I>V	rs76241124	0.0004	杂合	可能相关	蛋白结构预测结果为有害		
FAM179A	chr2:29274947	c.3048T>G(E20)	p.1016,F>L	rs77810069	0.013	纯合	可能相关	蛋白结构预测结果为有害		
SLC22A15	chr1:116609220	c.1445C>T(E11)	p.482,P>L	rs541129652	0.0002	杂合	可能相关	蛋白结构预测结果为有害		
NRBP1	chr2:27664043	c.1379A>G(E15)	p.460,H>R	rs34260196	0.0014	杂合	可能相关	蛋白结构预测结果为有害		
GREB1L	chr18:19034332	c.1990G>A(E15)	p.664,D>N	rs149534471	0.001	杂合	可能相关	蛋白结构预测结果为有害		
SPATA31A3	chr9:40703280	c.937G>C(E4)	p.313,E>Q	rs62565521		纯合	可能相关	蛋白结构预测结果为有害		
MBOAT7	chr19:54692157	c.28G>A(E2)	p.10,A>T	rs200505526	0.0012	杂合	可能相关	蛋白结构预测结果为有害		
MICALL1	chr22:38333116	c.2338C>T(E14)	p.780,R>W	rs575389316	0.0002	杂合	可能相关	蛋白结构预测结果为有害		
CAPRIN2	chr12:30866783	c.2684A>G(E16)	p.895,N>S			杂合	可能相关	蛋白结构预测结果为有害		
ANKRD40	chr17:48784934	c.82A>G(E1)	p.28,K>E	rs117204968	0.006	杂合	可能相关	蛋白结构预测结果为有害		
TECPR1	chr7:97847035	c.3353C>T(E25)	p.1118,P>L			杂合	可能相关	蛋白结构预测结果为有害		

基因突变及良性多态性变异:

基因	染色体位置	核酸改变	氨基酸改变	rs编号	MAF	杂合/纯合	与疾病相关性	相关性说明	PubMed文献号	基因功能或关联疾病表型
AFF3	chr2:100625314	c.134C>T(E4)	p.45,T>M			杂合	可能相关	蛋白结构预测结果为有害		
AFF4	chr5:132270203	c.554A>T(E3)	p.185,Q>L	rs137880283	0.0048	杂合	可能相关	蛋白结构预测结果为有害		
MEX3B	chr15:82336840	c.371G>T(E2)	p.124,R>L			杂合	可能相关	蛋白结构预测结果为有害		
OR1B1	chr9:125391653	c.162C>A(E1)	p.54,D>E	rs75769247	0.0046	杂合	可能相关	蛋白结构预测结果为有害		
MYO5C	chr15:52534310	c.2491C>T(E20)	p.831,R>C	rs191334982	0.0022	杂合	可能相关	蛋白结构预测结果为有害		
ELMOD2	chr4:141461350	c.428A>C(E6)	p.143,K>T	rs142450830	0.0058	杂合	可能相关	蛋白结构预测结果为有害		
ETV2	chr19:36133406	c.44C>T(E2)	p.15,P>L			杂合	可能相关	蛋白结构预测结果为有害		
FCGR1A	chr1:149755703	c.197C>T(E3)	p.66,S>L	rs144081076	0.0038	杂合	可能相关	蛋白结构预测结果为有害		
OR13C3	chr9:107298322	c.773T>C(E1)	p.258,M>T	rs80083218	0.008	杂合	可能相关	蛋白结构预测结果为有害		
NCAPH	chr2:97024911	c.1337G>A(E10)	p.446,R>H	rs202183416	0.0002	杂合	可能相关	蛋白结构预测结果为有害		
SPATA9	chr5:95011144	c.350C>T(E3)	p.117,A>V	rs547580466	0.0002	杂合	可能相关	蛋白结构预测结果为有害		
ANKRD36	chr2:97856878	c.2307C>G(E36)	p.769,N>K	rs187556886	0.002	杂合	可能相关	蛋白结构预测结果为有害		
STAT6	chr12:57496252	c.1333C>T(E13)	p.445,R>W			杂合	可能相关	蛋白结构预测结果为有害		

基因突变及良性多态性变异:

基因	染色体位置	核酸改变	氨基酸改变	rs编号	MAF	杂合/纯合	与疾病相关性	相关性说明	PubMed文献号	基因功能或关联疾病表型
CIC	chr19:42796852	c.3310T>A(E14)	p.1104,S>T	rs185048610	0.0038	杂合	可能相关	蛋白结构预测结果为有害		
FAM46A	chr6:82461727-82461728	c.131(E2)至c.132(E2):插入CGGCGACTTCGGCGG	插入非移码			杂合	可能相关	蛋白结构预测结果为有害		
KLC3	chr19:45853920-45853921	c.1294(E11)至c.1295(E11):缺失CT	p.432,L>Lfs5	rs528482467	0.002	杂合	较高	理论上这种移码突变会导致蛋白序列明显改变		
SPATA31A1	chr9:39360949	c.3187G>A(E4)	p.1063,A>T	rs62550833		纯合	可能相关	蛋白结构预测结果为有害		
SLC39A14	chr8:22272326	c.661G>A(E5)	p.221,V>I	rs142534611		杂合	可能相关	蛋白结构预测结果为有害		
RIN3	chr14:93151497	IVS9+2T>A	剪切位点			杂合	较高	突变在经典剪切位点上		
	chr14:93154538-93154540	c.2899(E10)至c.2901(E10):缺失GGC	缺失非移码	rs71698059		纯合	可能相关	蛋白结构预测结果为有害		
CR1L	chr1:207868022	c.788G>T(E5)	p.263,G>V	rs72468038	0.04	杂合	未知	蛋白结构预测结果为容忍或无害		
	chr1:207867854	c.620G>A(E5)	p.207,C>Y	rs41303261	0.039	杂合	可能相关	蛋白结构预测结果为有害		
	chr1:207851554	c.289C>T(E3)	p.97,R>C	rs72468037	0.04	杂合	可能相关	蛋白结构预测结果为有害		
AQPEP	chr5:115338957	c.1917A>T(E12)	p.639,Q>H			杂合	可能相关	蛋白结构预测结果为有害		

基因突变及良性多态性变异:

基因	染色体位置	核酸改变	氨基酸改变	rs编号	MAF	杂合/纯合	与疾病相关性	相关性说明	PubMed文献号	基因功能或关联疾病表型
SCAP	chr3:47455810	c.3454C>T(E22)	p.1152,R>W			杂合	可能相关	蛋白结构预测结果为有害		
ZFP37	chr9:115806359	c.539G>A(E4)	p.180,C>Y			杂合	可能相关	蛋白结构预测结果为有害		
	chr9:115812137	c.148G>C(E2)	p.50,D>H			杂合	可能相关	蛋白结构预测结果为有害		
IQCA1	chr2:237247013	c.1969C>T(E17)	p.657,R>C	rs186626813	0.0028	杂合	可能相关	蛋白结构预测结果为有害		
PSG1	chr19:43373030	c.866C>G(E4)	p.289,P>R	rs143886024	0.0076	杂合	可能相关	蛋白结构预测结果为有害		
LENG9	chr19:54973821	c.955C>T(E1)	p.319,R>C	rs114383943	0.0008	杂合	可能相关	蛋白结构预测结果为有害		
SNAPC3	chr9:15423046	c.169G>C(E1)	p.57,G>R	rs202082183	0.0016	杂合	可能相关	蛋白结构预测结果为有害		
FLOT2	chr17:27208326	c.982G>A(E9)	p.328,A>T	rs3736238	0.01	杂合	可能相关	蛋白结构预测结果为有害		
RRP36	chr6:42989434-42989451	c.42(E1)至c.59(E1):缺失CGGGG<8>CCCCG	缺失非移码	rs202053592	0.0086	杂合	未知	未知		
PCDHB8	chr5:140558102	c.487A>G(E1)	p.163,I>V	rs374425894	0.0004	杂合	可能相关	蛋白结构预测结果为有害		
FAM129B	chr9:130289567	c.221A>G(E3)	p.74,N>S	rs200797290	0.0006	杂合	可能相关	蛋白结构预测结果为有害		
SEMA3B	chr3:50306752-50306753	c.80(E2)至c.81(E2):插入C	p.27,S>Sfs36			纯合	较高	理论上这种移码突变会导致蛋白序列明显改变		

基因突变及良性多态性变异:

基因	染色体位置	核酸改变	氨基酸改变	rs编号	MAF	杂合/纯合	与疾病相关性	相关性说明	PubMed文献号	基因功能或关联疾病表型
LONP2	chr16:48330031	c.1489A>G(E9)	p.497,I>V			杂合	可能相关	蛋白结构预测结果为有害		
ATAD3B	chr1:1417587	c.584C>T(E6)	p.195,A>V	rs181312132	0.0046	杂合	可能相关	蛋白结构预测结果为有害		
CGREF1	chr2:27324354	c.745G>A(E6)	p.249,E>K	rs1057391		杂合	可能相关	蛋白结构预测结果为有害		
OR10A7	chr12:55615094	c.286G>A(E1)	p.96,G>S	rs12578318	0.008	杂合	可能相关	蛋白结构预测结果为有害		
LOC100996575	chr1:144619903	c.863T>A(E8)	p.288,V>E	rs71527379		纯合	未知	未知		
TTLL1	chr22:43465843	c.121G>A(E4)	p.41,V>M	rs547450706	0.0002	杂合	可能相关	蛋白结构预测结果为有害		
MTRNR2L2	chr5:79945871	c.35C>T(E1)	p.12,S>L	rs10942928		杂合	可能相关	蛋白结构预测结果为有害		
TTLL8	chr22:50483807	c.670C>T(E8)	p.224,R>C	rs201774397	0.0004	杂合	可能相关	蛋白结构预测结果为有害		
PCDHA9	chr5:140230097	c.2017G>C(E1)	p.673,G>R	rs17844334	0.0018	杂合	可能相关	蛋白结构预测结果为有害		
OR10A3	chr11:7960995	c.73G>T(E1)	p.25,V>L	rs539222840	0.0002	杂合	可能相关	蛋白结构预测结果为有害		
NRP2	chr2:206607968	c.1333A>C(E9)	p.445,I>L	rs201900948	0.0004	杂合	可能相关	蛋白结构预测结果为有害		
MTRNR2L8	chr11:10529739	c.35C>T(E1)	p.12,S>L	rs6484338		纯合	可能相关	蛋白结构预测结果为有害		
FUK	chr16:70512510	c.2886C>A(E22)	p.962,S>R	rs74613280	0.0096	杂合	可能相关	蛋白结构预测结果为有害		

基因突变及良性多态性变异:

基因	染色体位置	核酸改变	氨基酸改变	rs编号	MAF	杂合/纯合	与疾病相关性	相关性说明	PubMed文献号	基因功能或关联疾病表型
NOP16	chr5:175811094-175811095	c.586(E5)至c.587(E5):插入AT	p.196,R>Hfs38	rs56989856		杂合	较高	理论上这种移码突变会导致蛋白序列明显改变		
DESI2	chr1:244869041	c.535G>A(E5)	p.179,V>M			杂合	可能相关	蛋白结构预测结果为有害		
PCNXL2	chr1:233397813	c.458C>G(E3)	p.153,S>C			杂合	可能相关	蛋白结构预测结果为有害		
FAM90A1	chr12:8374781-8374782	c.1031(E7)至c.1032(E7):插入CGT	插入非移码	rs71265055		杂合	可能相关	蛋白结构预测结果为有害		
DENND1B	chr1:197643277	c.149A>T(E4)	p.50,K>M	rs76182569	0.0018	杂合	可能相关	蛋白结构预测结果为有害		
GPR152	chr11:67218797	c.1399G>A(E1)	p.467,A>T	rs199976078	0.0026	杂合	可能相关	蛋白结构预测结果为有害		
CELSR2	chr1:109812674	c.7227C>A(E23)	p.2409,H>Q	rs200084754	0.0016	杂合	可能相关	蛋白结构预测结果为有害		
	chr1:109808444	c.5815G>A(E14)	p.1939,E>K	rs184928378	0.0014	杂合	可能相关	蛋白结构预测结果为有害		
CCER1	chr12:91348057	c.463C>T(E1)	p.155,R>C	rs201558156	0.0006	杂合	可能相关	蛋白结构预测结果为有害		
C12orf49	chr12:117161005	c.135G>C(E2)	p.45,R>S	rs561038695	0.0004	杂合	可能相关	蛋白结构预测结果为有害		
OR4B1	chr11:48238422	c.61G>A(E1)	p.21,V>M	rs187430875	0.002	杂合	可能相关	蛋白结构预测结果为有害		
PMEL	chr12:56351128	c.959C>A(E7)	p.320,P>H	rs2071024	0.004	杂合	可能相关	蛋白结构预测结果为有害		
SRA1	chr5:139936980	c.58T>C(E1)	p.20,C>R	rs250427		杂合	可能相关	蛋白结构预测结果为有害		
TP53BP2	chr1:223991119	c.298C>A(E8)	p.100,Q>K	rs34683843	0.035	纯合	可能相关	蛋白结构预测结果为有害		

基因突变及良性多态性变异:

基因	染色体位置	核酸改变	氨基酸改变	rs编号	MAF	杂合/纯合	与疾病相关性	相关性说明	PubMed文献号	基因功能或关联疾病表型
PRR25	chr16:863422	c.770G>A(E3)	p.257,R>Q	rs142195552	0.0078	杂合	可能相关	蛋白结构预测结果为有害		
RSPH6A	chr19:46299138-46299139	c.2142(E6)至c.2143(E6):插入GAGGAGGAGGGCGAGGAG	插入非移码	rs3217398		杂合	可能相关	蛋白结构预测结果为有害		
LAMB4	chr7:107708575	c.2332G>A(E19)	p.778,G>R			杂合	可能相关	蛋白结构预测结果为有害		
FAM65A	chr16:67578623	c.2819G>A(E16)	p.940,R>H	rs145721165	0.002	杂合	可能相关	蛋白结构预测结果为有害		
SEMA5B	chr3:122642588	c.1148C>G(E10)	p.383,A>G	rs78693594	0.0024	杂合	可能相关	蛋白结构预测结果为有害		
ALS2CR8	chr2:203846865	c.1760C>T(E16)	p.587,P>L	rs75249727	0.0094	杂合	可能相关	蛋白结构预测结果为有害		
ADRBK1	chr11:67048250	c.551T>C(E7)	p.184,I>T	rs55696045	0.0004	杂合	可能相关	蛋白结构预测结果为有害		
AKAP13	chr15:86076886	c.253G>T(E4)	p.85,A>S	rs116551873	0.002	杂合	可能相关	蛋白结构预测结果为有害		
PRR14L	chr22:32109204	c.4621A>C(E4)	p.1541,I>L	rs144773326	0.006	杂合	可能相关	蛋白结构预测结果为有害		
SPATA31E1	chr9:90502058	c.2656G>C(E4)	p.886,E>Q	rs75241638	0.0064	杂合	可能相关	蛋白结构预测结果为有害		
ENPP2	chr8:120592406	c.1886A>G(E20)	p.629,N>S	rs2289886	0.005	杂合	可能相关	蛋白结构预测结果为有害		
	chr8:120629807	IVS5-4T>G	剪切位点	rs148588719	0.0052	杂合	可能相关	mRNA剪接可能会受影响		
ENPP5	chr6:46135587	c.413T>C(E2)	p.138,M>T	rs199876794	0.0014	杂合	可能相关	蛋白结构预测结果为有害		
C2orf57	chr2:232458767	c.1105G>A(E1)	p.369,V>M	rs201877259		杂合	可能相关	蛋白结构预测结果为有害		
EPS8L1	chr19:55594882	c.1351C>T(E13)	p.451,R>W			杂合	可能相关	蛋白结构预测结果为有害		
PDE4DIP	chr1:144852390	c.7053G>A(E44)	p.2351,W>X(12)	rs61804988		杂合	较高	无义突变可能会导致蛋白翻译提前终止		
	chr1:144886092	IVS23+5G>A	剪切位点	rs1343472		杂合	可能相关	mRNA剪接可能会受影响		

基因突变及良性多态性变异:

基因	染色体位置	核酸改变	氨基酸改变	rs编号	MAF	杂合/纯合	与疾病相关性	相关性说明	PubMed文献号	基因功能或关联疾病表型
TMEM184A	chr7:1586653-1586654	c.1176(E9)至c.1177(E9):插入GGC	插入非移码	rs374586451		纯合	可能相关	蛋白结构预测结果为有害		
MTF2	chr1:93602439	c.1637A>G(E15)	p.546,N>S	rs77900924	0.0038	杂合	可能相关	蛋白结构预测结果为有害		
PIAS3	chr1:145584490	c.1457C>T(E12)	p.486,T>I			杂合	可能相关	蛋白结构预测结果为有害		
C12orf70	chr12:27628640	c.488A>G(E5)	p.163,N>S	rs200524083	0.0006	杂合	可能相关	蛋白结构预测结果为有害		
TXNDC2	chr18:9887872	c.1396G>A(E2)	p.466,A>T	rs80251020	0.0042	杂合	可能相关	蛋白结构预测结果为有害		
	chr18:9887629	c.1153A>G(E2)	p.385,K>E	rs74368658	0.0026	杂合	可能相关	蛋白结构预测结果为有害		
LETM1	chr4:1843417	c.251C>G(E3)	p.84,S>W	rs202034038	0.0004	杂合	可能相关	蛋白结构预测结果为有害		
LILRB1	chr19:55148004	c.1707A>T(E15)	p.569,R>S	rs200490901		杂合	可能相关	蛋白结构预测结果为有害		
CARNS1	chr11:67186686	c.824G>A(E5)	p.275,R>H	rs181358688	0.003	杂合	可能相关	蛋白结构预测结果为有害		
FAM86B2	chr8:12291593	c.127G>T(E2)	p.43,D>Y	rs146321506		杂合	可能相关	蛋白结构预测结果为有害		
TMEM26	chr10:63170183	c.1004G>C(E6)	p.335,S>T			杂合	可能相关	蛋白结构预测结果为有害		
NUDT11	chrX:51239296-51239309	m.151(E1)至c.1(E1):缺失GGCTG>4<GAGGA	缺失移码			半合子	较高	起始密码子突变，影响蛋白编码起始位点		
NAV3	chr12:78444705	c.2294G>A(E11)	p.765,R>H			杂合	可能相关	蛋白结构预测结果为有害		
	chr12:78598728	c.6782G>A(E38)	p.2261,R>H			杂合	可能相关	蛋白结构预测结果为有害		
WNK3	chrX:54275950	c.2831G>T(E17)	p.944,G>V	rs190471786		半合子	可能相关	蛋白结构预测结果为有害		
NUDT15	chr13:48611918-48611919	c.36(E1)至c.37(E1):插入GGAGTC	插入非移码	rs554405994	0.016	杂合	可能相关	蛋白结构预测结果为有害		
RPUSD3	chr3:9885649	c.50G>T(E1)	p.17,R>L			杂合	可能相关	蛋白结构预测结果为有害		
SUCLG2	chr3:67426236	c.1231C>A(E11)	p.411,P>T	rs117743675	0.0002	杂合	可能相关	蛋白结构预测结果为有害		
POLK	chr5:74879226	c.1043A>G(E8)	p.348,D>G			杂合	可能相关	蛋白结构预测结果为有害		
OPTC	chr1:203472822	c.973C>T(E7)	p.325,R>W	rs56219555	0.0032	杂合	可能相关	蛋白结构预测结果为有害		
GTPBP2	chr6:43596838	c.62T>C(E1)	p.21,V>A	rs201099934	0.0014	杂合	可能相关	蛋白结构预测结果为有害		
CHRNA6	chr8:42611231	c.1111C>T(E5)	p.371,P>S	rs148005281	0.0014	杂合	可能相关	蛋白结构预测结果为有害		
VPS13D	chr1:12318057	c.1007G>A(E10)	p.336,R>H	rs143197573	0.002	杂合	可能相关	蛋白结构预测结果为有害		

基因突变及良性多态性变异:

基因	染色体位置	核酸改变	氨基酸改变	rs编号	MAF	杂合/纯合	与疾病相关性	相关性说明	PubMed文献号	基因功能或关联疾病表型
CD48	chr1:160650919-160650919	c.725(E3):缺失G	p.242,G>Vfs57	rs142100077	0.043	杂合	较高	理论上这种移码突变会导致蛋白序列明显改变		
HEMGN	chr9:100693387-100693389	c.288(E4)至c.290(E4):缺失AGT	缺失非移码	rs201990544	0.0028	杂合	可能相关	蛋白结构预测结果为有害		
HIBADH	chr7:27702373	c.35C>G(E1)	p.12,S>C			杂合	可能相关	蛋白结构预测结果为有害		
OR11H12	chr14:19378189	c.596G>T(E1)	p.199,R>L	rs2212201		杂合	未知	蛋白结构预测结果为容忍或无害		
SMPD2	chr6:109764222	c.667G>A(E8)	p.223,V>I	rs9386806	0.0076	杂合	可能相关	蛋白结构预测结果为有害		
IPPK	chr9:95420914	IVS2+2T>C	剪切位点			杂合	较高	突变在经典剪切位点上		
DENND4A	chr15:65989696	c.2856T>G(E21)	p.952,N>K			杂合	可能相关	蛋白结构预测结果为有害		
MEP1B	chr18:29784171	c.395G>A(E7)	p.132,R>Q	rs117084287	0.007	杂合	可能相关	蛋白结构预测结果为有害		
DENND4C	chr9:19332024	c.2302C>T(E17)	p.768,P>S	rs181354867	0.0012	杂合	未知	未知		
IQCF1	chr3:51928928	c.596T>C(E4)	p.199,I>T	rs142254753	0.0026	杂合	可能相关	蛋白结构预测结果为有害		
NDOR1	chr9:140110727	c.1529C>T(E13)	p.510,T>M	rs147308483	0.0058	杂合	可能相关	蛋白结构预测结果为有害		
ADI1	chr2:3517666	c.202A>G(E2)	p.68,T>A	rs57884381	0.0078	纯合	可能相关	蛋白结构预测结果为有害		
PEX11A	chr15:90226932	c.420T>A(E3)	p.140,Y>X(108)			杂合	较高	无义突变可能会导致蛋白翻译提前终止		
RCAN3	chr1:24840882	c.20A>G(E2)	p.7,K>R	rs143770846	0.0014	杂合	可能相关	蛋白结构预测结果为有害		
ASIC3	chr7:150747934-150747939	c.903(E4)至c.908(E4):缺失CCCCAG	缺失非移码	rs200386051		杂合	可能相关	蛋白结构预测结果为有害		

基因突变及良性多态性变异:

基因	染色体位置	核酸改变	氨基酸改变	rs编号	MAF	杂合/纯合	与疾病相关性	相关性说明	PubMed文献号	基因功能或关联疾病表型
ASIC2	chr17:31350878	IVS6+1G>A	剪切位点	rs199653464		杂合	较高	突变在经典剪切位点上		
NFRKB	chr11:129742837	c.2705C>G(E23)	p.902,P>R	rs200873091	0.0022	杂合	可能相关	蛋白结构预测结果为有害		
MORN1	chr1:2252973	c.1343T>C(E14)	p.448,L>P	rs201836187	0.0028	杂合	可能相关	蛋白结构预测结果为有害		
CNTD2	chr19:40729330	c.637G>A(E4)	p.213,G>R	rs373547599	0.0044	杂合	可能相关	蛋白结构预测结果为有害		
GPSM1	chr9:139235482-139235482	c.1239(E9):缺失C	p.414,P>Pfs56	rs374298038		杂合	较高	理论上这种移码突变会导致蛋白序列明显改变		
CEBPZ	chr2:37439494	c.2587G>A(E11)	p.863,D>N	rs544897966	0.0002	杂合	可能相关	蛋白结构预测结果为有害		
INPP5J	chr22:31522450	c.256G>A(E3)	p.86,D>N	rs150976596	0.0022	杂合	可能相关	蛋白结构预测结果为有害		
OR9I1	chr11:57886450	c.467C>A(E1)	p.156,A>D	rs199775374	0.0006	杂合	可能相关	蛋白结构预测结果为有害		
ALDH1A1	chr9:75540504	c.529A>T(E6)	p.177,I>F	rs8187929	0.0092	杂合	可能相关	蛋白结构预测结果为有害		
ARID2	chr12:46243406	c.1759A>G(E14)	p.587,S>G	rs78128744	0.0052	杂合	可能相关	蛋白结构预测结果为有害		
PKD1L1	chr7:47870905	c.6383C>A(E42)	p.2128,A>D	rs150761907	0.0006	杂合	可能相关	蛋白结构预测结果为有害		
	chr7:47983031	c.122T>A(E2)	p.41,L>Q	rs183524387	0.0028	杂合	可能相关	蛋白结构预测结果为有害		
ESYT2	chr7:158552793	c.1423C>G(E12)	p.475,L>V			杂合	可能相关	蛋白结构预测结果为有害		

基因突变及良性多态性变异:

基因	染色体位置	核酸改变	氨基酸改变	rs编号	MAF	杂合/纯合	与疾病相关性	相关性说明	PubMed文献号	基因功能或关联疾病表型
UPP2	chr2:158958551-158958552	IVS2-1至c.148(E3):插入A	插入移码	rs67998287		杂合	较高	理论上这种移码突变会导致蛋白序列明显改变		
ADAMTS15	chr11:130340817	c.1723T>G(E6)	p.575,S>A	rs79839754	0.0034	杂合	可能相关	蛋白结构预测结果为有害		
KDM6B	chr17:7752532-7752543	c.2926(E11)至c.2937(E11):缺失CAGAAGGAGCAT	缺失非移码	rs547582872	0.0024	杂合	未知	未知		
FHOD1	chr16:67264083	c.3100G>T(E20)	p.1034,V>F	rs146514223	0.014	杂合	可能相关	蛋白结构预测结果为有害		
ALDH9A1	chr1:165667711	c.85A>G(E1)	p.29,T>A	rs147998705	0.0012	杂合	可能相关	蛋白结构预测结果为有害		
ZFPM1	chr16:88599697-88599705	c.1331(E10)至c.1339(E10):缺失AGCCTCTGG插入CCC	复杂非移码	rs67712719,rs149145771,rs67322929,rs67873604		纯合	未知	蛋白结构预测结果为容忍或无害		
SPEF2	chr5:35753763	c.3368G>A(E24)	p.1123,R>Q	rs79487218	0.0048	纯合	可能相关	蛋白结构预测结果为有害		
TBCC	chr6:42712809	c.1003A>G(E1)	p.335,I>V	rs138350475	0.0018	杂合	可能相关	蛋白结构预测结果为有害		
LIMCH1	chr4:41699182	c.3232G>A(E27)	p.1078,G>R	rs142431467		杂合	可能相关	蛋白结构预测结果为有害		
FAM120C	chrX:54117740	IVS11+5G>T	剪切位点	rs2495797		半合子	可能相关	mRNA剪接可能会受影响		
CAPN12	chr19:39226831	c.1502T>A(E12)	p.501,L>Q	rs555930617	0.0004	杂合	可能相关	蛋白结构预测结果为有害		
SH2D3A	chr19:6755198	c.625G>A(E5)	p.209,A>T	rs76213282	0.0014	杂合	可能相关	蛋白结构预测结果为有害		
CHGA	chr14:93397655	c.416C>T(E6)	p.139,A>V	rs150148299	0.0004	杂合	可能相关	蛋白结构预测结果为有害		

基因突变及良性多态性变异:

基因	染色体位置	核酸改变	氨基酸改变	rs编号	MAF	杂合/纯合	与疾病相关性	相关性说明	PubMed文献号	基因功能或关联疾病表型
CHGA	chr14:93397654	c.415G>T(E6)	p.139,A>S	rs140821122	0.0004	杂合	可能相关	蛋白结构预测结果为有害		
TARBP1	chr1:234529446	c.4381A>G(E27)	p.1461,I>V	rs2275654	0.003	杂合	可能相关	蛋白结构预测结果为有害		
UFL1	chr6:96974255	c.409G>T(E5)	p.137,V>F	rs28372909	0.0066	杂合	可能相关	蛋白结构预测结果为有害		
CCNL2	chr1:1334613	c.74C>G(E1)	p.25,S>C	rs376963263	0.0006	杂合	可能相关	蛋白结构预测结果为有害		
NEK11	chr3:130828678	c.368A>G(E5)	p.123,Y>C	rs55806123	0.0042	杂合	可能相关	蛋白结构预测结果为有害		
SLC7A11	chr4:139093120-139093122	c.1494(E12)至c.1496(E12):缺失AGA	缺失非移码			杂合	可能相关	蛋白结构预测结果为有害		
TBCK	chr4:107154790	c.1467G>C(E16)	p.489,K>N	rs2305685	0.0006	杂合	可能相关	蛋白结构预测结果为有害		
MAN1A1	chr6:119623261	c.708C>G(E4)	p.236,I>M	rs201487763	0.0006	杂合	可能相关	蛋白结构预测结果为有害		
SIGLEC12	chr19:52001394	c.1283T>A(E5)	p.428,L>H	rs193032542	0.0022	杂合	可能相关	蛋白结构预测结果为有害		
ARID5B	chr10:63845597	c.1336C>T(E9)	p.446,H>Y	rs141586900	0.0006	杂合	可能相关	蛋白结构预测结果为有害		
SARS	chr1:109778650	c.1021T>C(E8)	p.341,F>L	rs143192294	0.0014	杂合	可能相关	蛋白结构预测结果为有害		
GAGE2A	chrX:49355893	c.175C>G(E3)	p.59,Q>E	rs59641550		半合子	可能相关	蛋白结构预测结果为有害		
CNTN6	chr3:1414127	c.1637A>G(E13)	p.546,K>R	rs145045076	0.0034	杂合	可能相关	蛋白结构预测结果为有害		
ACRBP	chr12:6752776	c.1006A>G(E6)	p.336,T>A	rs3741923	0.0038	杂合	可能相关	蛋白结构预测结果为有害		
CCDC108	chr2:219900320	c.424A>C(E5)	p.142,I>L	rs75663378	0.0072	杂合	可能相关	蛋白结构预测结果为有害		
ANKRD36B	chr2:98128313	c.3008C>T(E39)	p.1003,T>M	rs202131502		杂合	可能相关	蛋白结构预测结果为有害		
AKR1C1	chr10:5019931	c.969T>G(E9)	p.323,Y>X(1)	rs201500205	0.0016	杂合	较高	无义突变可能会导致蛋白翻译提前终止		
AP5M1	chr14:57741132	c.245C>T(E2)	p.82,S>F			杂合	可能相关	蛋白结构预测结果为有害		
PDE8A	chr15:85657252	c.1334T>C(E14)	p.445,V>A	rs200432446	0.0006	杂合	可能相关	蛋白结构预测结果为有害		
SCN7A	chr2:167262143	c.4996G>A(E25)	p.1666,G>S	rs138133378	0.0012	杂合	可能相关	蛋白结构预测结果为有害		
	chr1:16902884	c.1997A>G(E19)	p.666,N>S	rs74630591		杂合	可能相关	蛋白结构预测结果为有害		

基因突变及良性多态性变异:

基因	染色体位置	核酸改变	氨基酸改变	rs编号	MAF	杂合/纯合	与疾病相关性	相关性说明	PubMed文献号	基因功能或关联疾病表型
NBPF1	chr1:16901668	c.2176A>G(E20)	p.726,K>E	rs3901679		杂合	可能相关	蛋白结构预测结果为有害		
	chr1:16905718	c.1771T>G(E17)	p.591,C>G	rs3738661		杂合	可能相关	蛋白结构预测结果为有害		
C1orf159	chr1:1026914	c.10C>T(E3)	p.4,R>W			杂合	可能相关	蛋白结构预测结果为有害		
MAN2B2	chr4:6580124	c.290G>A(E3)	p.97,R>H	rs114307861	0.012	杂合	可能相关	蛋白结构预测结果为有害		
L1TD1	chr1:62676038	c.1592A>G(E5)	p.531,K>R			杂合	可能相关	蛋白结构预测结果为有害		
TOR3A	chr1:179063231	c.822T>A(E5)	p.274,N>K	rs201173574	0.0006	杂合	可能相关	蛋白结构预测结果为有害		
AFMID	chr17:76198639	c.214G>A(E3)	p.72,E>K	rs139139850	0.0026	杂合	可能相关	蛋白结构预测结果为有害		
ACOT4	chr14:74058668	c.5C>T(E1)	p.2,S>L	rs139476628	0.0066	杂合	可能相关	蛋白结构预测结果为有害		
CHPF2	chr7:150932675	c.805G>A(E2)	p.269,V>I			杂合	可能相关	蛋白结构预测结果为有害		
LYAR	chr4:4276124	c.802G>T(E7)	p.268,A>S	rs180853467	0.0024	杂合	可能相关	蛋白结构预测结果为有害		
APLF	chr2:68694870-68694870	c.7(E1):缺失G	p.3,G>Gfs22			杂合	较高	理论上这种移码突变会导致蛋白序列明显改变		
LTN1	chr21:30319854	c.3557A>C(E19)	p.1186,K>T			杂合	可能相关	蛋白结构预测结果为有害		
SDE2	chr1:226173217	c.1142A>G(E7)	p.381,D>G	rs76285584	0.0098	杂合	可能相关	蛋白结构预测结果为有害		
CPB2	chr13:46632343	c.970C>T(E9)	p.324,R>X(100)	rs183048225	0.001	杂合	较高	无义突变可能会导致蛋白翻译提前终止		
SLC13A3	chr20:45217838	c.836C>A(E8)	p.279,A>D			杂合	可能相关	蛋白结构预测结果为有害		
ZNF318	chr6:43306998	c.4738G>A(E10)	p.1580,A>T	rs3734684	0.0058	杂合	可能相关	蛋白结构预测结果为有害		
HIVEP3	chr1:42045428	c.5041C>T(E4)	p.1681,R>C	rs201327347	0.0002	杂合	可能相关	蛋白结构预测结果为有害		
HDHD3	chr9:116136473	c.162G>T(E2)	p.54,R>S	rs75660101	0.005	杂合	可能相关	蛋白结构预测结果为有害		

基因突变及良性多态性变异:

基因	染色体位置	核酸改变	氨基酸改变	rs编号	MAF	杂合/纯合	与疾病相关性	相关性说明	PubMed文献号	基因功能或关联疾病表型
C1orf127	chr1:11036248	c.92C>T(E2)	p.31,T>M	rs190590002	0.0006	杂合	可能相关	蛋白结构预测结果为有害		
ARMC9	chr2:232099979	c.665G>A(E8)	p.222,R>H	rs3752780	0.009	杂合	可能相关	蛋白结构预测结果为有害		
GPR39	chr2:133403080-133403081	c.1263(E2)至c.1264(E2):插入AAG	插入非移码	rs10631541	0.027	杂合	可能相关	蛋白结构预测结果为有害		
R3HDM2	chr12:57663729	c.1351G>A(E13)	p.451,G>S			杂合	可能相关	蛋白结构预测结果为有害		
SARM1	chr17:26699367-26699368	c.314(E1)至c.315(E1):插入C	p.105,L>Lfs51			杂合	较高	理论上这种移码突变会导致蛋白序列明显改变		
P2RX5	chr17:3593367	c.611C>G(E6)	p.204,S>C	rs376122191	0.0014	纯合	可能相关	蛋白结构预测结果为有害		
MUC4	chr3:195509029	c.9422G>A(E2)	p.3141,S>N			杂合	可能相关	蛋白结构预测结果为有害		
	chr3:195508956	c.9495G>C(E2)	p.3165,Q>H	rs9759028		杂合	可能相关	蛋白结构预测结果为有害		
CACNA2D3	chr3:55041821	c.2771A>G(E33)	p.924,Y>C	rs191332047	0.0004	杂合	可能相关	蛋白结构预测结果为有害		
SLC6A17	chr1:110717526	c.697G>A(E5)	p.233,V>M	rs79143268	0.002	杂合	可能相关	蛋白结构预测结果为有害		
CREBZF	chr11:85375300	c.620C>A(E1)	p.207,T>K	rs192968795	0.0022	杂合	可能相关	蛋白结构预测结果为有害		
GABRP	chr5:170236616	c.877C>T(E9)	p.293,R>C	rs79997355	0.015	杂合	可能相关	蛋白结构预测结果为有害		
	chr5:170221295	c.233G>C(E4)	p.78,S>T	rs142642795	0.008	杂合	可能相关	蛋白结构预测结果为有害		
MBNL3	chrX:131525119	IVS4-8G>A	剪切位点	rs72614187		半合子	可能相关	mRNA剪接可能会受影响		
DOPEY1	chr6:83847825	c.4037G>A(E21)	p.1346,R>Q	rs142639349	0.0034	杂合	可能相关	蛋白结构预测结果为有害		
OSBPL1A	chr18:21912965	c.566A>C(E7)	p.189,K>T			杂合	可能相关	蛋白结构预测结果为有害		
DPCR1	chr6:30919345	c.3104T>C(E2)	p.1035,I>T	rs566807233	0.0006	杂合	未知	蛋白结构预测结果为容忍或无害		
	chr6:30919296	c.3055G>A(E2)	p.1019,E>K	rs546915230	0.0002	杂合	可能相关	蛋白结构预测结果为有害		
CCL4	chr17:34431360	c.62C>T(E1)	p.21,A>V	rs143966312	0.0038	杂合	可能相关	蛋白结构预测结果为有害		
LRIG3	chr12:59313947	c.70G>A(E1)	p.24,A>T			杂合	可能相关	蛋白结构预测结果为有害		
FN3K	chr17:80706821	c.559C>T(E5)	p.187,R>X(123)			杂合	较高	无义突变可能会导致蛋白翻译提前终止		

基因突变及良性多态性变异:

基因	染色体位置	核酸改变	氨基酸改变	rs编号	MAF	杂合/纯合	与疾病相关性	相关性说明	PubMed文献号	基因功能或关联疾病表型
ABCA8	chr17:66914191	c.1924G>T(E14)	p.642,A>S	rs117020693	0.0086	杂合	可能相关	蛋白结构预测结果为有害		
NFE2L2	chr2:178095985	c.1346G>A(E5)	p.449,R>H	rs181294188	0.0004	杂合	可能相关	蛋白结构预测结果为有害		
FBXO15	chr18:71793304	c.590A>G(E6)	p.197,N>S	rs79499419	0.002	杂合	可能相关	蛋白结构预测结果为有害		
KIR3DL3	chr19:55241042	c.739C>T(E5)	p.247,R>W			杂合	可能相关	蛋白结构预测结果为有害		
KRBA1	chr7:149430826	c.2782G>A(E17)	p.928,A>T	rs547301232	0.0004	杂合	未知	未知		
HIF1A	chr14:62203623	c.1045G>A(E9)	p.349,D>N	rs142179458	0.0046	杂合	可能相关	蛋白结构预测结果为有害		
FAM48A	chr13:37583874-37583876	c.2273(E26)至c.2275(E26):缺失ATC	缺失非移码			杂合	未知	未知		
SIX4	chr14:61180672	c.1799C>T(E3)	p.600,S>L			杂合	可能相关	蛋白结构预测结果为有害		
VAX2	chr2:71148371	c.391G>A(E2)	p.131,E>K	rs200582274	0.0002	杂合	可能相关	蛋白结构预测结果为有害		
FLJ25363	chr3:109136723	c.185A>C(E2)	p.62,D>A	rs551919635	0.0026	杂合	可能相关	蛋白结构预测结果为有害		
NEIL3	chr4:178257369	c.521G>A(E4)	p.174,G>D			杂合	可能相关	蛋白结构预测结果为有害		
BPIFA3	chr20:31814763	c.649G>T(E6)	p.217,G>W	rs114904196	0.006	杂合	可能相关	蛋白结构预测结果为有害		
MTUS1	chr8:17510766	c.3313G>C(E12)	p.1105,E>Q	rs61733705	0.0066	杂合	可能相关	蛋白结构预测结果为有害		
ZNF574	chr19:42583753	c.995G>A(E2)	p.332,R>Q	rs3745226	0.0086	杂合	可能相关	蛋白结构预测结果为有害		
ELOVL7	chr5:60050522	c.775C>T(E9)	p.259,R>C	rs116939630	0.0074	杂合	可能相关	蛋白结构预测结果为有害		
DNAJC7	chr17:40134398	c.1106A>C(E11)	p.369,N>T	rs373239996	0.0002	杂合	可能相关	蛋白结构预测结果为有害		
PGLYRP3	chr1:153275063	c.550C>T(E5)	p.184,R>X(158)			杂合	较高	无义突变可能会导致蛋白翻译提前终止		
SND1	chr7:127729628	c.2506G>A(E22)	p.836,G>S	rs150524780	0.0044	杂合	可能相关	蛋白结构预测结果为有害		
UNC5A	chr5:176305466	IVS12-10T>A	剪切位点	rs2304516		纯合	可能相关	mRNA剪接可能会受影响		
AQP12B	chr2:241621869	c.386C>T(E1)	p.129,T>M	rs74882485		杂合	可能相关	蛋白结构预测结果为有害		
CHIC1	chrX:72783200-72783217	c.80(E1)至c.97(E1):缺失CGTCG<8>GCCGT	缺失非移码			半合子	未知	未知		
AGAP6	chr10:51748510	c.35G>A(E1)	p.12,S>N	rs372576093	0.0028	杂合	可能相关	蛋白结构预测结果为有害		

基因突变及良性多态性变异:

基因	染色体位置	核酸改变	氨基酸改变	rs编号	MAF	杂合/纯合	与疾病相关性	相关性说明	PubMed文献号	基因功能或关联疾病表型
ST3GAL1	chr8:134488126	c.142G>A(E4)	p.48,E>K			杂合	可能相关	蛋白结构预测结果为有害		
CGB7	chr19:49558216	c.65G>A(E2)	p.22,R>K	rs35728583		杂合	可能相关	蛋白结构预测结果为有害		
RGL3	chr19:11505146	c.2083G>A(E19)	p.695,A>T	rs149297688	0.0046	杂合	可能相关	蛋白结构预测结果为有害		
IDE	chr10:94297257	c.149T>C(E2)	p.50,I>T			杂合	可能相关	蛋白结构预测结果为有害		
XPO6	chr16:28123188	c.2249A>G(E18)	p.750,N>S	rs574144151	0.0002	杂合	可能相关	蛋白结构预测结果为有害		
METTL21A	chr2:208486581	c.208G>A(E3)	p.70,V>M	rs146834988	0.001	杂合	可能相关	蛋白结构预测结果为有害		
MICB	chr6:31477681	c.1147A>G(E6)	p.383,T>A			纯合	可能相关	蛋白结构预测结果为有害		
	chr6:31473546	c.223A>G(E2)	p.75,N>D			纯合	可能相关	蛋白结构预测结果为有害		
DNM1	chr9:130980957	c.332C>G(E3)	p.111,T>S	rs192913494	0.0042	杂合	可能相关	蛋白结构预测结果为有害		
FAM71E2	chr19:55870188	c.2048C>T(E9)	p.683,P>L	rs200752567	0.0004	杂合	可能相关	蛋白结构预测结果为有害		
USP42	chr7:6194295	c.3110A>G(E15)	p.1037,Y>C			杂合	可能相关	蛋白结构预测结果为有害		
PHLDB3	chr19:44006318	c.331C>T(E3)	p.111,R>C	rs147272153	0.0016	杂合	可能相关	蛋白结构预测结果为有害		
NLRP13	chr19:56443601	c.77A>G(E1)	p.26,Q>R	rs76565431	0.0068	杂合	可能相关	蛋白结构预测结果为有害		
NLRP14	chr11:7059878	c.61G>A(E2)	p.21,E>K	rs11041150	0.0062	杂合	可能相关	蛋白结构预测结果为有害		
CMTM7	chr3:32433531	c.133G>T(E1)	p.45,A>S			杂合	可能相关	蛋白结构预测结果为有害		
DGKZ	chr11:46400758	c.3109C>G(E30)	p.1037,P>A			杂合	可能相关	蛋白结构预测结果为有害		
NARFL	chr16:782297	IVS8+4C>T	剪切位点			杂合	可能相关	mRNA剪接可能会受影响		
GAS2L2	chr17:34079859	c.11C>T(E1)	p.4,P>L			杂合	可能相关	蛋白结构预测结果为有害		
CDH18	chr5:19612540	IVS6+3A>G	剪切位点	rs188184405	0.0004	杂合	可能相关	mRNA剪接可能会受影响		
C6orf10	chr6:32307382	c.383C>T(E10)	p.128,P>L			纯合	可能相关	蛋白结构预测结果为有害		
IGSF22	chr11:18735537	c.1957C>T(E14)	p.653,R>C	rs138337552	0.0008	杂合	可能相关	蛋白结构预测结果为有害		

基因突变及良性多态性变异:

基因	染色体位置	核酸改变	氨基酸改变	rs编号	MAF	杂合/纯合	与疾病相关性	相关性说明	PubMed文献号	基因功能或关联疾病表型
IGSF21	chr1:18554492	c.171C>G(E2)	p.57,I>M	rs368924241		杂合	可能相关	蛋白结构预测结果为有害		
RASSF4	chr10:45467247	c.89C>T(E3)	p.30,T>I	rs184086507	0.0028	杂合	可能相关	蛋白结构预测结果为有害		
TNP2	chr16:11362979	c.141G>C(E1)	p.47,Q>H	rs201198575	0.0006	杂合	可能相关	蛋白结构预测结果为有害		
TRIM22	chr11:5719719	c.694A>G(E4)	p.232,T>A	rs2291843	0.0098	杂合	可能相关	蛋白结构预测结果为有害		
WWTR1	chr3:149243847	c.971C>T(E6)	p.324,P>L			杂合	可能相关	蛋白结构预测结果为有害		
STAB2	chr12:104111587	c.4651A>G(E44)	p.1551,K>E	rs185812040	0.0006	杂合	可能相关	蛋白结构预测结果为有害		
TMPRSS7	chr3:111766749-111766749	c.516(E5):缺失C	p.173,C>Vfs 6	rs144239192	0.006	杂合	较高	理论上这种移码突变会导致蛋白序列明显改变		
ERVFRD-1	chr6:11105086	c.458C>G(E2)	p.153,S>C			杂合	可能相关	蛋白结构预测结果为有害		
GPR78	chr4:8584263	c.674G>A(E2)	p.225,R>Q			杂合	可能相关	蛋白结构预测结果为有害		
ZNF24	chr18:32920512	c.103G>A(E2)	p.35,E>K			杂合	可能相关	蛋白结构预测结果为有害		
NBPF11	chr1:146057292	c.320A>G(E5)	p.107,N>S	rs201615164		杂合	可能相关	蛋白结构预测结果为有害		
OR2A12	chr7:143792742	c.542T>C(E1)	p.181,M>T	rs148680931	0.0008	杂合	可能相关	蛋白结构预测结果为有害		
NBPF16	chr1:148754942	c.1598A>T(E14)	p.533,Q>L	rs201022397		杂合	可能相关	蛋白结构预测结果为有害		
NBPF14	chr1:148010911	c.1711C>T(E14)	p.571,R>C	rs61810210		杂合	可能相关	蛋白结构预测结果为有害		
KIAA1551	chr12:32137854	c.3965G>A(E4)	p.1322,R>Q	rs118160668	0.0006	杂合	可能相关	蛋白结构预测结果为有害		
	chr12:32137833	c.3944A>G(E4)	p.1315,E>G	rs202170401	0.0002	杂合	可能相关	蛋白结构预测结果为有害		
TTC12	chr11:113236975	c.2071G>A(E22)	p.691,G>S	rs138333675	0.0034	杂合	可能相关	蛋白结构预测结果为有害		
PHLDA1	chr12:76424615	c.907G>A(E1)	p.303,V>I	rs78921231	0.006	杂合	可能相关	蛋白结构预测结果为有害		
	chr12:76424938-76424940	c.582(E1)至c.584(E1):缺失GCA	缺失非移码	rs71716769	0.0094	杂合	可能相关	蛋白结构预测结果为有害		
ZBP1	chr20:56186819	c.838G>A(E6)	p.280,E>K	rs536878834		杂合	可能相关	蛋白结构预测结果为有害		

基因突变及良性多态性变异:

基因	染色体位置	核酸改变	氨基酸改变	rs编号	MAF	杂合/纯合	与疾病相关性	相关性说明	PubMed文献号	基因功能或关联疾病表型
CDRT4	chr17:15341322	c.227C>T(E4)	p.76,P>L	rs184570389	0.0008	杂合	未知	蛋白结构预测结果为容忍或无害		
KNDC1	chr10:134980927	c.145G>A(E2)	p.49,G>S			杂合	可能相关	蛋白结构预测结果为有害		
FBXW10	chr17:18647637	c.80G>A(E1)	p.27,R>Q	rs200553727	0.0002	杂合	可能相关	蛋白结构预测结果为有害		
PET112	chr4:152679989	c.262C>T(E2)	p.88,R>C	rs202123480	0.0006	杂合	可能相关	蛋白结构预测结果为有害		
SERPINB12	chr18:61228352	c.419C>T(E4)	p.140,T>M	rs534052662	0.0002	杂合	可能相关	蛋白结构预测结果为有害		
TEKT5	chr16:10721553	c.1345C>T(E7)	p.449,R>W	rs61731534	0.0062	杂合	可能相关	蛋白结构预测结果为有害		
USP37	chr2:219374744	c.983C>G(E11)	p.328,P>R	rs150878701	0.0076	杂合	可能相关	蛋白结构预测结果为有害		
REXO1	chr19:1818484	c.3013C>T(E10)	p.1005,R>W	rs77403452	0.0022	杂合	可能相关	蛋白结构预测结果为有害		
FAM166A	chr9:140140000	c.281C>T(E3)	p.94,P>L	rs77069806	0.0014	杂合	可能相关	蛋白结构预测结果为有害		
FAM166B	chr9:35563312	c.137G>A(E2)	p.46,R>Q	rs374415795		杂合	可能相关	蛋白结构预测结果为有害		
CRISPLD2	chr16:84922968	c.1438A>G(E14)	p.480,S>G			杂合	可能相关	蛋白结构预测结果为有害		
PAAF1	chr11:73638343	c.1064A>T(E13)	p.355,E>V			杂合	可能相关	蛋白结构预测结果为有害		
QTRT1	chr19:10818225	c.580A>G(E5)	p.194,K>E	rs188490696	0.0008	杂合	可能相关	蛋白结构预测结果为有害		
NHLRC2	chr10:115644050	c.950T>C(E5)	p.317,I>T	rs536340589	0.0004	杂合	可能相关	蛋白结构预测结果为有害		
RABGAP1	chr9:125761051	IVS10+6G>A	剪切位点	rs605546		杂合	可能相关	mRNA剪接可能会受影响		
PHLDB1	chr11:118509676	c.2603G>A(E12)	p.868,R>H	rs2298484	0.0098	杂合	可能相关	蛋白结构预测结果为有害		
MFSD7	chr4:676591	c.1240G>A(E9)	p.414,E>K	rs187312561	0.0014	杂合	可能相关	蛋白结构预测结果为有害		
KIAA1429	chr8:95523796	c.3007A>G(E13)	p.1003,I>V	rs200661878	0.0002	杂合	可能相关	蛋白结构预测结果为有害		
CBWD6	chr9:69206924	c.864A>T(E12)	p.288,E>D	rs62553375		杂合	可能相关	蛋白结构预测结果为有害		
	chr9:69238234	c.658C>A(E8)	p.220,L>I	rs66626885		杂合	可能相关	蛋白结构预测结果为有害		
KIAA1217	chr10:24508723	c.239G>C(E2)	p.80,G>A			杂合	可能相关	蛋白结构预测结果为有害		
GLRA4	chrX:102979223	c.277C>T(E4)	p.93,R>W			半合子	可能相关	蛋白结构预测结果为有害		
ZNF480	chr19:52825077	c.574C>T(E5)	p.192,Q>X(344)			杂合	较高	无义突变可能会导致蛋白翻译提前终止		
SPRR3	chr1:152975963	c.467C>T(E3)	p.156,T>M	rs2075740	0.0022	杂合	可能相关	蛋白结构预测结果为有害		
NUP205	chr7:135300818	IVS24+5G>C	剪切位点	rs74594925	0.018	杂合	可能相关	mRNA剪接可能会受影响		
CLIC3	chr9:139889452	c.482G>A(E5)	p.161,R>H	rs371960731	0.003	杂合	可能相关	蛋白结构预测结果为有害		
GOLGA6L10	chr15:82636963	c.1123C>T(E6)	p.375,R>C	rs550639504	0.037	杂合	可能相关	蛋白结构预测结果为有害		
BRWD1	chr21:40570769	c.5573A>T(E40)	p.1858,Q>L	rs147211854	0.0022	杂合	可能相关	蛋白结构预测结果为有害		

基因突变及良性多态性变异:

基因	染色体位置	核酸改变	氨基酸改变	rs编号	MAF	杂合/纯合	与疾病相关性	相关性说明	PubMed文献号	基因功能或关联疾病表型
ST6GALNAC1	chr17:74625371-74625373	c.552(E2)至c.554(E2):缺失CCA	缺失非移码	rs565363235	0.0018	杂合	可能相关	蛋白结构预测结果为有害		
CCT6A	chr7:56128100	c.1204A>G(E10)	p.402,I>V	rs192970041	0.009	杂合	可能相关	蛋白结构预测结果为有害		
PLEKHH1	chr14:68053942	c.4085C>T(E29)	p.1362,T>M	rs201241121	0.0012	杂合	可能相关	蛋白结构预测结果为有害		
CCDC178	chr18:30969588	c.124G>A(E4)	p.42,A>T	rs12606658	0.0092	杂合	可能相关	蛋白结构预测结果为有害		
ZDHHC8	chr22:20128413	c.772C>T(E7)	p.258,R>W	rs374745817		杂合	可能相关	蛋白结构预测结果为有害		
FMNL1	chr17:43318566	c.1301G>A(E13)	p.434,S>N	rs118179136	0.0012	杂合	可能相关	蛋白结构预测结果为有害		
TCHH	chr1:152081116	c.4577G>C(E2)	p.1526,R>P	rs185664717	0.0086	杂合	可能相关	蛋白结构预测结果为有害		
ANGPT4	chr20:870992	c.329C>T(E2)	p.110,T>M	rs142196008	0.0002	杂合	可能相关	蛋白结构预测结果为有害		
BRD4	chr19:15349608	c.3966C>G(E19)	p.1322,D>E	rs371743668		杂合	可能相关	蛋白结构预测结果为有害		
FOXB2	chr9:79635474	c.904G>A(E1)	p.302,V>M	rs200620036	0.0016	杂合	可能相关	蛋白结构预测结果为有害		
CEACAM18	chr19:51981879	c.166G>A(E2)	p.56,E>K	rs76009806	0.0048	杂合	可能相关	蛋白结构预测结果为有害		
SPIB	chr19:50926128	c.173A>G(E4)	p.58,Y>C	rs145129604		杂合	可能相关	蛋白结构预测结果为有害		
SPANXD	chrX:140785792	c.124C>A(E2)	p.42,L>I	rs149669480		半合子	可能相关	蛋白结构预测结果为有害		
SYNJ2	chr6:158454680	c.679G>A(E4)	p.227,G>S	rs147847428	0.0016	杂合	可能相关	蛋白结构预测结果为有害		
TMEM102	chr17:7340260	c.962T>G(E3)	p.321,V>G	rs567937828	0.0006	杂合	可能相关	蛋白结构预测结果为有害		
SERPINA4	chr14:95033457	c.800G>A(E3)	p.267,R>Q	rs191488393	0.0002	杂合	可能相关	蛋白结构预测结果为有害		
NMUR2	chr5:151784190	c.485G>A(E1)	p.162,R>Q	rs137937656	0.0002	杂合	可能相关	蛋白结构预测结果为有害		
ZNF683	chr1:26689533	c.1127G>A(E5)	p.376,R>Q	rs202155950	0.0006	杂合	可能相关	蛋白结构预测结果为有害		
EVI5	chr1:93159927	c.1060C>T(E8)	p.354,R>C	rs117711462	0.0008	杂合	可能相关	蛋白结构预测结果为有害		
CILP2	chr19:19653379	c.788T>C(E5)	p.263,M>T			杂合	可能相关	蛋白结构预测结果为有害		
SMAP1	chr6:71567717	c.1054G>C(E10)	p.352,A>P	rs117107973	0.0016	杂合	可能相关	蛋白结构预测结果为有害		
CFHR2	chr1:196920061-196920064	c.333(E3)至c.336(E3):缺失AATT	p.112,I>Ffs18	rs375905519	0.0034	杂合	较高	理论上这种移码突变会导致蛋白序列明显改变		
DDI1	chr11:103907858	c.308G>C(E1)	p.103,G>A	rs115094910	0.006	杂合	可能相关	蛋白结构预测结果为有害		
KIAA1614	chr1:180904367	c.1322C>T(E5)	p.441,P>L	rs144390511	0.0048	杂合	可能相关	蛋白结构预测结果为有害		
ZNF155	chr19:44501145	c.1136A>G(E5)	p.379,K>R	rs2302411	0.0084	杂合	可能相关	蛋白结构预测结果为有害		
ZNF295	chr21:43412698	c.1507G>A(E3)	p.503,G>R	rs200969667	0.002	杂合	可能相关	蛋白结构预测结果为有害		
CACHD1	chr1:65099758	c.668G>A(E7)	p.223,R>Q	rs199888610	0.0006	杂合	可能相关	蛋白结构预测结果为有害		

基因突变及良性多态性变异:

基因	染色体位置	核酸改变	氨基酸改变	rs编号	MAF	杂合/纯合	与疾病相关性	相关性说明	PubMed文献号	基因功能或关联疾病表型
ATP10B	chr5:160016649	c.3700A>G(E24)	p.1234,I>V	rs187173360	0.0018	杂合	可能相关	蛋白结构预测结果为有害		
	chr5:159992771	c.4075C>A(E26)	p.1359,P>T	rs151210844	0.0046	杂合	可能相关	蛋白结构预测结果为有害		
ZNF19	chr16:71510045-71510046	c.404(E6)至c.405(E6):插入TT	p.135,V>Vfs 11	rs77495348		杂合	较高	理论上这种移码突变会导致蛋白序列明显改变		
PLCXD2	chr3:111432752	c.643T>C(E3)	p.215,C>R	rs76407344	0.0062	杂合	可能相关	蛋白结构预测结果为有害		
RBMXL3	chrX:114424650	c.646A>G(E1)	p.216,I>V	rs372019163		半合子	可能相关	蛋白结构预测结果为有害		
MAP2	chr2:210570359	c.4640T>C(E11)	p.1547,I>T			杂合	可能相关	蛋白结构预测结果为有害		
CMYA5	chr5:78985833	c.103G>A(E1)	p.35,E>K	rs141830505	0.0056	杂合	可能相关	蛋白结构预测结果为有害		
HEBP2	chr6:138725714	c.82C>G(E1)	p.28,P>A			杂合	可能相关	蛋白结构预测结果为有害		
KCNJ8	chr12:21926210-21926224	c.327(E2)至c.341(E2):缺失AATGG>5<AGTGG	缺失非移码			杂合	未知	未知		
WDR66	chr12:122359397-122359398	c.186(E2)至c.187(E2):插入GAGGAGGAGGAGAAA	插入非移码	rs142042908		杂合	未知	蛋白结构预测结果为容忍或无害		
CCDC81	chr11:86119225	c.1026T>G(E9)	p.342,S>R	rs200061603	0.001	杂合	可能相关	蛋白结构预测结果为有害		
PAIP1	chr5:43543191	c.649A>T(E4)	p.217,M>L	rs200898020	0.0002	杂合	可能相关	蛋白结构预测结果为有害		
MKI67	chr10:129906928	c.3176C>T(E13)	p.1059,A>V	rs61729196	0.013	杂合	可能相关	蛋白结构预测结果为有害		
	chr10:129899952	c.9275G>A(E14)	p.3092,R>H	rs189424344	0.001	杂合	可能相关	蛋白结构预测结果为有害		
GUCY2F	chrX:108647653	c.2029G>C(E10)	p.677,V>L	rs35474112		半合子	可能相关	蛋白结构预测结果为有害		
	chrX:108718478	c.688C>T(E2)	p.230,R>W	rs33973457		半合子	可能相关	蛋白结构预测结果为有害		
	chrX:108696981	c.1140G>T(E4)	p.380,Q>H	rs2272925		半合子	可能相关	蛋白结构预测结果为有害		
ZC3H13	chr13:46584543	c.686C>T(E7)	p.229,S>L			杂合	可能相关	蛋白结构预测结果为有害		
DVL2	chr17:7133695	c.319C>T(E3)	p.107,R>W			杂合	可能相关	蛋白结构预测结果为有害		
POTED	chr21:14987871	c.790G>T(E3)	p.264,D>Y	rs55653693		杂合	可能相关	蛋白结构预测结果为有害		
	chr21:14987811	c.730C>T(E3)	p.244,H>Y	rs56121372		杂合	可能相关	蛋白结构预测结果为有害		
	chr21:15013670	c.1538C>T(E11)	p.513,S>F	rs544959599	0.0004	杂合	可能相关	蛋白结构预测结果为有害		

基因突变及良性多态性变异:

基因	染色体位置	核酸改变	氨基酸改变	rs编号	MAF	杂合/纯合	与疾病相关性	相关性说明	PubMed文献号	基因功能或关联疾病表型
IQSEC1	chr3:12942851-12942851	c.2977(E14):缺失G	p.993,P>Hfs127	rs56387830		杂合	较高	理论上这种移码突变会导致蛋白序列明显改变		
ZNF70	chr22:24087185	c.143A>G(E2)	p.48,Y>C	rs145952310	0.0042	杂合	可能相关	蛋白结构预测结果为有害		
	chr22:24086120	c.1208G>C(E2)	p.403,R>P	rs202042749		杂合	可能相关	蛋白结构预测结果为有害		
FGL1	chr8:17739546	c.206A>G(E4)	p.69,N>S	rs201668151	0.0004	杂合	可能相关	蛋白结构预测结果为有害		
DNMT3L	chr21:45681109	c.34G>A(E2)	p.12,E>K	rs75396112		杂合	可能相关	蛋白结构预测结果为有害		
POTEM	chr14:20002286	c.1136T>C(E7)	p.379,L>S			杂合	可能相关	蛋白结构预测结果为有害		
CCDC102B	chr18:66504029	c.29T>C(E4)	p.10,I>T	rs528717742	0.0008	杂合	可能相关	蛋白结构预测结果为有害		
CXorf65	chrX:70325901	c.199C>T(E3)	p.67,L>F	rs184815664		半合子	可能相关	蛋白结构预测结果为有害		
GOLGA6B	chr15:72956802	c.1451A>G(E13)	p.484,Q>R	rs202197805		纯合	可能相关	蛋白结构预测结果为有害		
POTEH	chr22:16269934	c.1247T>C(E7)	p.416,L>S	rs2845206		杂合	可能相关	蛋白结构预测结果为有害		
OR56B4	chr11:6129919	c.911G>T(E1)	p.304,R>I			杂合	可能相关	蛋白结构预测结果为有害		
C20orf173	chr20:34117097	c.106C>T(E2)	p.36,R>X(167)	rs141795719	0.0018	杂合	较高	无义突变可能会导致蛋白翻译提前终止		
KIAA1009	chr6:84913772	c.614C>T(E7)	p.205,P>L			杂合	可能相关	蛋白结构预测结果为有害		
C1orf56	chr1:151020478	c.155C>T(E1)	p.52,A>V	rs138867928	0.0026	杂合	可能相关	蛋白结构预测结果为有害		
SCRIB	chr8:144890866	c.2028G>T(E15)	p.676,E>D			杂合	可能相关	蛋白结构预测结果为有害		
PRRC2A	chr6:31604011	c.5650G>A(E26)	p.1884,G>S	rs9366785	0.027	杂合	可能相关	蛋白结构预测结果为有害		
UBA5	chr3:132394144	c.865C>G(E9)	p.289,Q>E			杂合	可能相关	蛋白结构预测结果为有害		
GSTCD	chr4:106640376	c.586C>T(E3)	p.196,R>C			杂合	可能相关	蛋白结构预测结果为有害		
MTX2	chr2:177193087	c.352G>A(E6)	p.118,V>I	rs78943740	0.0078	杂合	可能相关	蛋白结构预测结果为有害		
DNAH14	chr1:225534194	c.10855T>C(E69)	p.3619,C>R	rs12565381	0.0006	杂合	可能相关	蛋白结构预测结果为有害		
TNRC18	chr7:5434111	c.303C>A(E3)	p.101,N>K			杂合	可能相关	蛋白结构预测结果为有害		
PPP2R3A	chr3:135722253	c.1913C>T(E2)	p.638,S>L	rs141649812	0.0024	杂合	可能相关	蛋白结构预测结果为有害		

基因突变及良性多态性变异:

基因	染色体位置	核酸改变	氨基酸改变	rs编号	MAF	杂合/纯合	与疾病相关性	相关性说明	PubMed文献号	基因功能或关联疾病表型
DNAH10	chr12:124393994	IVS57+5G>C	剪切位点			杂合	可能相关	mRNA剪接可能会受影响		
IRAK1BP1	chr6:79577384	c.91A>G(E1)	p.31,R>G	rs191756094	0.0032	杂合	可能相关	蛋白结构预测结果为有害		
UNC13C	chr15:54307777	c.2677A>G(E1)	p.893,T>A	rs548329395	0.0002	杂合	可能相关	蛋白结构预测结果为有害		
FAM18B2	chr17:15406413	c.596A>G(E6)	p.199,Q>R			杂合	可能相关	蛋白结构预测结果为有害		
PRMT10	chr4:148605101	c.38G>C(E1)	p.13,G>A	rs554850100	0.001	杂合	可能相关	蛋白结构预测结果为有害		
GGACT	chr13:101184775	c.71C>A(E2)	p.24,A>D	rs148679908	0.0096	杂合	可能相关	蛋白结构预测结果为有害		
AQR	chr15:35210529	c.1272G>A(E15)	p.424,M>I			杂合	可能相关	蛋白结构预测结果为有害		
OGFOD2	chr12:123463486	c.538G>A(E7)	p.180,E>K			杂合	可能相关	蛋白结构预测结果为有害		
DCDC1	chr11:31349808	c.20A>G(E3)	p.7,E>G	rs11031357	0.0072	杂合	可能相关	蛋白结构预测结果为有害		
SSPO	chr7:149518533-149518533	c.12616(E83):缺失C	p.4206,C>Afs38	rs11353848		杂合	较高	理论上这种移码突变会导致蛋白序列明显改变		
SPIRE2	chr16:89894998	c.40G>A(E1)	p.14,A>T	rs571363661	0.0054	杂合	可能相关	蛋白结构预测结果为有害		
PKHD1L1	chr8:110463357	c.6329C>T(E41)	p.2110,T>I	rs202241413	0.0006	杂合	可能相关	蛋白结构预测结果为有害		
LIG3	chr17:33319051-33319052	c.1283(E7)至c.1284(E7):缺失AT	p.428,H>Rfs6			杂合	较高	理论上这种移码突变会导致蛋白序列明显改变		
LIG1	chr19:48657168	c.427G>C(E6)	p.143,D>H			杂合	可能相关	蛋白结构预测结果为有害		
ZNF646	chr16:31088280	c.635A>G(E2)	p.212,Q>R			杂合	可能相关	蛋白结构预测结果为有害		
TPSB2	chr16:1279622	c.178G>A(E3)	p.60,G>R	rs370837354	0.0002	杂合	可能相关	蛋白结构预测结果为有害		
BAHCC1	chr17:79410575	c.2029G>A(E4)	p.677,A>T	rs189830738	0.0014	杂合	未知	未知		
YEATS2	chr3:183439751	c.364T>A(E5)	p.122,S>T			杂合	可能相关	蛋白结构预测结果为有害		

基因突变及良性多态性变异:

基因	染色体位置	核酸改变	氨基酸改变	rs编号	MAF	杂合/纯合	与疾病相关性	相关性说明	PubMed文献号	基因功能或关联疾病表型
MED26	chr19:16687570	c.1071G>C(E3)	p.357,K>N			杂合	可能相关	蛋白结构预测结果为有害		
ADAM2	chr8:39613406	c.1638A>G(E16)	p.546,I>M	rs78400740	0.0014	杂合	可能相关	蛋白结构预测结果为有害		
NAA11	chr4:80247016	c.16G>A(E1)	p.6,A>T	rs3811765	0.0016	杂合	可能相关	蛋白结构预测结果为有害		
ANKLE1	chr19:17394502	c.929C>T(E5)	p.310,P>L	rs564336131	0.0002	杂合	可能相关	蛋白结构预测结果为有害		
PGC	chr6:41709537	c.692G>T(E6)	p.231,S>I	rs74742298	0.0046	杂合	可能相关	蛋白结构预测结果为有害		
SLC9A4	chr2:103090268	c.50T>C(E1)	p.17,L>P	rs79378995	0.0096	杂合	可能相关	蛋白结构预测结果为有害		
PLEKHM2	chr1:16056399	c.2183T>C(E14)	p.728,F>S			杂合	可能相关	蛋白结构预测结果为有害		
HIGD1C	chr12:51354842	c.186G>A(E2)	p.62,M>I	rs145639855	0.0026	杂合	可能相关	蛋白结构预测结果为有害		
FLYWCH1	chr16:2980847	c.759C>G(E4)	p.253,S>R			杂合	可能相关	蛋白结构预测结果为有害		
