# Supplementary figures and images for: Clinical Manifestations of Alport Syndrome-Diffuse Leiomyomatosis Patients With Contiguous Gene Deletions in COL4A6 and COL4A5
Source: Front Med (Lausanne). 2021 Oct 27;8:766224. doi: 10.3389/fmed.2021.766224 (PMC8578185; doi:10.3389/fmed.2021.766224)

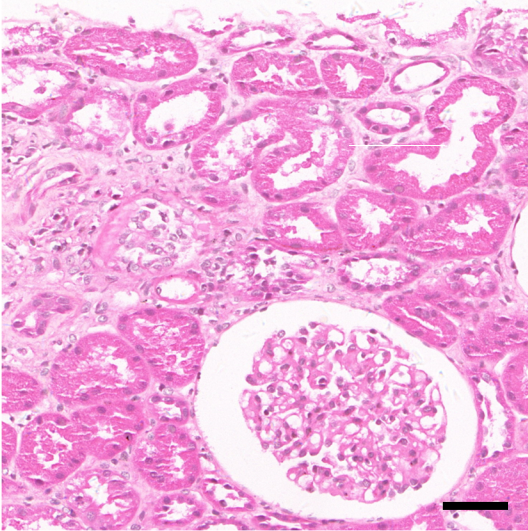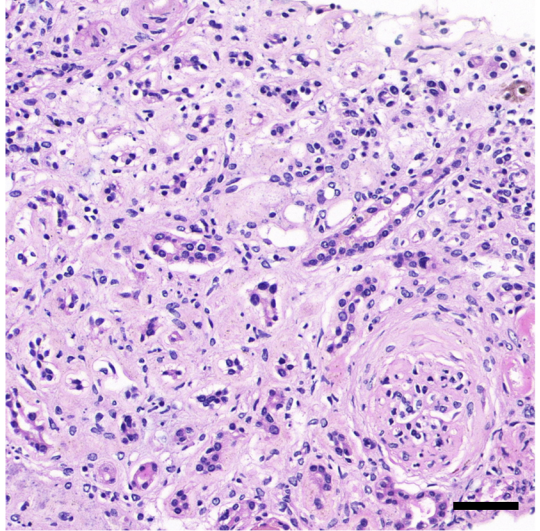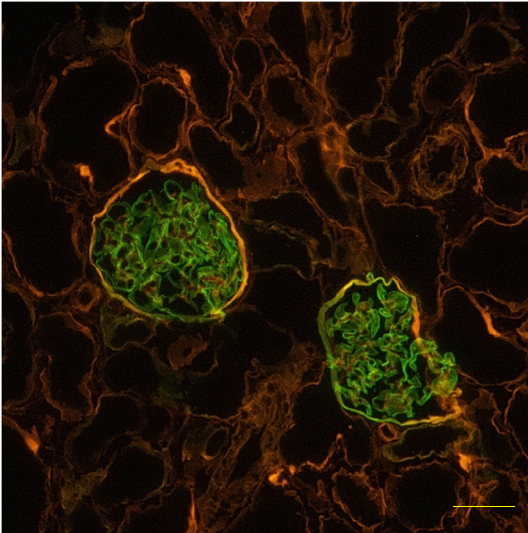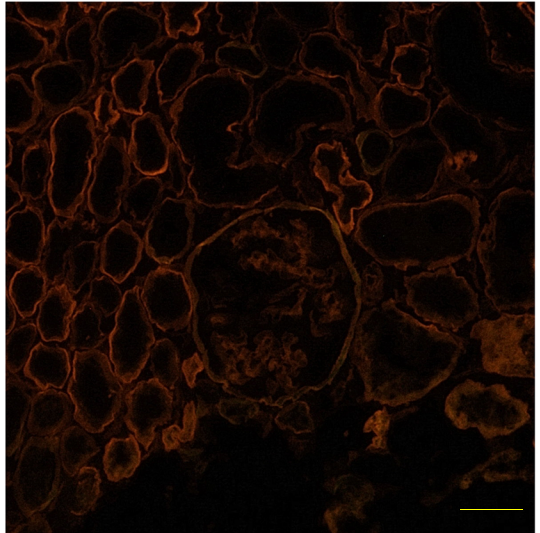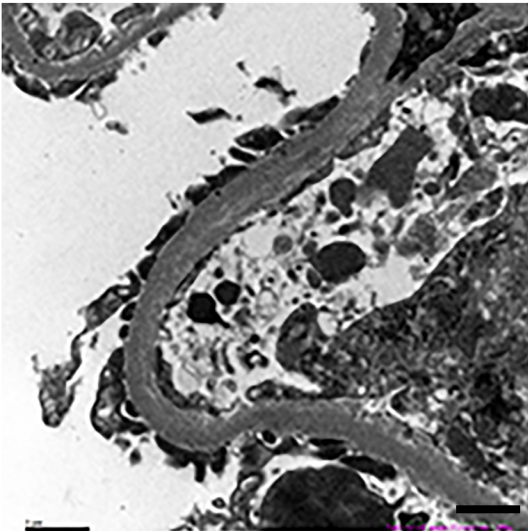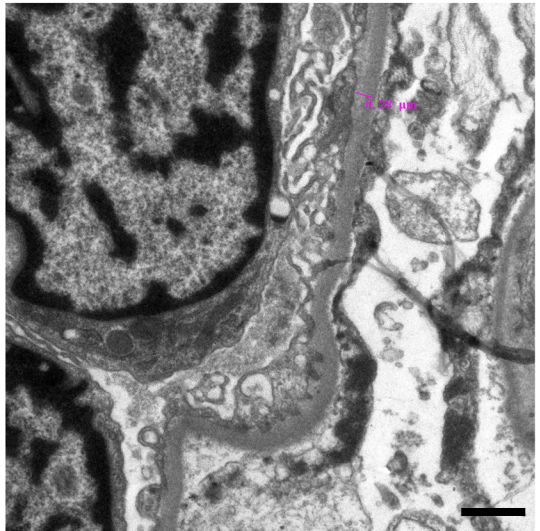

Supplement: Supplementary file 7 [file Data_Sheet_3.PDF]
